# Schlafen 12 Slows TNBC Tumor Growth, Induces Luminal Markers, and Predicts Favorable Survival

**DOI:** 10.3390/cancers15020402

**Published:** 2023-01-07

**Authors:** Sandeep K. Singhal, Sarmad Al-Marsoummi, Emilie E. Vomhof-DeKrey, Bo Lauckner, Trysten Beyer, Marc D. Basson

**Affiliations:** 1 Department of Pathology, School of Medicine and the Health Sciences, University of North Dakota, Grand Forks, ND 58202, USA; 2 Department of Biomedical Sciences, School of Medicine and the Health Sciences, University of North Dakota, Grand Forks, ND 58202, USA; 3 Department of Surgery, School of Medicine and the Health Sciences, University of North Dakota, Grand Forks, ND 58202, USA

**Keywords:** prognostic predictor, personalized medicine, xenograft, RNA sequencing, race

## Abstract

**Simple Summary:**

This study indicates that we could develop a prognostic predictor and new targets for TNBC treatment by increasing the expression levels of the protein SLFN12 and through targeting the SLFN12 gene signature. Therapies that would target SLFN12 and its corresponding gene signature could lead to a reduced tumor growth, an increased differentiation of breast cancer cells with luminal markers, and an increased survival of patients. Additionally, our results could be applied to providing personalized targeted therapy for African Americans, who are at a higher risk for TNBCs and a worse prognosis.

**Abstract:**

The Schlafen 12 (SLFN12) protein regulates triple-negative breast cancer (TNBC) growth, differentiation, and proliferation. SLFN12 mRNA expression strongly correlates with TNBC patient survival. We sought to explore SLFN12 overexpression effects on in vivo human TNBC tumor xenograft growth and performed RNA-seq on xenografts to investigate related SLFN12 pathways. Stable SLFN12 overexpression reduced tumorigenesis, increased tumor latency, and reduced tumor volume. RNA-seq showed that SLFN12 overexpressing xenografts had higher luminal markers levels, suggesting that TNBC cells switched from an undifferentiated basal phenotype to a more differentiated, less aggressive luminal phenotype. SLFN12-overexpressing xenografts increased less aggressive BC markers, HER2 receptors ERBB2 and EGFR expression, which are not detectable by immunostaining in TNBC. Two cancer progression pathways, the NAD signaling pathway and the superpathway of cholesterol biosynthesis, were downregulated with SLFN12 overexpression. RNA-seq identified gene signatures associated with SLFN12 overexpression. Higher gene signature levels indicated good survival when tested on four independent BC datasets. These signatures behaved differently in African Americans than in Caucasian Americans, indicating a possible biological difference between these races that could contribute to the worse survival observed in African Americans with BC. These results suggest an increased SLFN12 expression modulates TNBC aggressiveness through a gene signature that could offer new treatment targets.

## 1. Introduction

There are 290,560 new cases and 43,870 deaths due to breast cancer in the United States alone annually [1]. Although we have learned much about breast cancer, there is still much to be done to understand and treat the disease. Schlafen 12 (SLFN12) is a protein-coding gene that has a reduced expression in human triple-negative breast cancer (TNBC) tumors and correlates with survival [2]. This study sought to further investigate the role of SLFN12 in breast cancer.

Schlafen 12 (SLFN12) is a member of the Schlafen protein family, which is widely expressed across species including both humans and rodents, although humans express different SLFNs than rodents. These proteins are subclassified into short, intermediate, and long SLFNs based on size and structure [3]. SLFN12 and its close relation SLFN12-like protein are the only human intermediate SLFN proteins and notably lack the nuclear targeting sequence that localizes long SLFNs to the nucleus [4]. Indeed, adding a nuclear exclusion sequence to the mouse ortholog Slfn3 does not appear to interfere with its activity [5]. SLFN12 has previously been associated with various diseases and pathways, including lung adenocarcinoma, triple-negative breast cancer, prostate cancer, T cell activation, and human enterocytic differentiation [2,3,4,6,7,8]. 

SLFN12 appears to act through diverse pathways. For instance, SLFN12 reduces TNBC cell line proliferation and invasiveness while promoting differentiation and reducing the proportion of the cancer stem-cell-like subpopulation. This occurs at least in part because SLFN12 decreases the translation and promotes the degradation of ZEB1, a transcription factor that promotes TNBC stem cell differentiation [2]. SLFN12 also affects the sensitivity of TNBC cells to radiation and to some but not all chemotherapeutic agents [9]. Alternatively, SLFN12 promotes enterocytic differentiation by binding to Serpin B12 and altering its interaction with critical deubiquitylases that affect the degradation of phenotype-driving transcription factors such as Cdx2 [4]. Others have focused on SLFN12 interaction with PDE3A, an enzyme that hydrolyzes cAMP and cGMP [6]. SLFN12 and PDE3A form a complex through the binding of DNMDP to PDE3A [10]. When DNMDP binds to PDE3A, the surface of PDE3A becomes an adhesive surface for SLFN12 [11]. Estrogen-like hormones induce apoptosis through this mechanism when SLFN12 binds to ribosomes in the cell, instigated by the formation of the PDE3A–SLFN12 complex [12]. This process blocks signal recognition molecules from binding to the ribosome and leads to a block in protein translation occurring in the endoplasmic reticulum [12]. Additionally, SLFN12 may be an RNase. Its activity is essential for cancer cell killing induced by velcrins, a proposed class of molecules like DNMDP with adhesive properties [11]. 

Because SLFN12 acts in many ways, and because cancers frequently develop downstream mutations to escape from potential tumor suppressors such as SLFN12, it may be important to investigate not just SLFN12 but its downstream and associated genes. This study, therefore, sought to define an SLFN12-associated gene set and to characterize the relationship of such a gene set to survival in breast cancer. We established control and SLFN12-overexpressing human breast cancer xenografts from MDA-MB-231 cells exposed to an empty vector or an SLFN12-encoding lentivirus and assessed the behavior of such xenografts. Then, we used xenograft tumor bulk RNA-seq data to investigate pathways related to SLFN12 in breast cancer and used a statistical analysis to develop a gene signature. Finally, we used this gene signature to create a prediction model for human breast cancer based on survival and race.

This study thus exemplifies the use of using different machine learning and statistical modeling approaches applied to demographic, and multiple type of different genomic data, including RNA-seq, single-cell sequencing and microarray data to develop a risk predictor breast cancer biomarker. We translated the findings from one data platform to another using various statistical and machine learning methods. For instance, the biomarkers identified using the RNA-seq xenograft data were then translated to microarray data from human samples. Collection, transformation, management, and intelligent data integration systems were used to develop an SLFN12-based breast cancer risk predictor. Machine learning approaches such as principal component analysis (PCA), correlation, regression model, and survival analysis based upon optimal cutoff methods were used at various points throughout the study. 

## 2. Materials and Methods

### 2.1. Animal Study

#### 2.1.1. Animal Care

All procedures involving animals were conducted in conformity with the guidelines of the University of North Dakota Institutional Animal Care and Use Committee (protocol#1912-1). Animals were housed at the University of North Dakota Center for Biomedical Research facility and were fed and watered according to institutional guidelines. Animals were euthanized using an approved protocol.

#### 2.1.2. Animals

The NOD-*scid* IL2Rgamma^null^ mouse model (NOD.Cg-*Prkdc^scid^ Il2rg^tm1Wjl^*/SzJ), also known as NSG, was used in this study. Animals were supplied by Jackson Laboratory (Bar Harbor, ME, USA). These mice carry the severe combined immune deficiency (scid) and a complete null allele of the IL2 receptor common gamma chain (IL2rg^null^) mutations on the NOD/ShiLtJ genetic background. These mice are B- and T-deficient from the scid mutation that is in the DNA repair complex protein Prkdc. They also have NK cells that are functionally deficient from the IL2rg^null^ mutation, which prevents cytokine signaling through many receptors.

#### 2.1.3. Animal Surgery

MDA-MB-231 cells stably overexpressing SLFN12 and control cells (generated in our laboratory as described previously [9]) were cultured in DMEM medium supplemented with 10% FBS at 37 °C and 5% CO_2_, passed twice and allowed to reach 70% confluence. We denoted the control cells as “EV” and the SLFN12-overexpressing cells as “LV”. On the day of surgery, cells were detached with 0.25% trypsin, and resuspended in sterile PBS. Viable cells were counted using a Spark^®^ Multimode Microplate Reader by Tecan (Männedorf, Switzerland). Animals were injected with buprenorphine (1–2 mg/kg) subcutaneously for pain control, anesthetized with 2% isoflurane, and shaved. A 2–4 mm incision was made lateral to the midline on the abdominal skin adjacent to the right fourth nipple. The fourth mammary fat pad was identified, retracted using forceps, and injected with 100 µL of cell suspension (350,000 cells/100 µL of PBS) using a sterile 25-gauge needle. The skin was sutured with 3–0 Perma-hand silk thread (Ethicon, Raritan, New Jersey) and the mouse was allowed to recover from the anesthesia. Animals were monitored for a full recovery and returned to the cage with free access to water and food. During the first 24 h of postsurgery, animals were checked every three hours for any sign of distress, bleeding, or wound dehiscence. Skin sutures were removed after 7–10 days postoperative. Animals were then checked every three days for the development of a palpable tumor. The first day at which the tumor became palpable was recorded, and the subsequent tumor size was measured with a digital caliper every three days in addition to monitoring animal weight and welfare. Tumor volume was calculated using the formula tumor volume = (W × W × L)/2, with the (W) width diameter being smaller than the (L) length diameter [13,14,15]. Animals were followed for 12 weeks after surgery (the approved duration of the protocol), during which any animal that met the criteria of tumor burden or was critically ill was euthanized. The day of the first palpable tumor was recorded as the onset of the tumor to be used in the analysis for the latency of tumor development. For RNA sequencing, mice were euthanized, and the xenograft tumors were carefully excised and washed three times with PBS. A 10 mg piece from the xenograft tumor was rapidly excised and preserved separately for the qPCR analysis. Xenograft tumors were then snap-frozen in liquid nitrogen and stored at −80 °C prior to shipment on dry ice to GENEWIZ (South Plainfield, NJ) for sequencing.

#### 2.1.4. RNA and RT-qPCR

For xenograft tumors, 10 mg of tissue was snap-frozen in liquid nitrogen. Then, 1 mL of QIAzol lysis reagent (#79306, Qiagen, Hilden, Germany) was added together with one tungsten carbide bead (#69997, Qiagen) and homogenized over four minutes with a TissueLyser (Qiagen) at 40 Hz. Then, 400 µL of chloroform was added with gentle shaking, followed by centrifugation at 12,000× *g* for 15 min at 4 °C. A quantity of 600 µL of the upper aqueous clear layer was then transferred to a new 2 mL tube, and RNA was extracted using a QIAcube (RNeasy Lipid Protocol, Qiagen) using an RNeasy Mini Kit (Qiagen) according to the manufacturer’s protocol. cDNA was prepared from RNA samples using the SMARTScribe Reverse Transcription kit (#639537, Takara Clontech, Mountain View, CA, USA) as per the manufacturer’s protocol. cDNA samples were analyzed by a multiplexing qPCR analysis using the BioRad CFX96 Touch Real-Time PCR Detection System and the PrimeTime Gene Expression Master Mix (#1055772, IDT, Coralville, IA, USA). Expression levels were determined from the threshold cycle (Cq) values using the method of 2^−∆∆Ct^ using RPLP0 as the reference control gene. The following primer–probe sets were from IDT: FBP1-forward 5′-AGC AGT CAA AGC CAT CTC TTC-3′, reverse 5′-ACG TCC AGC TTC TTA ACT TGA-3′, probe 5′-/5Cy5/CAA TGC CAT AGA GGT GCG CGA TG/3IAbRQSp/-3′; EEF1A2-forward 5′-CAC CCA GGC ATA CTT GAA GG-3′, reverse 5′-GCC ACC TCA TCT ACA AAT GC-3′, probe 5′-/56-FAM/CCG CCT CCT/ZEN/TCT CGA ACT TCT CAA T/3IABkFQ/; GJA1-forward 5′-GTA CTG ACA GCC ACA CCT TC-3′, reverse 5′-ACT TGG CGT GAC TTC ACT AC-3′, probe 5′-/5Cy5/AGG CAA CAT GGG TGA CTG GAG C/3IAbRQSp/-3′; UCA1-forward 5′-ATC AGT CTT CAG CCA CTA AGC-3′, reverse 5′-TGA AGA GAT CCA CCT GCG A-3′, probe 5′-/56-FAM/CCT CAG ACC/ZEN/AGC CCA AGG AAC ATC/3IABkFQ/-3′. B2M-forward 5′-GGA CTG GTC TTT CTA TCT CTT GT-3′, reverse 5′-ACC TCC ATG ATG CTG CTT AC-3′, probe 5′-/FAM/CCT GCC GTG/ZEN/TGA ACC ATG TGA CT/3IABkFQ/-3′. The following primer–probe sets were from BioRad and are proprietary: CALB2 (Cy5.5, Assay ID qHsaCIP0031558), PAEP (Cy5.5, Assay ID qHsaCEP0024781), NQO1 (Tex615, Assay ID qHsaCEP0039593), GJB3 (Tex615, Assay ID qHsaCEP0055265). The statistical analysis performed was an unpaired, nonparametric, Mann–Whitney *t*-test. 

#### 2.1.5. Genomics

Libraries were prepared using the Tecan Ovation SoLo RNA-Seq library preparation kit as per the manufacturer’s instructions. Libraries were quantified using the Quant-iT PicoGreen dsDNA assay kit and read on a BioTek FLx800 fluorescence microplate reader. Libraries were pooled equal molar and the sample pool was quantified using Biorad ddPCR. Libraries were sequenced as stated below. 

### 2.2. Study Population

#### Xenograft Samples

A single-cell and bulk-RNA sequencing dataset was generated by Genewiz, Inc. (South Plainfield, NJ, USA). The bulk-RNA sequencing dataset comprised six samples, of which three had normal expressions of SLFN12, and the remaining three had an overexpression of SLFN12. Similarly, single-cell sequencing consisted of two samples, of which one had normal expression, and the other had an overexpression of SLFN12. After receiving the raw files (FASTQ) from Genewiz, preprocessing and normalization was performed in-house using the following approach.

### 2.3. Bulk-RNA Data Preprocessing

RNA-seq raw reads were stored in fastq format files containing the sequence data. We explored the fastq files to visualize preliminary results. In addition, we accessed the quality of reads using FastQC to check if any filtration of the raw reads was required [16]. The QC report of the datasets was assessed, and only qualified samples were included. Trimmomatic v.0.36 was used to eliminate potential adaptor sequences and low-quality nucleotides from sequencing reads. Using the STAR aligner v.2.5.2b, the trimmed reads were mapped to the *Homo sapiens* GRCh38 reference genome available on ENSEMBL [17]. The STAR aligner is a spliced aligner that recognizes and includes splice junctions to aid in aligning whole read sequences. As a result of this procedure, BAM files were created [18]. Using the feature Counts from the Subread package v.1.5.2, we computed the number of unique gene hits. The gene ID feature in the annotation file was used to summarize and provide the hit numbers. Special readings that fell within exon regions were counted [19]. 

#### Human Samples

Separate from xenograft samples, SLFN12 expression and its associated gene signatures were validated in human breast cancers to find clinical relevance. For this purpose, we utilized our data repository, which is a collection of data from collaborators and public resources of early breast cancer studies with survival and the race information [20,21,22,23,24] (Supplementary Table S1). An outline of the methods in this study is shown in a flowchart (Supplementary Figure S1).

### 2.4. Statistical Methods

A two-sided *t*-test was performed to investigate the significant difference genes between EV-Control and LV-SLFN12. Together with the SLFN12 gene alone, a few gene signatures were developed to better understand the relationship of SLFN12 with survival and race using the significant genes across a phenotypic condition. Scattered volcano plots were used to show the statistical significance with *p*-value versus the magnitude of change |fold-change|. To avoid the loss of a significant number of genes at the initial level without further examination, genes were filtered based on a *p*-value with a threshold of 0.05 without statistical approaches that govern the false discovery rate (FDR).

### 2.5. Functional and Pathway Analysis

A STRING functional enrichment analysis [25] was used to determine the functional association among significantly up- and downregulated genes. The network was created using known interactions from curated databases, experimentally determined relations, and text mining from online databases. The minimum required interaction score was set to the highest confidence, i.e., 0.900, and the connecting lines representing the interaction sources used different colors.

Genes were ranked according to their log2 fold changes. Ranked lists were used in a gene set enrichment analysis (GSEA) preranked software tool for both minimum gene set sizes of 5 and 50 with a maximum gene set size of 500 [26]. The MSigDB [27] collected was used to identify enriched gene sets, including Hallmark, C1, C2, C3, C4, C5, C6, C7, and C8. Enrichment plots were generated using the GSEA.

A QIAGEN Ingenuity Pathway Analysis (IPA) was used to study gene expression data in-depth and deduce connect relevant pathways. The z-score of IPA indicates whether a pathway is projected to be activated or inhibited, with a negative z value indicating inhibition and a positive z value indicating activation. |z| values greater than 2 were considered significant [28].

### 2.6. Single-Cell Analysis

A single-cell analysis was performed in 10x Genomics’s Loupe Browser 4.1.0. Single-cell data were clustered by K-means into four groups, and cell types were identified using breast cancer cell types from Gambardella et al. [29]. The selection of four K-means clusters was based on minimizing the number of clusters to compare the intensity and number of cells in the clusters. Significant genes per cluster were found in Loupe Browser through a globally distinguishing significant feature comparison including the log2 fold change as the displayed numeric value, features filtered by all significant genes per cluster, and all gene expression exported. Genes with *p* < 0.05 were then selected. Violin plots corresponding to cluster expressions were produced in Loupe Browser.

### 2.7. Computation of Signature (Module) Score for High SLFN12 Expression Biomarker

The module score was calculated as Module Score (s) = $\frac{\sum_{icn}W_{i}X_{i}}{\sum_{icn}\left|W_{i}\right|}$ where *n* is the number of genes in the module, *X_i_* is the gene’s expression, and gene-specific weights *W_i_* are equal to +1 or −1 depending on the direction of their correlation with the phenotype in the original publication where the module score was introduced. Only genes with Entrez Gene IDs were used in this study. Within our research, each module score was scaled so that the 2.5 percent and 97.5 percent quantiles were −1 and +1, respectively [30].

Four gene signatures were defined as a part of this study. SLFN12_Sig was defined as including all the significant genes differently expressed between normal vs. high SLFN12 expression with the magnitude and direction. SLFN12_Sig_NoDir was defined as including all the significant genes without consideration for the direction of regulation across phenotypic condition. SLFN12_Sig_Up and SLFN12_Sig_Dn was defined as including only genes that were upregulated and downregulated in high SLFN12 compared to control.

For the survival analysis, to classify the low vs. high gene signature value, optimal cut-off points for RNA expression/signature scores were determined as previously described by Hothorn et al. [31] and plotted using the Kaplan–Meier curve. The significance of individual hazard ratios was estimated by Wald’s test.

### 2.8. Data Availability 

The data generated in this study are publicly available in Gene Expression Omnibus (GEO) at GSE215442. 

## 3. Results

### 3.1. Schlafen 12 Overexpression Reduces the Growth of Mammary Xenograft Tumors

**Figure 1:**
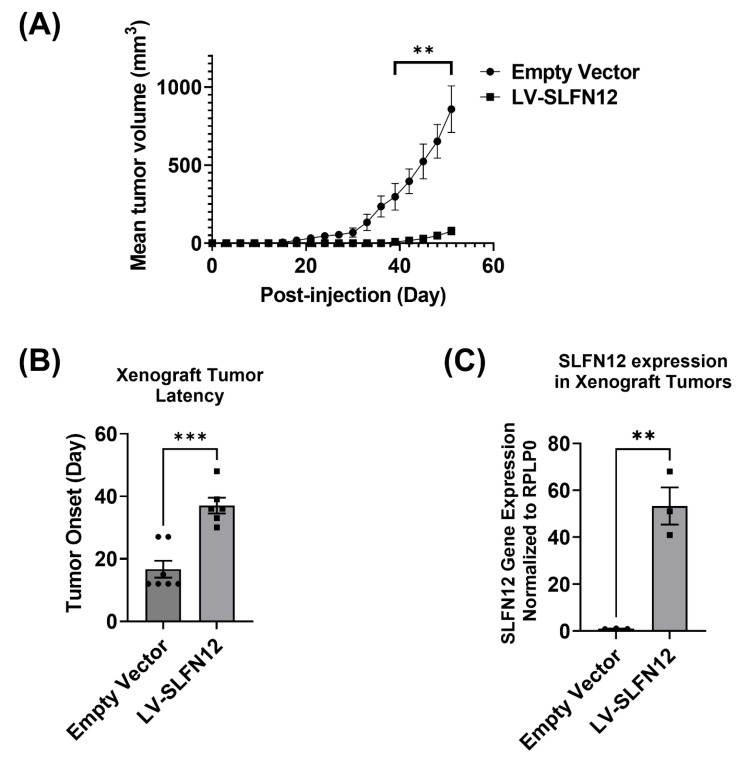
Schlafen 12 reduces mammary xenograft tumor growth in vivo. (**A**) Average tumor volume as a function of time in NSG mice that were orthotopically injected with MDA-MB-231 cells overexpressing SLFN12 (LV-SLFN12) compared to NSG mice injected with control MDA-MB-231 cells (empty vector) over a 12-week period (n = 6, ** *p* ≤ 0.01, student *t*-test). (**B**) NSG mice injected with MDA-MB-231 cells overexpressing SLFN12 (LV-SLFN12) showed significantly increased latency compared to mice injected with control empty vector cells (n = 6, ** *p* ≤ 0.01, student *t*-test). (**C**) mRNA analysis by Primer-probe RT-qPCR of SLFN12 expressions in LV-SLFN12 mammary xenograft tumors that were processed for RNA-seq analysis compared to the control empty vector mammary xenograft tumor; RPLP0 was used as a reference gene (n = 3, ** *p* ≤ 0.01, *** *p* ≤ 0.001). Data are expressed as mean ± SEM.

Animals that were orthotopically injected with MDA-MB-231 cells overexpressing SLFN12 (LV-SLFN12) exhibited a substantially reduced tumorigenesis as indicated by a slower tumor growth over a period of twelve weeks and an increased tumor latency compared to animals injected with the control MDA-MB-231 cells that had been treated with an empty vector lentivirus (EV). Tumors in mice that had been injected with LV-SLFN12 cells were substantially smaller tumors than those in mice that had been injected with the control empty vector cells (EV) (Figure 1A). On the 42nd day after injection, for instance, the mean tumor volume of LV-SLFN12 animals was 17.98 mm^3^ ± 7.51 versus 396.1 ± 79.03 mm^3^ in control empty vector xenografts (*p* < 0.01, n = 6). The mean tumor volume 51 days after of LV-SLFN12 xenografts was 76.64 ± 26.09 mm^3^ versus 858.8 ± 149.4 mm^3^ in empty vector tumors (n = 6, *p* < 0.001) (Figure 1A). Mice injected with LV-SLFN12 also exhibited a substantially greater mammary xenograft tumor latency (the time interval between injection and the day at which the tumor became palpable) (Figure 1B). The mean latency of mammary xenograft tumor in LV-SLFN12-injected mice was 37.0 ± 2.53 days versus 19.13 ± 3.35 days in mice injected with EV (n = 6, *p* < 0.01) (Figure 1B). At the end of the study, mammary xenograft tumors of LV-SLFN12 demonstrated a higher SLFN12 expression (53.32 ± 7.92-fold versus 1.01 ± 0.07-fold in empty vector tumor, n = 3, *p* < 0.01) (Figure 1C).

### 3.2. Global Gene Expression Analysis for Xenografts

**Figure 2:**
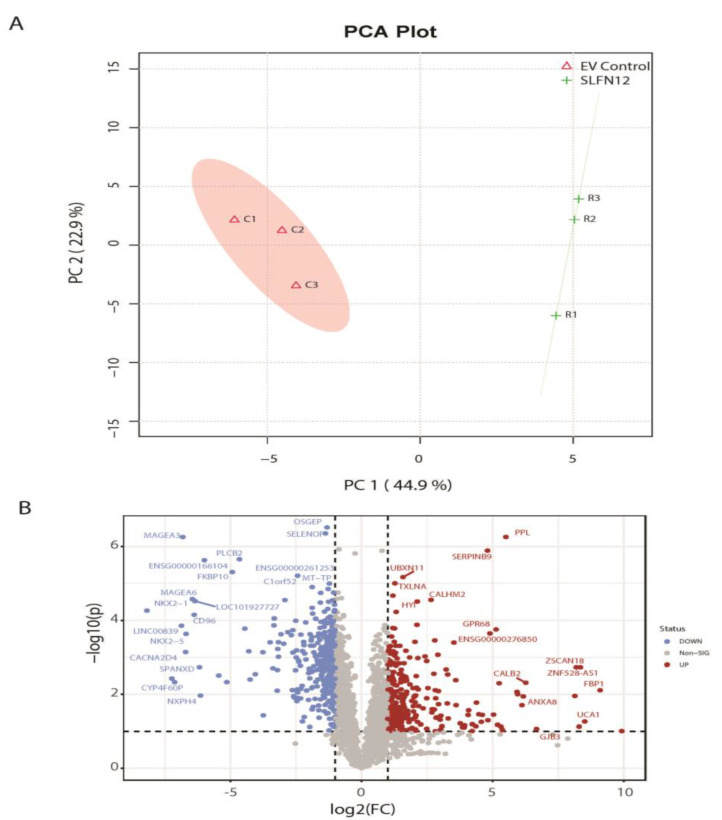
(**A**) Sample distribution of gene expression profiles: principal component analysis (PCA) plot showing clusters of samples based on similarity. The first two PCA components (PC1 and PC2) of the gene expression profile and overall variance between the groups are displayed. Each dot represents a sample color coded by both latency and SLFN12 expression. (**B**) Volcano plot of differentially expressed genes described in Supplementary Table S2.

A PCA analysis was performed together with EV control and LV-SLFN12 samples from patient-derived xenograft data to assess the similarities and differences in global gene expression patterns. There was a clear separation with 44.9% and 22.9% variation using the first and second principal components, respectively, between EV control and LV-SLFN12 (Figure 2A). The results suggest that there was a significant shift in the overall gene expression profile after the addition of SLFN12.

A differential gene expression analysis was performed to identify significant genes between EV control and LV-SLFN12 samples. *t*-tests identified 1182 human genes with significant *p*-values < 0.05 (Supplementary Table S2). To further define a list of significant genes, human genes with *p* < 0.05 and a fold change >2 were shortlisted. In addition, a few genes with a higher fold change of >4 (even with *p* > 0.05) were also included in this list to test the significance at the downstream level. The list was reduced to 549 significant genes (Supplementary Table S3, Figure 2B) with this selection criterion. Out of the 549 significant genes, 229 genes were upregulated, and 320 genes were downregulated in LV-SLFN12 compared to EV control, identifying important genes for further investigation. With a refined list of 549 significant genes that could be potentially related to SLFN12 expression, this list was then used to find the functional and pathway associations. 

### 3.3. Functional and Pathway Analysis of Significant Genes

#### 3.3.1. Functional Enrichment Analysis

**Figure 3:**
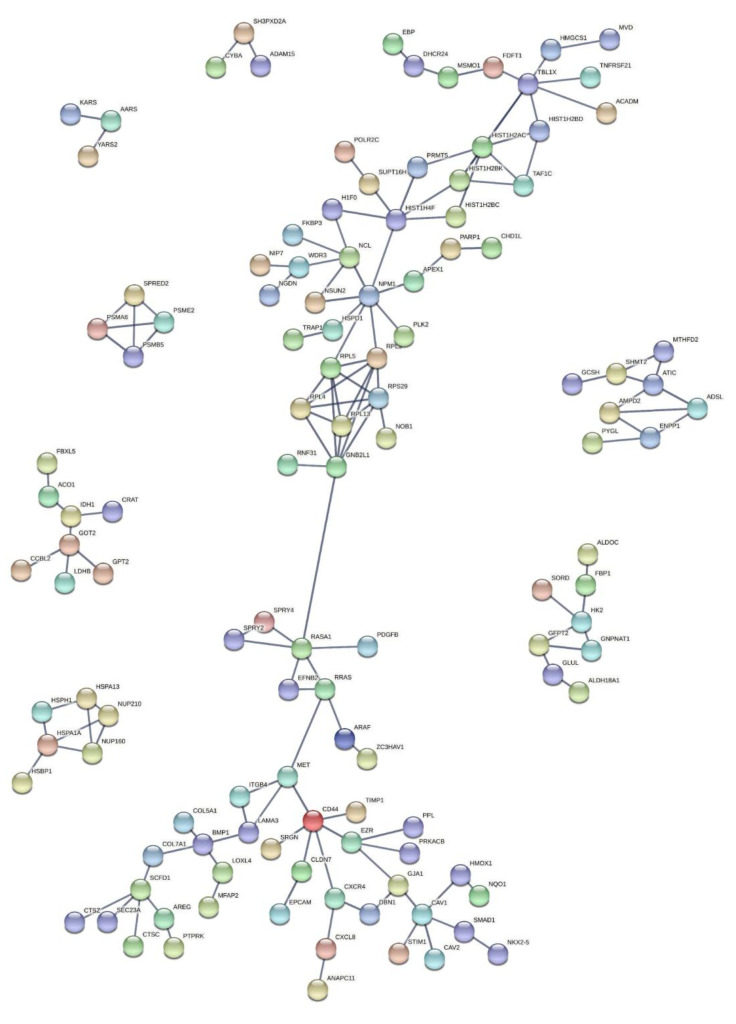
Functional enrichment analysis: STRING plot of interaction network functional enrichment analysis for all 512 genes based on the 549 genes from Supplementary Table S3. The connecting lines indicate the degree of interconnection and enrichment in certain molecular functions.

A functional enrichment analysis was performed using the STRING tool for the 549 differentially expressed genes as described above to identify known protein–protein interaction networks. Of the 549 genes, only 512 genes were used in the analysis, as 37 genes did not have a matching gene symbol, which is required by the STRING tool. The gene interaction network achieved a PPI enrichment *p*-value of 0.00714 (Figure 3), which indicated that the genes interacted more and were at least partially biologically connected as a group. Some of the notable genes central to the gene clusters and possibly connected to cancer in the interaction network included APEX1, NPM1, RPS29, MET, CD44, RRAS, CAV1, VEGFA, and ADAM15. This was able to indicate a possible connection to cancer. For instance, APEX1 overexpression is positively correlated with cancer progression, whereas NPM1 is known as a poor prognostic factor in breast cancer patients [32,33].

#### 3.3.2. Gene Set Enrichment Analysis

**Figure 4:**
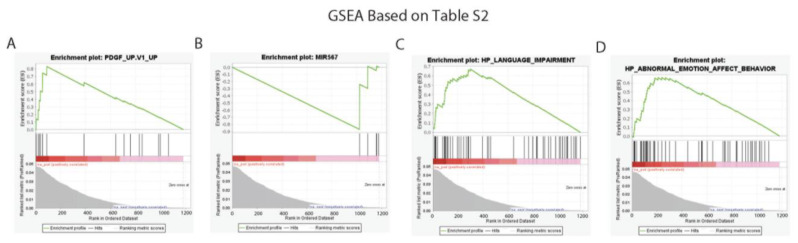
Enrichment plots from the gene set enrichment analysis: (**A**,**B**) are enrichment plots based on Supplementary Table S2’s 1182 genes with a minimum gene set size of five with FWER *p* < 0.05. (**C**,**D**) are enrichment plots based on Supplementary Table S2’s 1182 genes with a minimum gene set size of fifty with FWER *p* < 0.05.

First, a gene set enrichment analysis was performed to identify significant gene sets based on the results of the global gene expression analysis (Supplementary Table S2, 1182 human genes) to identify the enrichment scores of pathways with a nominal *p*-value < 0.05 for two different minimum gene set sizes of 5 and 50 (Supplementary Table S4, Supplementary Table S5, respectively) from molecular profile data. At the minimum gene set size of five, 915/931 pathways were upregulated and 16/931 were downregulated (Supplementary Tables S4A and S2). At the minimum gene set size of 50, 96 pathways were upregulated, and no downregulated pathways were identified (Supplementary Table S5). Pathways with an FWER *p*-value < 0.05 were identified and their enrichment plots were shown (Figure 4).

**Figure 5:**
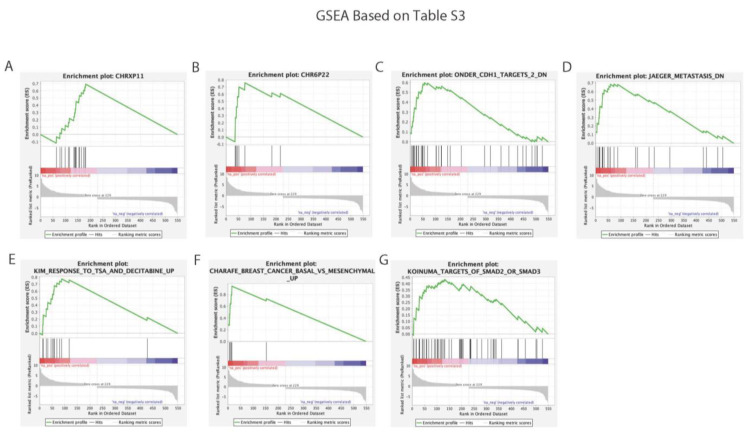
Enrichment plots from the gene set enrichment analysis. (**A**–**F**) are enrichment plots based on Supplementary Table S3’s 549 genes with a minimum gene set size of five with FWER *p* < 0.05. (**G**) is an enrichment plot based on Supplementary Table S3’s 549 genes with a minimum gene set size of fifty with FWER *p* < 0.05.

Then, a GSEA was performed again on the 549 significantly identified genes from Table S2 to find the associated pathways with a nominal *p*-value < 0.05 for two different minimum gene set sizes 5 and 50 (Supplementary Table S6, Supplementary Table S7, respectively). For a gene set minimum size of five, 458/647 pathways were upregulated and 189/647 were downregulated (Supplementary Table S6A,B). At the gene set minimum size of 50, 5/7 pathways were upregulated and 2/7 were downregulated (Supplementary Table S7). Pathways with an FWER *p*-value < 0.05 were identified with their enrichment plots (Figure 5). Both GSEAs verified important function associations based on the significant changes in gene expression after the introduction of SLFN12 and identified potential pathways that merited future investigation, including several pathways associated with breast cancer.

#### 3.3.3. Ingenuity Pathway Analysis

**Figure 6:**
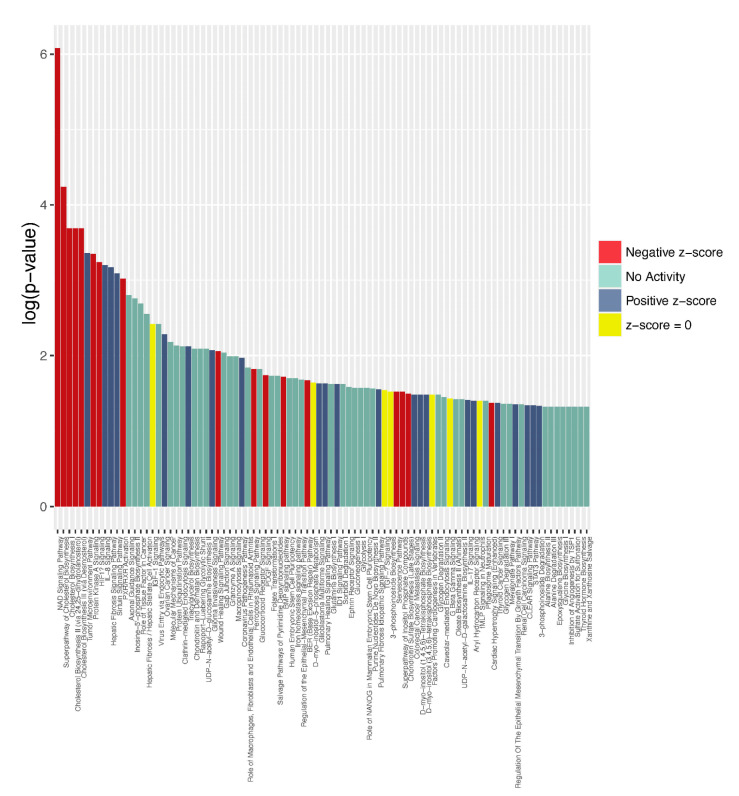
Ingenuity Pathway Analysis identified canonical pathways for human expression amongst −log(*p*-value) and z-score classes. The bar plot describes all the canonical pathways for the z-score positive, negative, zero, and no activity pattern.

To begin to investigate the molecular mechanisms potentially related to SLFN12, information on differentially expressed genes defined in Supplementary Table S3 was classified into related canonical pathways based on the IPKB (Ingenuity Pathway Knowledge Base). The top enriched categories of canonical pathways with a −log(*p*-value) and z-score class are listed in Figure 6 for each pathway. The z-score describes the activation or inhibition of genes. The pathways are sorted in the order of their significance with a threshold *p*-value of 0.05, corresponding to a −log(*p*-value) of 1.3. This analysis identified the NAD signaling pathway as the most statistically significant with a negative z-score. The superpathway of the cholesterol biosynthesis and three additional cholesterol biosynthesis pathways were next in significance with negative z-score values. The nicotinamide adenine dinucleotide (NAD) signaling pathway was ranked first in this list. NAD is a coenzyme that mediates redox reactions in diverse metabolic pathways, and increased levels of NAD enhance glycolysis and fuel cancer cells [34]. Next, were several pathways associated with the cholesterol biosynthesis that play a complex role in supporting cancer progression and suppressing immune responses [35].

### 3.4. Single-Cell Analysis for Patient-Derived Xenografts

**Figure 7:**
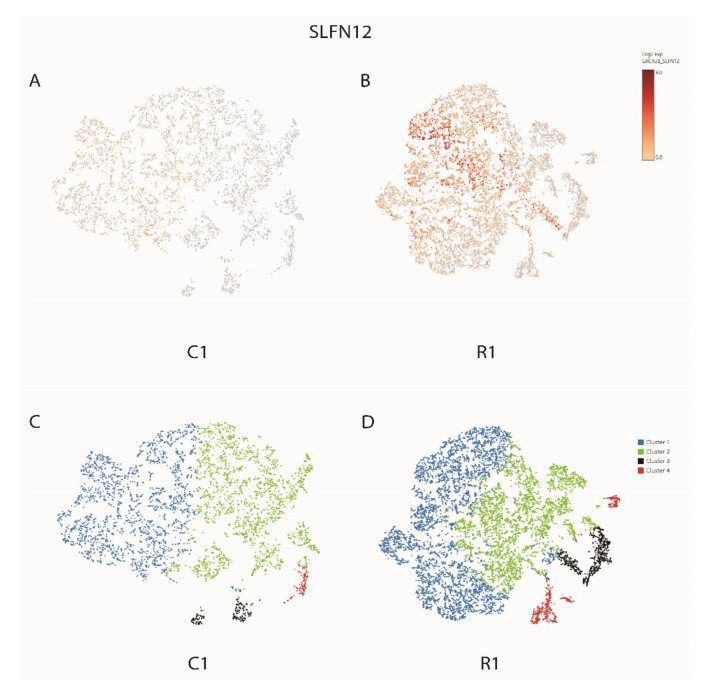
Single-cell expression of SLFN12 in (**A**) C1 and (**B**) R1. Log2 expression in 0–9.0. (**C**) Four K-means clusters of C1. (**D**) Four K-means clusters of R1.

Based on single-cell sequencing technology, differences between xenograft conditions were investigated. Using this approach, two samples, i.e., EV-control (C1: included a normal level of SLFN12 expression) and LV-SLFN12 (R1: was modified with a lentivirus to overexpress SLFN12) were examined. Figure 7 shows an overall SLFN12-gene expression only in human MDA-MB-231 cells of the C1 and R1 xenografts. We found very limited expression of SLFN12 in C1 whereas it was significantly higher in R1 (Figure 7B) with a greater number of cells. 

Four K-means clusters were selected as described in the methods to further evaluate the functional properties of cells across those two conditions (i.e., C1 vs. R1) (Figure 7C,D). A review of the *p*-values of all genes in the analysis showed that the fourth cluster had no genes with *p* < 0.05 in C1 and only had 25 significant genes in R1 (Table 1: summary table, Supplementary Table S8: complete gene list). 

**Figure 8:**
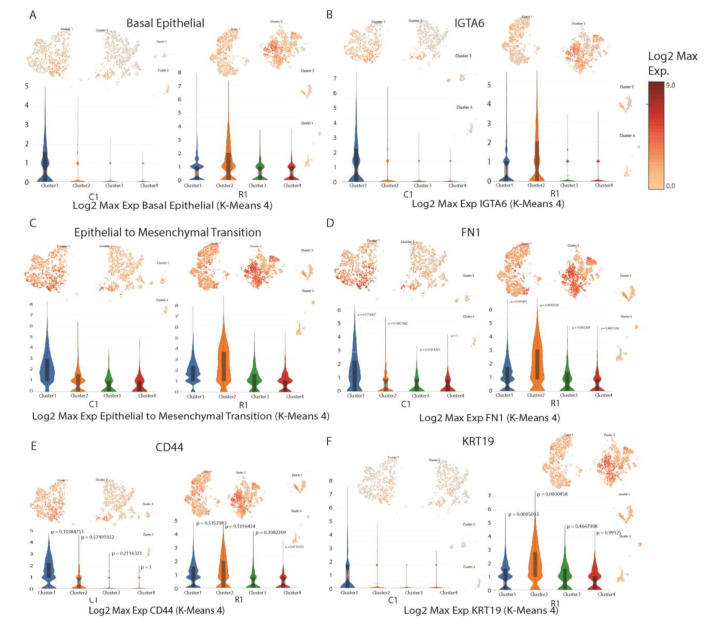
Cell type from single-cell analysis. (**A**) Basal epithelial cell type’s overall expression (**B**) IGTA6 gene expression (basal epithelial cell type gene) (**C**) Epithelial-to-mesenchymal transition cell type’s overall expression (**D**) FN1 gene expression (epithelial-to-mesenchymal transition cell type gene) (**E**) CD44 gene expression (epithelial-to-mesenchymal transition cell type gene). (**F**) KRT19 gene expression (luminal epithelial cell type gene). Log2 expression in 0–9.0. *p*-values given for significant clusters identified by the single-cell analysis.

The single-cell analysis showed an overall greater expression in R1 in the identified basal epithelial cell type compared with C1 (Figure 8A). The second cluster in R1 showed a higher expression level than other clusters in both R1 and C1. Within the basal epithelial cell type, there was a greater gene expression of KRT14 and IGTA6 and the greater expression was prominently shown in the second cluster (Supplementary Figure S2A, Figure 8B). Within the epithelial-to-mesenchymal transition cell type, there was an overall greater expression in R1 compared with C1, and in genes FN1 and CD44, where the second cluster in R1 showed a higher expression than in C1 (Figure 8C–E). For the epithelial cell type, the single-cell analysis showed that the overall expression was stronger in R1’s second cluster as compared to C1’s second cluster (Supplementary Figure S2B). Within the epithelial cell type, EPCAM, EGFR, and ERBB2 had differing expressions between C1 and R1. In EPCAM, R1’s overall expression was greater than C1’s (Supplementary Figure S2C). In EGFR, C1’s first cluster had a relatively large expression when compared to other clusters within EGFR (Supplementary Figure S2E). Within ERBB2 expression, relatively little expression was detected in C1’s clusters with greater expression in R1’s clusters (Supplementary Figure S2D). In the luminal epithelial cell type, overall expression was higher in R1 when compared to C1 (Supplementary Figure S2F). MUC1, KRT8, KRT18, and KRT19 had differing expressions between C1 and R1 within this cell type. MUC1 expression was overall greater in R1 than C1 (Supplementary Figure S2G). KRT8, KRT18, and KRT19 had overall greater expression in R1 and much of this expression was concentrated in R1’s second cluster (Supplementary Figure S2H,I, Figure 8F). Keratin 8 (KRT8) plays an essential role in the development and metastasis of several human cancers [36,37]. For instance, KRT18 induces an epithelial–mesenchymal transition (EMT) in human breast cancer [38] and KRT19 plays a critical role in breast cancer proliferation [39].

### 3.5. Favorable Survival with High Expression of SLFN12

Kaplan–Meier curves were generated to estimate the survival function from four publicly available datasets (Supplementary Table S1). The patients were classified into low and high SLFN12 status using machine learning based an optimal cutoff and median cutoff. SLFN12 and all four gene signatures were evaluated independently.

**Figure 9:**
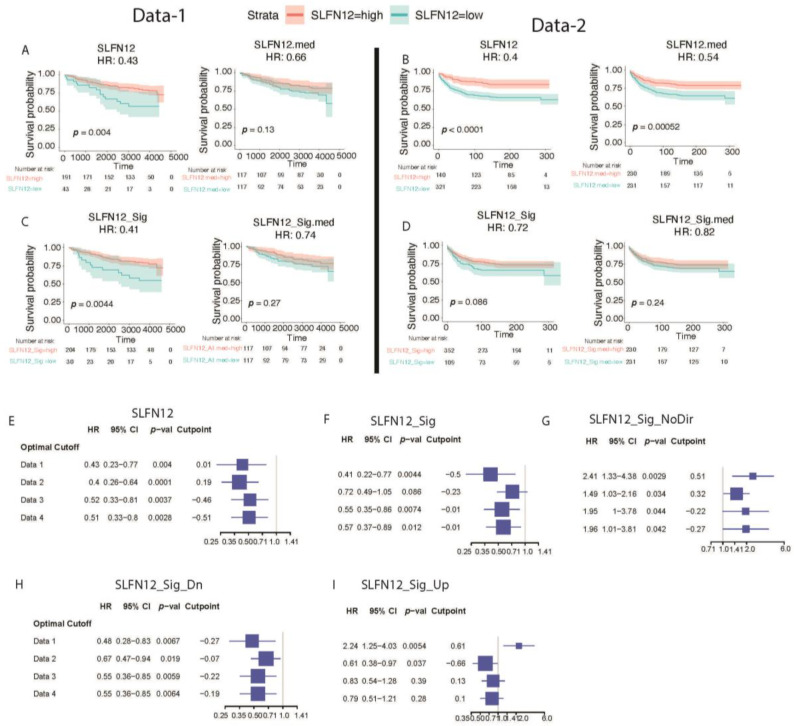
Kaplan–Meier curve describing the relapse-free survival (RFS) association of SLFN12, SLFN12 signature, and SLFN12 downregulated gene sets with the survival probability based on (**A**) optimal cutoff and (**C**) median score for Data-1. Kaplan–Meier curve describing the distant metastasis-free survival (DMFS) association of SLFN12 and SLFN12 signature with the survival probability based on (**B**) optimal cutoff and (**D**) median score for Data-2. Hazards Ratios (HRs) based on optimal cutoffs of Data-1, Data-2, Data-3, and Data-4 for signatures: (**E**) SLFN12, (**F**) SLFN12 Sig, (**G**) SLFN12 genes without direction, (**H**) SLFN12 downregulated genes, (**I**) SLFN12 upregulated genes as defined in the methods. Horizontal bars represent the 95% CIs of the HRs.

There was a significant difference in terms of hazard ratios with 0.43 (*p* = 0.004) and 0.66 (*p* = 0.13) for the optimal cutoff and median score, respectively, for the SLFN12 gene alone using Data-1 (Figure 9A). When the SLFN12 gene signature, i.e., SLFN12_Sig was examined, the hazard ratios were 0.41 (*p* = 0.0044) and 0.74 (*p* = 0.27) for the optimal cutoff and median score, respectively (Figure 9C). Similar results were illustrated for the SLFN12 upregulated (SLFN12_Up), SLFN12 downregulated (SLFN12_Dn), and SLFN12 signature genes without using the direction of the genes (SLFN12_Sig_NoDir) for the optimal cutoff and median score using relapse-free survival (RFS) (Figure 9A,C). Similar plots for Data-2 are demonstrated in Figure 9B,D.

To test the reproducibility power of the outcome, we used a total of four different datasets and a forest plot was created using the HR, 95% confidence interval, and *p*-value for the SLFN12 gene alone and all four gene signatures (Figure 9E–I). The results were very consistent across all the four different datasets using both optimal and median cutoffs (Figure 9E–I, Supplementary Figure S3). The distributions, maximally selected rank statistics, and Kaplan–Meier plots for all signature conditions are shown and provided in the following supplements (Supplementary Figures S4–S7). The survival curves suggest that a low SLFN12 expression in the tumor is associated with a poor prognosis for SLFN12 alone as well as for all of the gene signatures identified, with better survival prediction capabilities for the SLFN12 gene signature than for SLFN12 alone.

#### Conformation qPCR Analysis of SLFN12 Signature Genes

**Figure 10:**
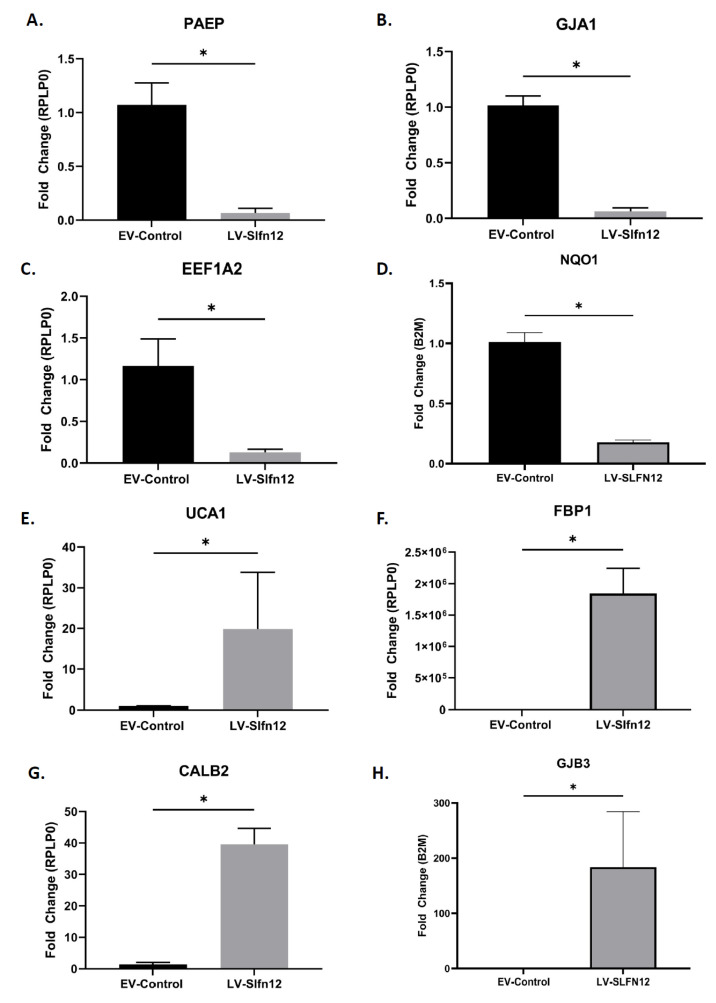
Conformation qPCR analysis of SLFN12 signature genes for EV-control and LV-SLFN12 including: (**A**) PAEP, (**B**) GJA1, (**C**) EEF1A2, (**D**) NQO1, (**E**) UCA1, (**F**) FBP1, (**G**) CALB2, and (**H**) GJB3. RPLP0 or B2M was used as a reference gene (n = 4–5, * *p* < 0.01). Data are expressed as mean ± SEM.

Utilizing qPCR, we measured the RNA expression of eight genes from the SLFN12 signature gene set (Supplementary Table S3) that were shown to be significantly up- or downregulated by RNA-seq and are known to be involved in breast cancer progression or regulation. PAEP, GJA1, EEF1A2, and NQO1 were confirmed by qPCR to be downregulated in the LV-SLFN12 samples compared to the EV-Control and UCA1, FBP1, CALB2, and GJB3 were confirmed to be upregulated (Figure 10).

### 3.6. SLFN12 Expression Behaves Differently amongst Different Races

**Figure 11:**
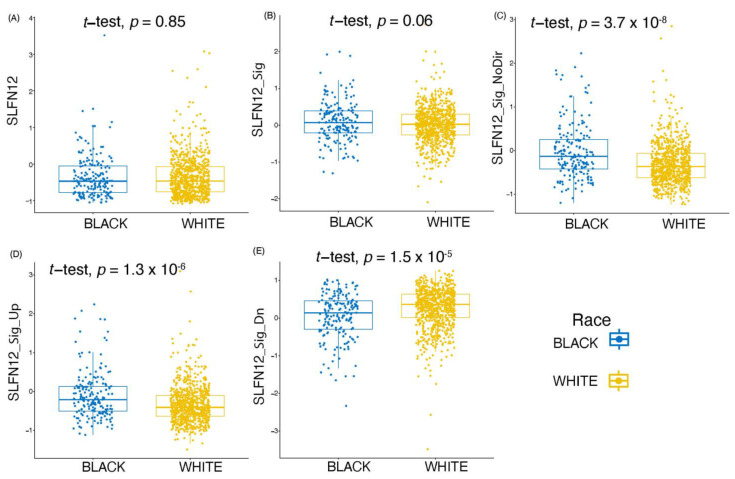
A box plot describing the race difference for (**A**) SLFN12, (**B**) SLFN12_Sig, (**C**) SLFN12_Sig_NoDir, (**D**) SLFN12_Sig_Up, and (**E**) SLFN12_Sig_Dn genes for white and black patients for Data-1.

To investigate potential racial disparity in SLFN12 biology across European (white) and African (black) populations, we analyzed the gene expression pattern of SLFN12 alone, and SLFN12 signature genes (SLFN12_Sig) in two different datasets. We further analyzed three other signatures (SLFN12_Sig_Up, SLFN12_Sig_Dn, and SLFN12_Sig_NoDir) to test their power of detection of racial differences. For Data-1 (Figure 11), significant racial differences were found for SLFN12_Sig_NoDir, SLFN12_Up, and SLFN12_Dn gene signatures (Figure 11C–E), while Data-2 (Supplementary Figure S8) showed a significant difference between black and white patients for only the SLFN12_Sig gene signature (Supplementary Figure S8B). The significant difference captured between races (such as African American and Caucasian American patients in this case) suggests the potential importance of SLFN12 in precision medicine.

## 4. Discussion

Our initial in vitro work had demonstrated that exogenous SLFN12 expression in TNBC cells slowed proliferation and reduced invasion [2]. Our present results now demonstrate that SLFN12 expression in vivo reduces tumorigenesis, increases tumor latency, and leads to a significantly smaller tumor volume. From these overexpressed SLFN12 xenograft tumors, an RNA-Seq analysis was performed and in the global gene expression analysis, we were able to identify major genes and functional pathways. Our xenografts were established from the triple-negative MDA-MB-231 cells which are characterized by a low expression of the luminal markers (KRT8, MUC1, EPCAM) [40,41]. However, the single-cell analysis showed that xenografts established from SLFN12-overexpressing MDA-MB-231 cells had a higher expression of luminal and epithelial markers. SLFN12 induces differentiation in enterocytes and breast cancer cells in 2D cultures [2,4]. Our current results show that SLFN12-overexpressing xenografts have a higher expression of luminal markers (KRT8, MUC1, and EPCAM) indicating that SLFN12 promoted TNBC cells from the undifferentiated basal phenotype toward a more differentiated luminal phenotype which is less aggressive and responds better to chemotherapy [40,41,42]. This could explain the positive correlation of SLFN12 expression with better survival in TNBC. Moreover, it could provide a differentiation therapy approach to target TNBC through modulating SLFN12 expression. Whether the switch from basal to luminal is partial or complete needs to be investigated in a dedicated study.

TNBC lacks the expression of estrogen, progesterone, and HER2 receptors [43]. Interestingly, our single-cell analysis showed an increased expression of both ERBB2 and EGFR in SLFN12-overexpressing xenografts. ERBB2/HER2 is a marker that is not expressed in TNBC [44], and HER2+ breast cancers respond well to trastuzumab (anti-HER2) [44,45]. Therefore, TNBC patients with a high SLFN12 expression might respond better to trastuzumab therapy. Similarly, higher SLFN12-expressing TNBC might respond better to EGFR inhibitors. EGFR inhibitors showed a limited response in TNBC clinical trials with variable response rates [46,47]. Therefore, it may be worthwhile in the future to explore selecting candidates for EGFR inhibitor therapeutic trials based on the expression of specific markers, such as SLFN12, that might improve the response and outcome of EGFR inhibitors in TNBC.

Although the basal markers (KRT14, IGTA6) and EMT markers (FN1, CD44) expressions were increased in SLFN12-overexpressing xenograft, the increase might be a compensatory mechanism to restore the basal phenotype of the cells and counteract the differentiating effect of SLFN12. Nevertheless, our tumor growth and survival data indicated that such increases did not abolish the effect of SLFN12.

The top two canonical pathways based on IPKB were the NAD signaling pathway and the superpathway of the cholesterol biosynthesis. Each had a negative z-score value, indicating that the overexpression of SLFN12 was downregulating these pathways. The NAD pathway has been implicated in the progression of multiple metabolic disorders, cancers, and neurodegenerative diseases [48]. NAD is an important factor in early tumorigenesis and can become a harmful factor in the cancer phase and promotion phase. It can either prevent or reverse the phenotype of malignant cells at early cancer stages [49]. Alterations in cholesterol pathways could also regulate mechanisms that are involved in the development of breast cancer. Very low density lipoproteins (VLDL) and LDL are necessary for breast cancer proliferation, anchorage-independent growth, EMT development, angiogenesis, and metastasis [50]. Conversely, high-density lipoproteins (HDL) reduces mammosphere formation and the mammary tumoral cells have augmented radiation sensitivity [50]. HDL levels have also been inversely correlated to breast cancer risk based on race. African American and Taiwanese women have low HDL levels, which was then connected to an elevated risk of breast cancer [50]. 

Our analysis demonstrates that the SLFN12 gene signature predicts a better survival in breast cancer. The reportability and robustness of the results across different datasets support the validity of this outcome. Although SLFN12 expression was similar in tumors from African Americans (black) and Caucasian Americans (white), we found a significant difference in the SLFN12 gene signature (Supplementary Table S3) between the two races. Such differences remained valid whether we analyzed the data in the SLFN12_Sig_NoDir, SLFN12_Sig_Up, or SLFN12_Sig_Dn gene signature genes (Figure 11C–E). Therefore, these data could indicate that if we had a targeted therapy that increased the expression of SLFN12, then the SLFN12_Upreg signature genes would be further upregulated and the SLFN12_Dnreg signature genes would be further downregulated in the African American population, which could lead to a better breast cancer survival rate. 

In a recent cohort study, African American women had nearly a threefold increased risk of triple-negative breast cancers and African American women with TNBC had a worse prognosis than Caucasian women [51]. Differences in socioeconomic status and the difficult access of African Americans to better health care and state-of-the-art health procedures surely contribute to such disparities in outcome [52]. However, differences in biology between different races might also affect African American breast cancer outcomes [53,54]. Differences in expression of SLFN12 gene signatures might be one of these biological differences. 

We have previously reported that SLFN12 itself is predictive of survival in triple-negative breast cancer but not in other breast cancers [2]. However, the present results indicate that the SLFN12-associated gene signature appears to predict survival not only in TNBC but in all breast cancers and is more predictive than SLFN12 alone. Furthermore, this SLFN12 gene signature could serve as a prognostic predictor of breast cancer severity and could help to develop personalized targeted therapy for African Americans or patients of other races. 

## 5. Conclusions

Schlafen 12 can induce differentiation and predict survival in triple negative breast cancer patients, as well as other types of breast cancer. SLFN12 signature genes could be part of the network that explain severity of triple negative breast cancer in African American and might play a role in future design of personalized therapy for patients with breast cancer.

## Figures and Tables

**Table 1:** Genes with *p* < 0.05 based on a single-cell K-means analysis. Genes for C1 and R1 are shown in parentheses for each cluster with overlapping genes shown in the table.

		C1			
		Cluster 1 (236)	Cluster 2 (271)	Cluster 3 (91)	Cluster 4 (0)
**R1**	Cluster 1 (200)	87	112	41	0
	Cluster 2 (225)	99	126	40	0
	Cluster 3 (15)	4	4	20	0
	Cluster 4 (25)	6	19	19	0

## Data Availability

The data generated in this study are publicly available in Gene Expression Omnibus (GEO) at GSE215442.

## Supplementary Materials

The following supporting information can be downloaded at: https://www.mdpi.com/article/10.3390/cancers15020402/s1, Figure S1: Flowchart of the study design; Figure S2: Cell type single cell analysis; Figure S3: hazards ratios (HRs) based on median cutoffs of data-1, data-2, data-3, and data-4 for signatures; Figure S4: Distribution, Maximally Selected Rank Statistics, and Kaplan Meier survival plots for Data-1; Figure S5: Distribution, Maximally Selected Rank Statistics, and Kaplan Meier survival plots for Data-2; Figure S6: Distribution, Maximally Selected Rank Statistics, and Kaplan Meier survival plots for Data-3; Figure S7: Distribution, Maximally Selected Rank Statistics, and Kaplan Meier survival plots for Data-4; Figure S8: A box plot describing the race difference; Table S1: Dataset characteristics; Table S2: Global gene expression analysis of genes with *p* < 0.05; Table S3: Global Gene Expression Analysis of Human Genes with *p* < 0.05 and FC > 2 and FC < 0.5, and genes with FC > 4-fold; Table S4: GSEA identified gene sets with minimum size 5 and nominal *p*-value < 0.05 based on Table 1’s 1182 genes (*p* < 0.05); Table S5: GSEA identified gene sets with minimum size 50 and nominal *p*-value < 0.05 based on Table 1’s 1182 genes (*p* < 0.05); Table S6: GSEA identified gene sets with minimum size 5 and nominal *p*-value < 0.05 based on Table S2’s 549 genes; Table S7: GSEA identified gene sets with minimum size 50 and nominal *p*-value < 0.05 based on Table S2’s 549 genes; Table S8: *p*-values from Single Cell analysis of C1 and R1.
