# Supplementary material for: Schlafen 12 Slows TNBC Tumor Growth, Induces Luminal Markers, and Predicts Favorable Survival
Source: Cancers (Basel). 2023 Jan 7;15(2):402. doi: 10.3390/cancers15020402 (PMC9856841; doi:10.3390/cancers15020402)
Supplement: Supplementary file 1 [file cancers-15-00402-s001.zip › Supplemental Figure 4 (1).pdf]

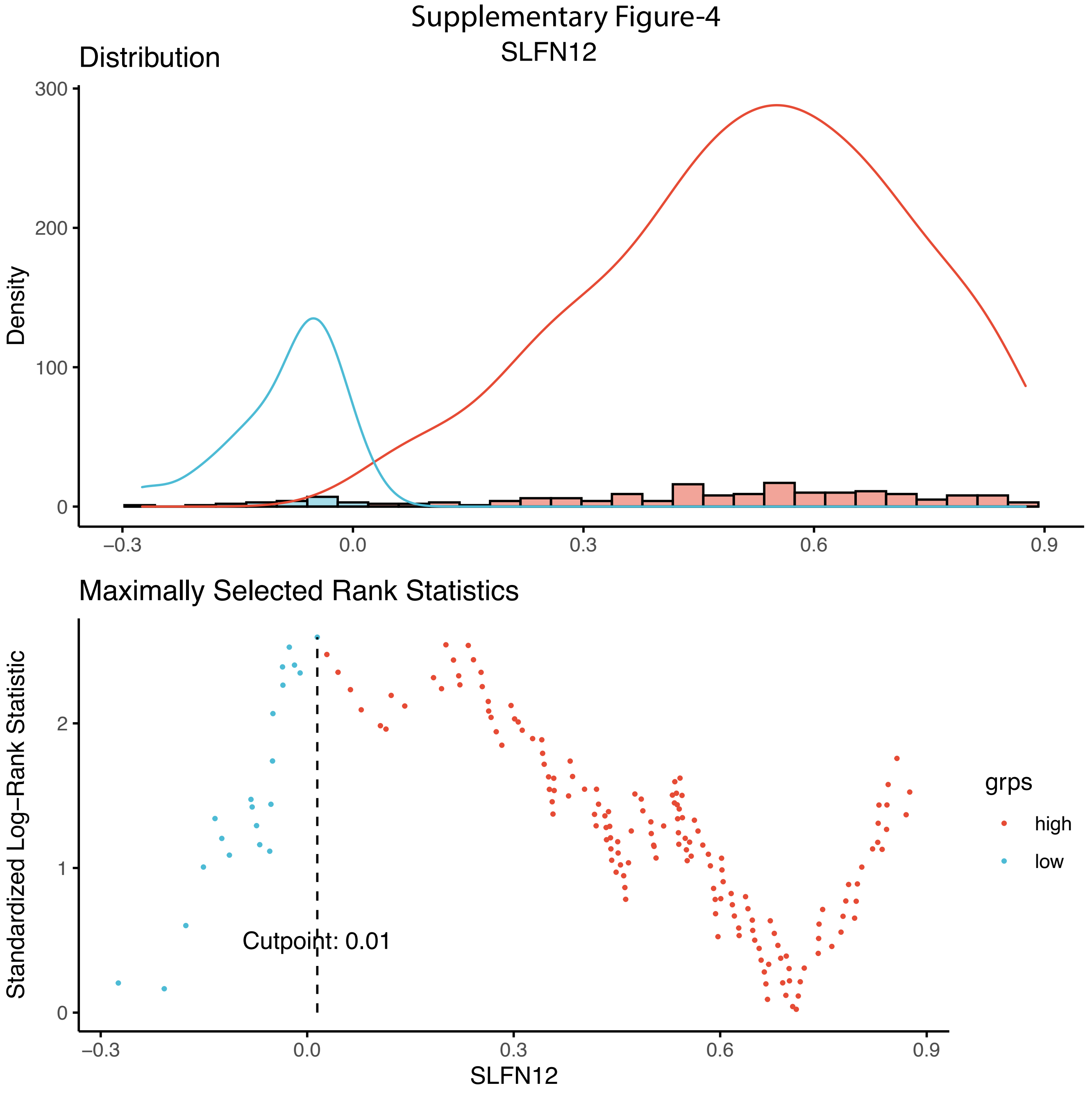
Supplementary Figure-4
SLFN12
Distribution
Density
300
200
100
0
−0.3
0.0
0.3
0.6
0.9
Maximally Selected Rank Statistics
Standardized Log−Rank Statistic
2
1
0
Cutpoint: 0.01
−0.3
0.0
0.3
0.6
0.9
SLFN12
grps
high
low

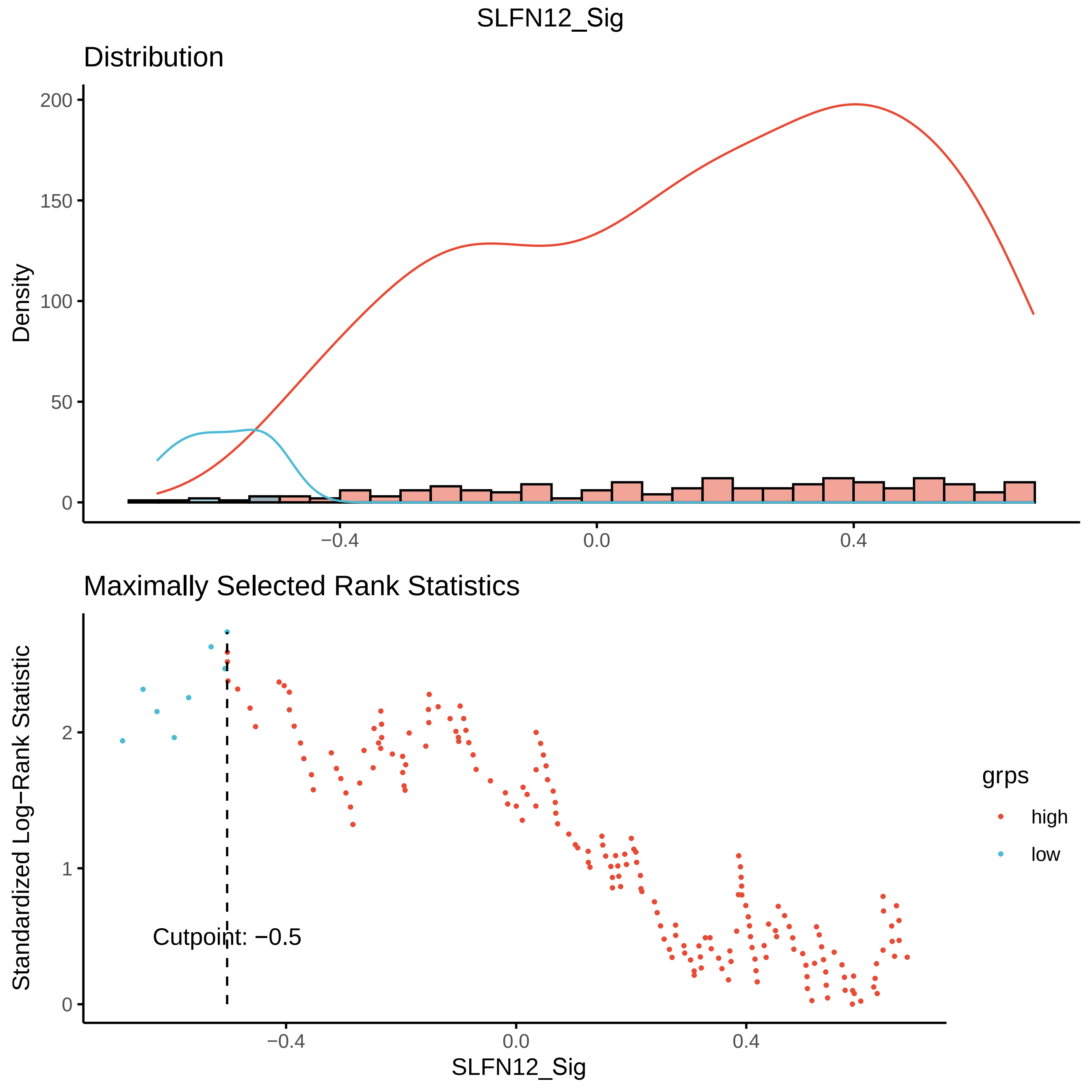

SLFN12_Sig
Distribution
Density
200
150
100
50
0
−0.4
0.0
0.4
Maximally Selected Rank Statistics
Standardized Log−Rank Statistic
2
1
0
Cutpoint: −0.5
−0.4
0.0
0.4
SLFN12_Sig
grps
high
low

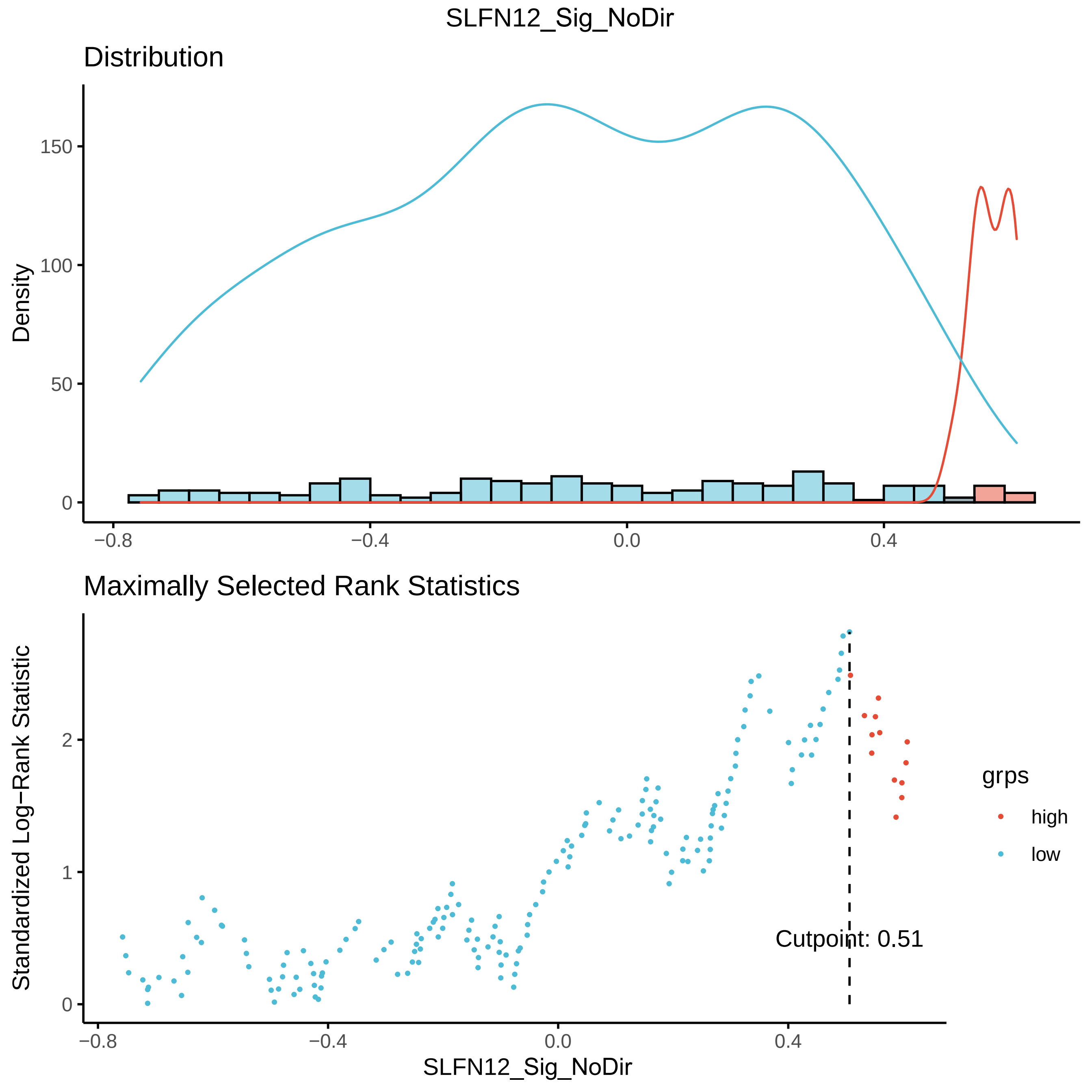

SLFN12_Sig_NoDir
Distribution
Density
150
100
50
0
−0.8
−0.4
0.0
0.4
Maximally Selected Rank Statistics
Standardized Log−Rank Statistic
2
1
0
Cutpoint: 0.51
−0.8
−0.4
0.0
0.4
SLFN12_Sig_NoDir
grps
high
low

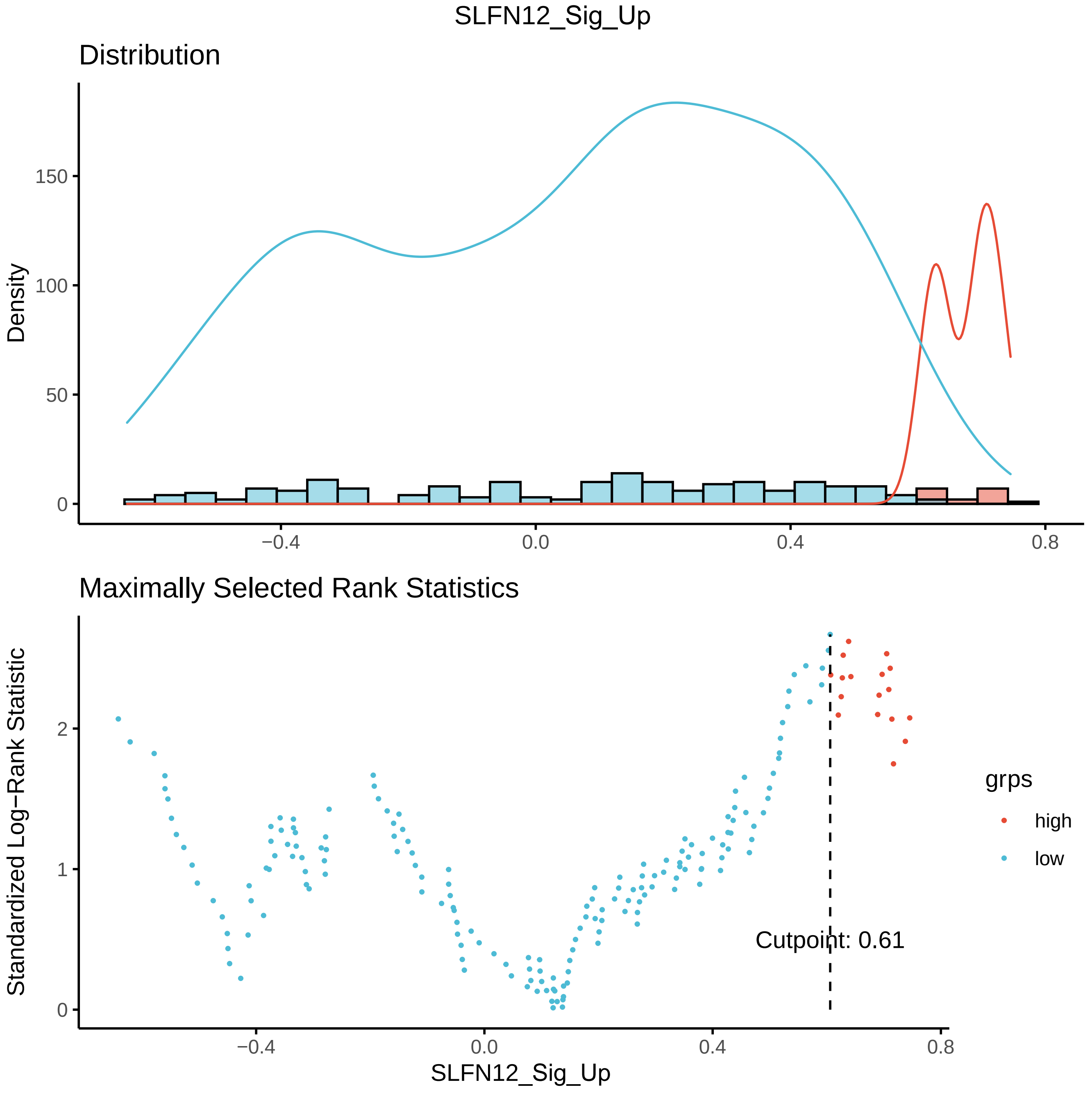

SLFN12_Sig_Up
Distribution
Density
150
100
50
0
−0.4
0.0
0.4
0.8
Maximally Selected Rank Statistics
Standardized Log−Rank Statistic
2
1
0
Cutpoint: 0.61
grps
high
low
SLFN12_Sig_Up

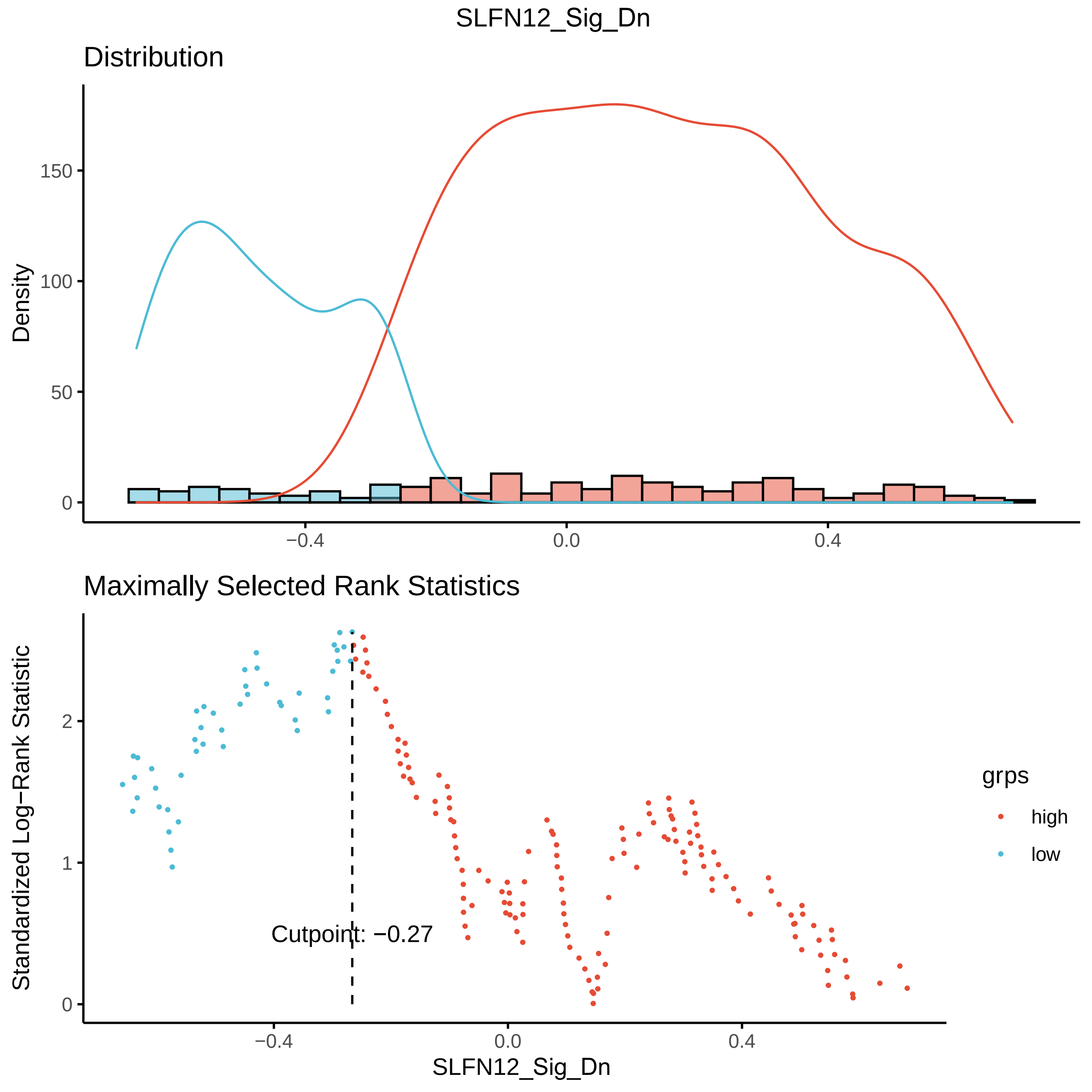

SLFN12_Sig_Dn
Distribution
Density
150
100
50
0
−0.4
0.0
0.4
Maximally Selected Rank Statistics
Standardized Log−Rank Statistic
2
1
0
Cutpoint: −0.27
−0.4
0.0
0.4
SLFN12_Sig_Dn
grps
high
low

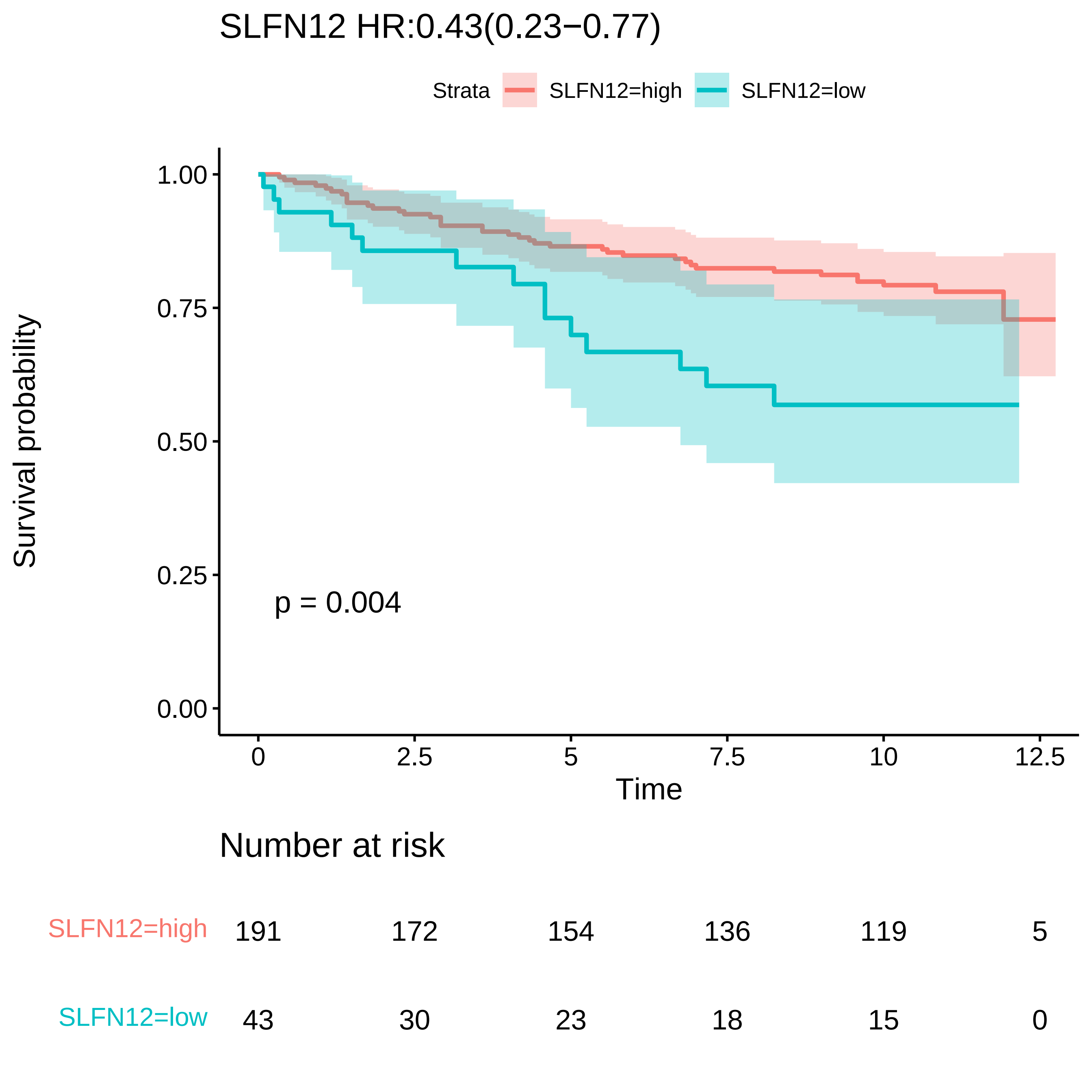

SLFN12 HR:0.43(0.23−0.77)
Strata
SLFN12=high
SLFN12=low
Survival probability
1.00
0.75
0.50
0.25
0.00
p = 0.004
0
2.5
5
7.5
10
12.5
Time
Number at risk
SLFN12=high
191
172
154
136
119
5
SLFN12=low
43
30
23
18
15
0

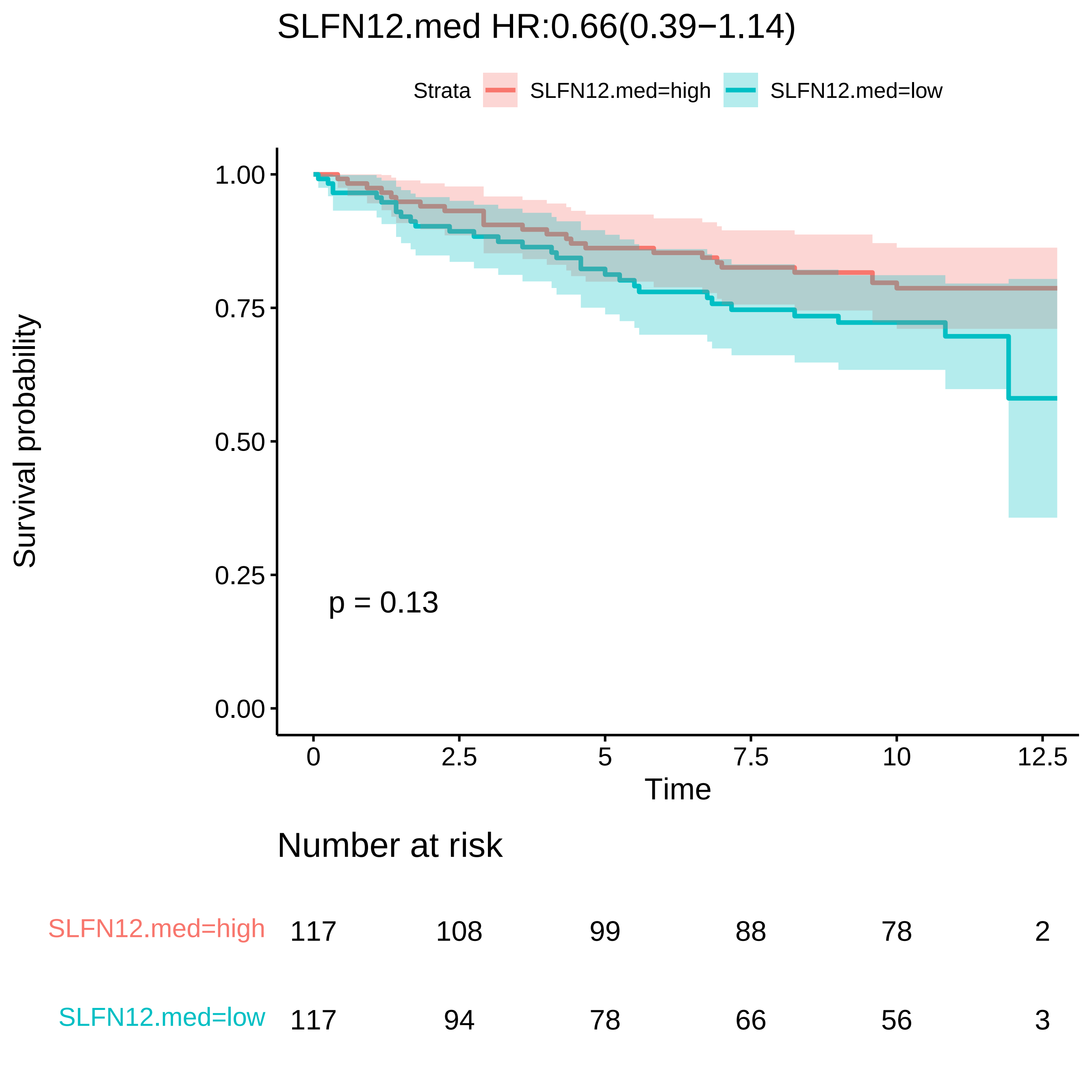
SLFN12.med HR:0.66(0.39−1.14)
Strata
SLFN12.med=high
SLFN12.med=low
Survival probability
1.00
0.75
0.50
0.25
0.00
p = 0.13
0
2.5
5
7.5
10
12.5
Time
Number at risk
SLFN12.med=high 117 108 99 88 78 2
SLFN12.med=low 117 94 78 66 56 3

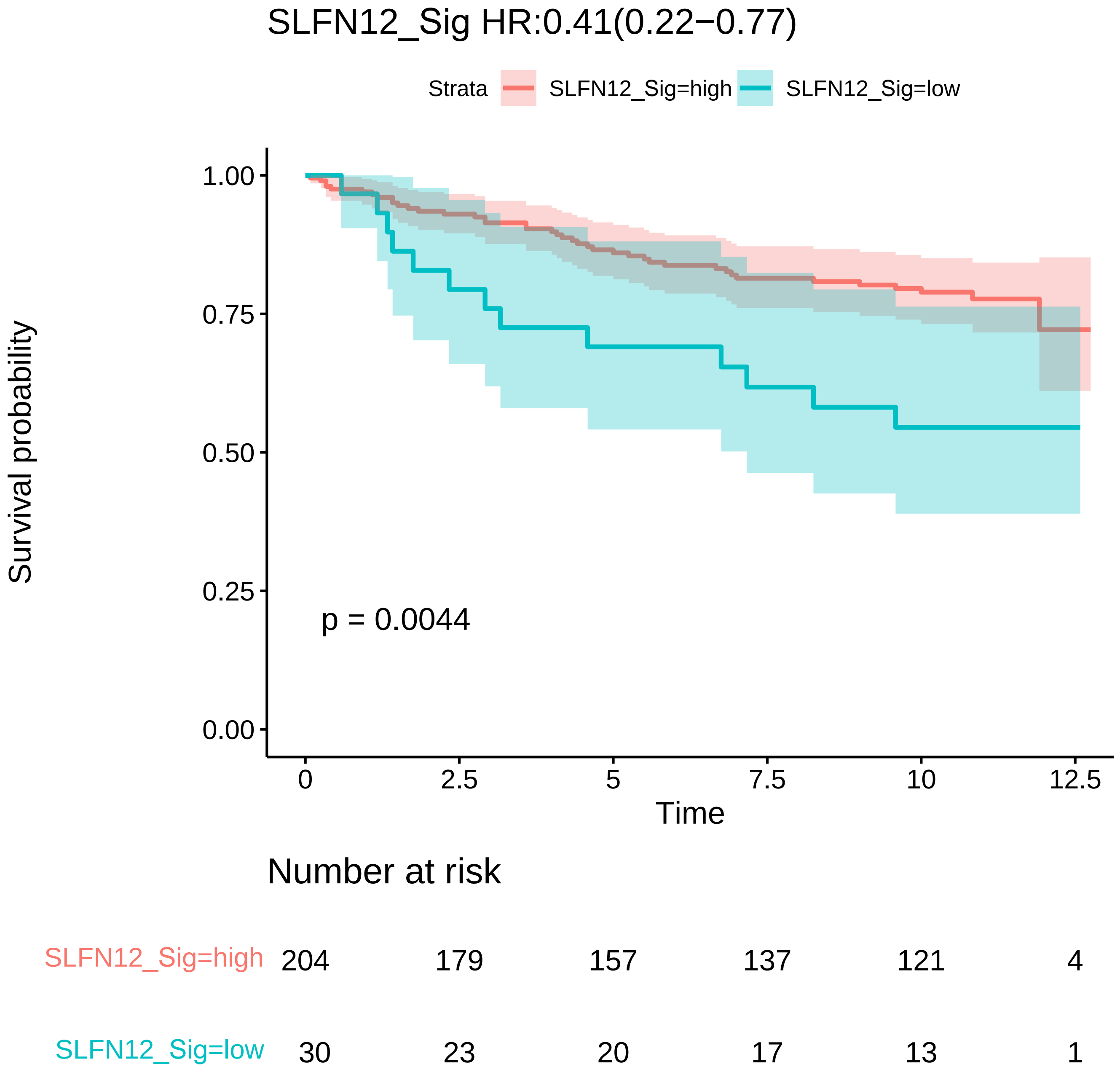
SLFN12_Sig HR:0.41(0.22−0.77)
Strata
SLFN12_Sig=high
SLFN12_Sig=low
Survival probability
1.00
0.75
0.50
0.25
0.00
p = 0.0044
0
2.5
5
7.5
10
12.5
Time
Number at risk
SLFN12_Sig=high 204 179 157 137 121 4
SLFN12_Sig=low 30 23 20 17 13 1

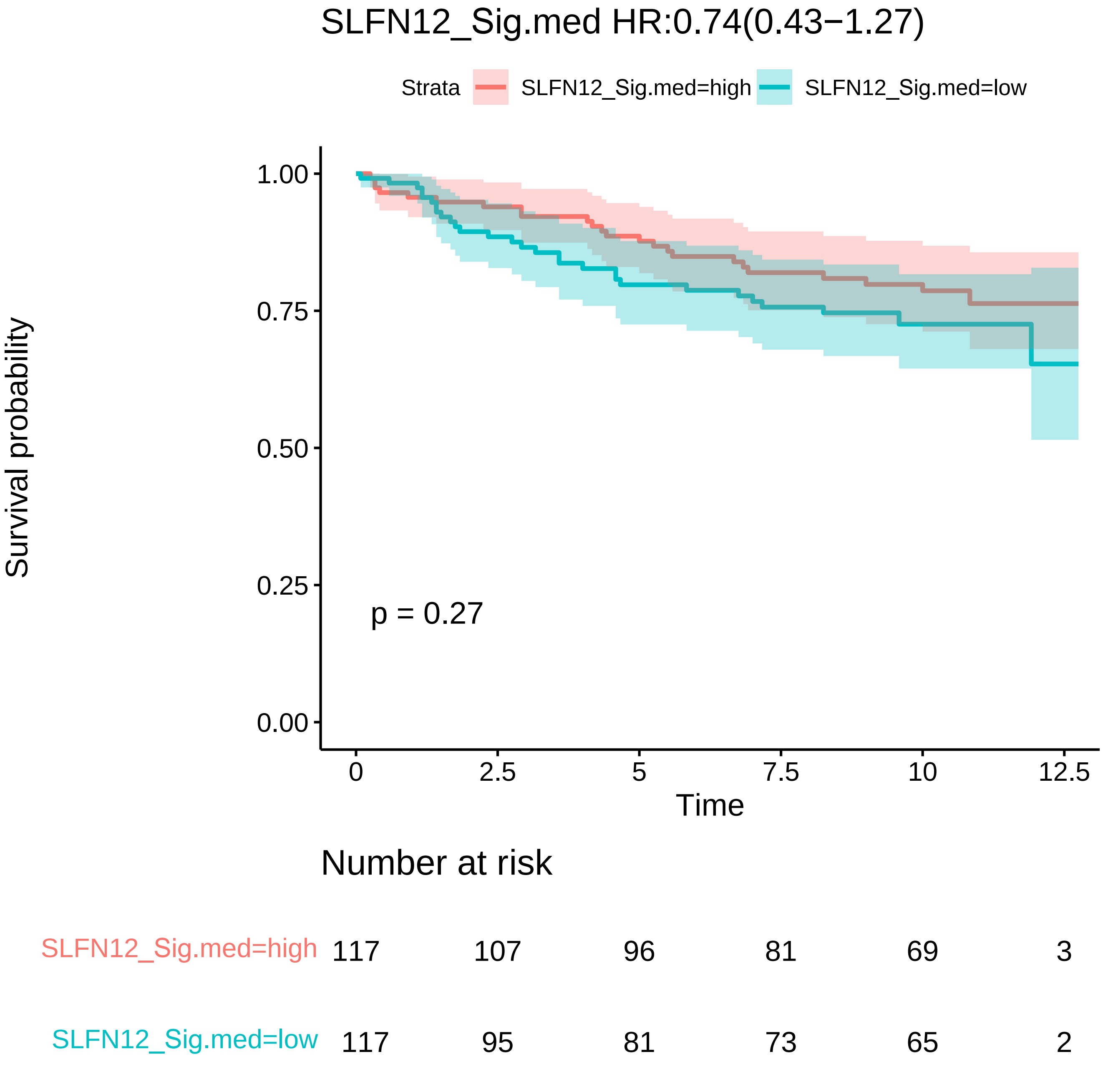
SLFN12_Sig.med HR:0.74(0.43−1.27)
Strata
SLFN12_Sig.med=high
SLFN12_Sig.med=low
Survival probability
1.00
0.75
0.50
0.25
0.00
p = 0.27
0
2.5
5
7.5
10
12.5
Time
Number at risk
SLFN12_Sig.med=high 117 107 96 81 69 3
SLFN12_Sig.med=low 117 95 81 73 65 2

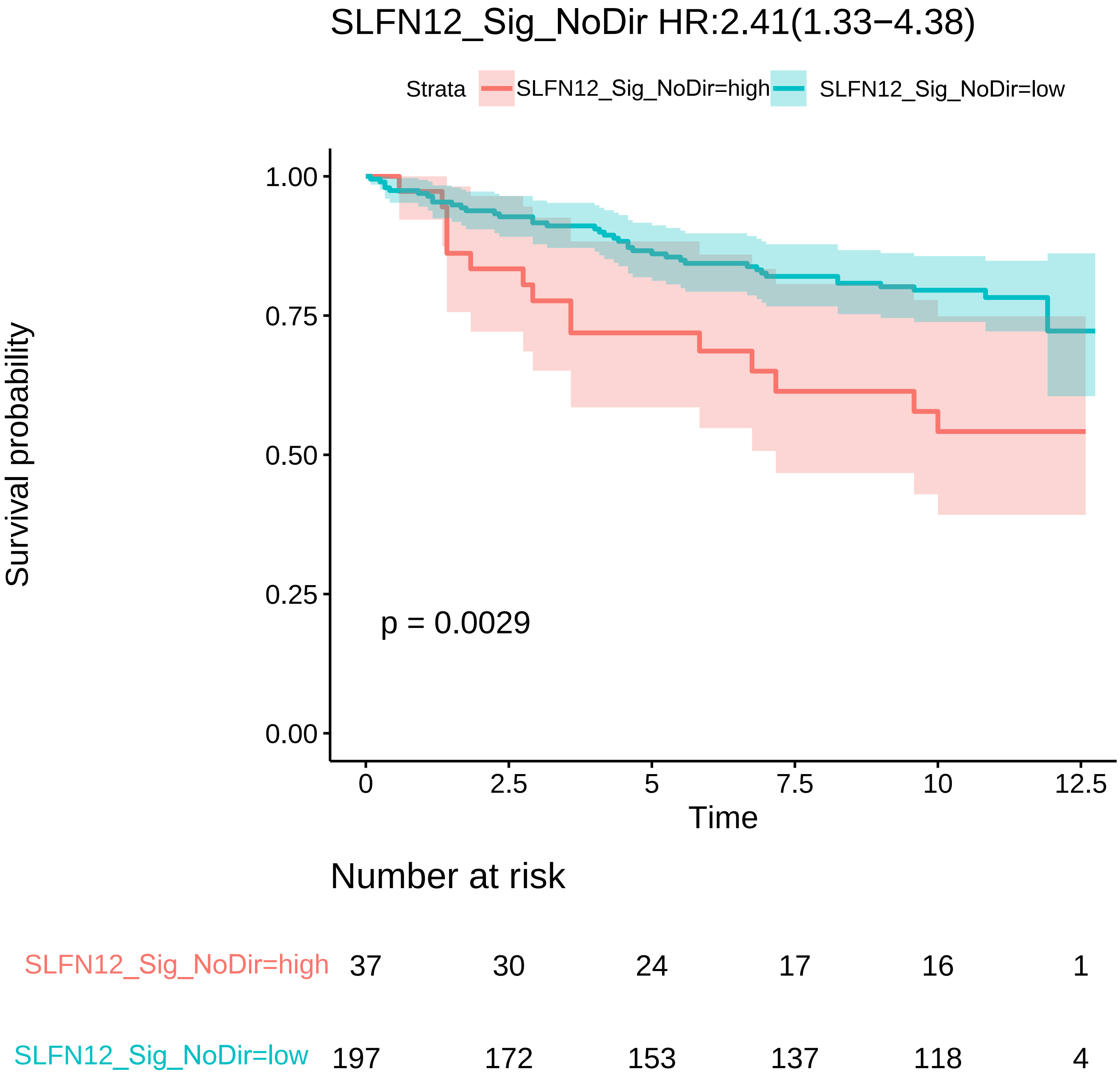
SLFN12_Sig_NoDir HR:2.41(1.33−4.38)
Strata
SLFN12_Sig_NoDir=high
SLFN12_Sig_NoDir=low
Survival probability
1.00
0.75
0.50
0.25
0.00
p = 0.0029
0
2.5
5
7.5
10
12.5
Time
Number at risk
SLFN12_Sig_NoDir=high 37 30 24 17 16 1
SLFN12_Sig_NoDir=low 197 172 153 137 118 4

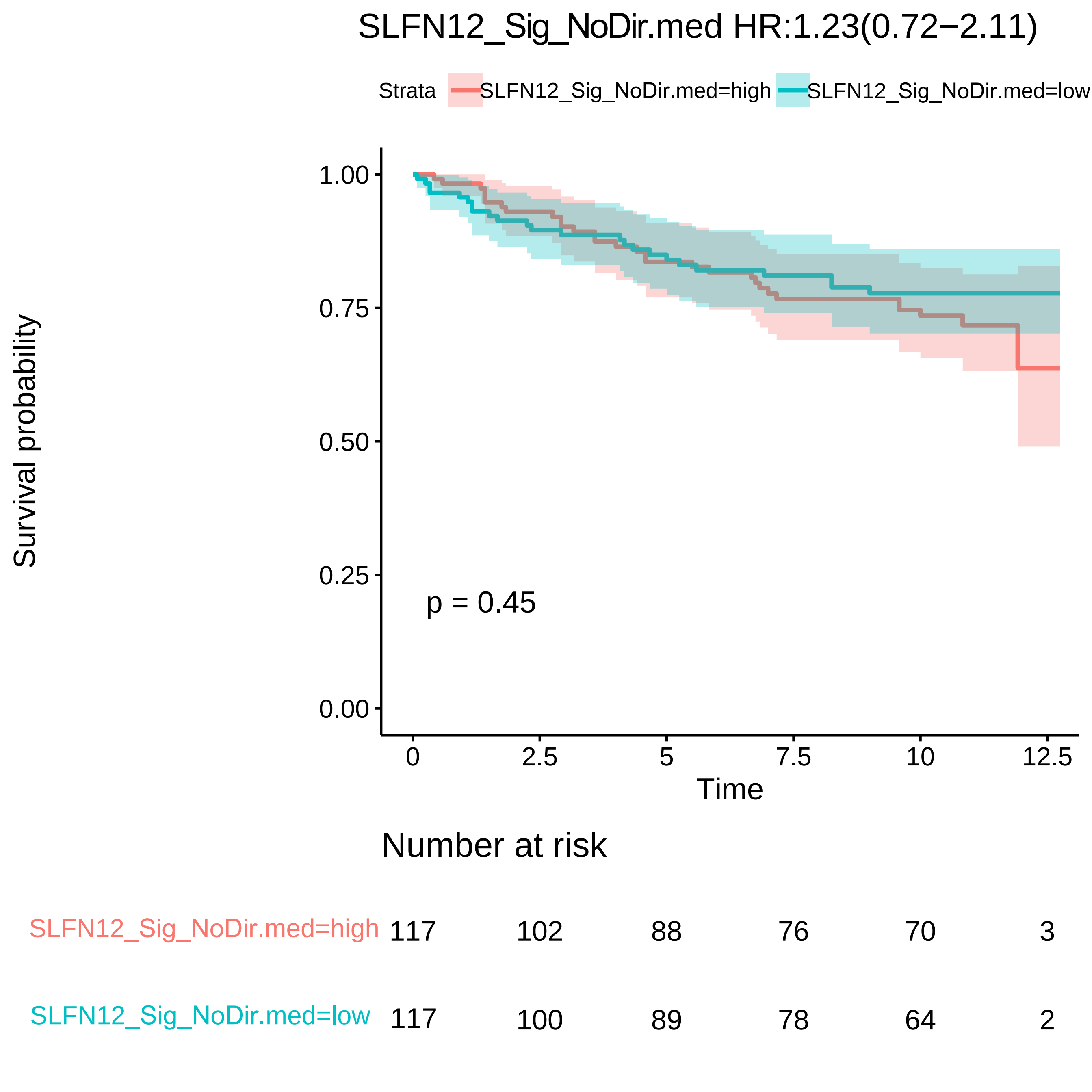
SLFN12_Sig_NoDir.med HR:1.23(0.72−2.11)
Strata
SLFN12_Sig_NoDir.med=high
SLFN12_Sig_NoDir.med=low
Survival probability
1.00
0.75
0.50
0.25
0.00
p = 0.45
0
2.5
5
7.5
10
12.5
Time
Number at risk
SLFN12_Sig_NoDir.med=high 117 102 88 76 70 3
SLFN12_Sig_NoDir.med=low 117 100 89 78 64 2

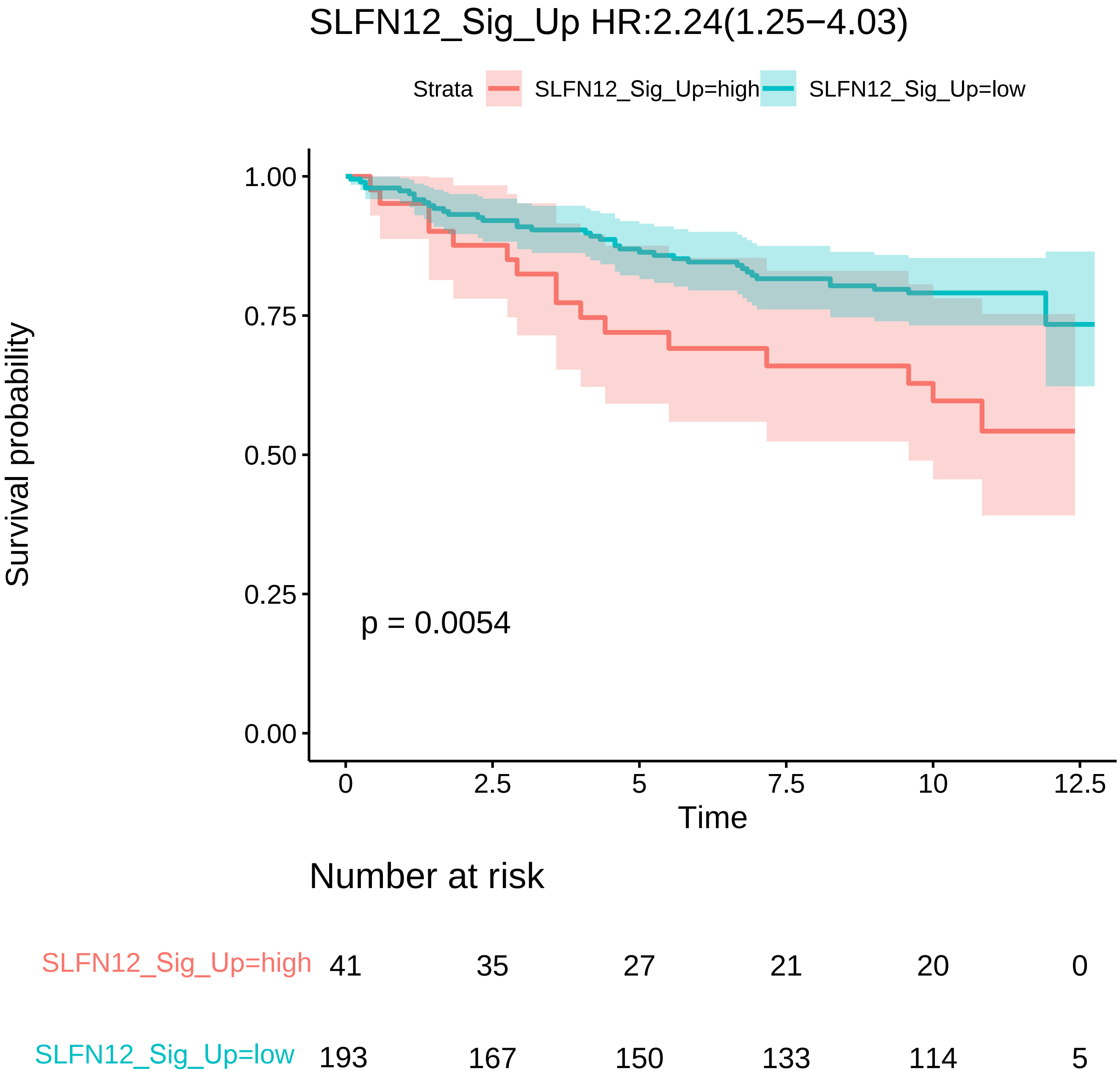

SLFN12_Sig_Up HR:2.24(1.25−4.03)
Strata
SLFN12_Sig_Up=high
SLFN12_Sig_Up=low
Survival probability
1.00
0.75
0.50
0.25
0.00
p = 0.0054
0
2.5
5
7.5
10
12.5
Time
Number at risk
SLFN12_Sig_Up=high 41 35 27 21 20 0
SLFN12_Sig_Up=low 193 167 150 133 114 5

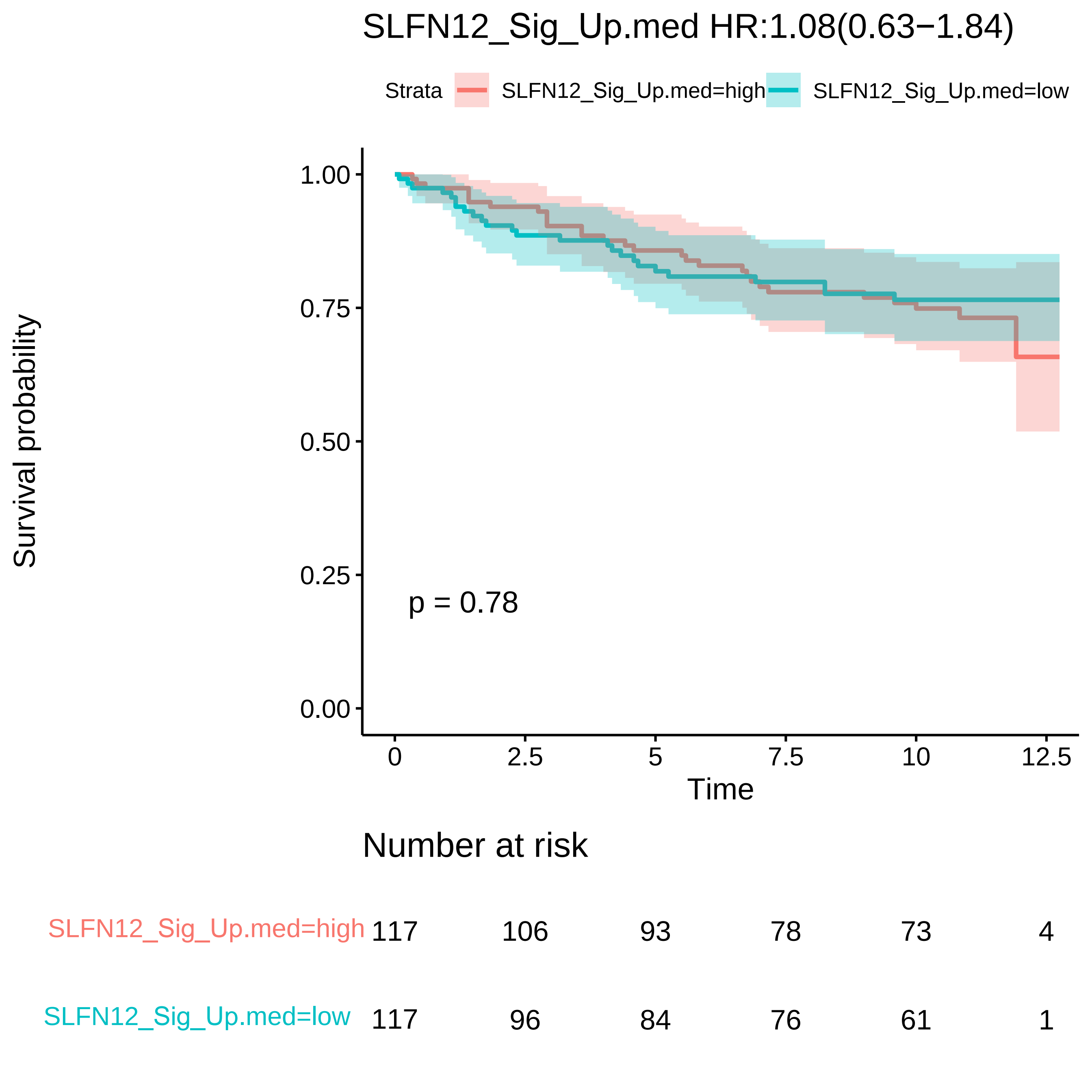
SLFN12_Sig_Up.med HR:1.08(0.63−1.84)
Strata
SLFN12_Sig_Up.med=high
SLFN12_Sig_Up.med=low
Survival probability
1.00
0.75
0.50
0.25
0.00
p = 0.78
0
2.5
5
7.5
10
12.5
Time
Number at risk
SLFN12_Sig_Up.med=high 117 106 93 78 73 4
SLFN12_Sig_Up.med=low 117 96 84 76 61 1

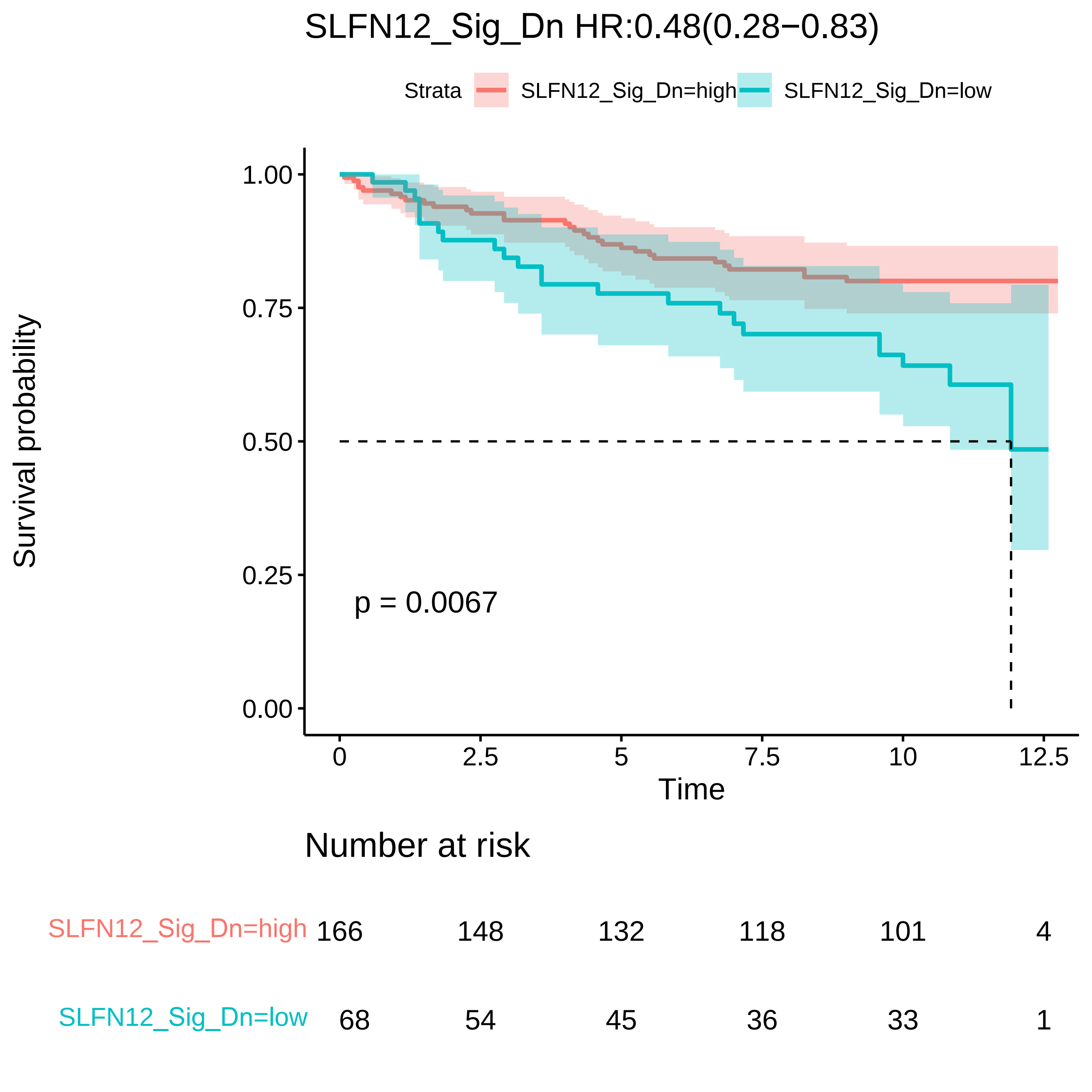
SLFN12_Sig_Dn HR:0.48(0.28−0.83)
Strata
SLFN12_Sig_Dn=high
SLFN12_Sig_Dn=low
Survival probability
1.00
0.75
0.50
0.25
0.00
p = 0.0067
0
2.5
5
7.5
10
12.5
Time
Number at risk
SLFN12_Sig_Dn=high 166 148 132 118 101 4
SLFN12_Sig_Dn=low 68 54 45 36 33 1

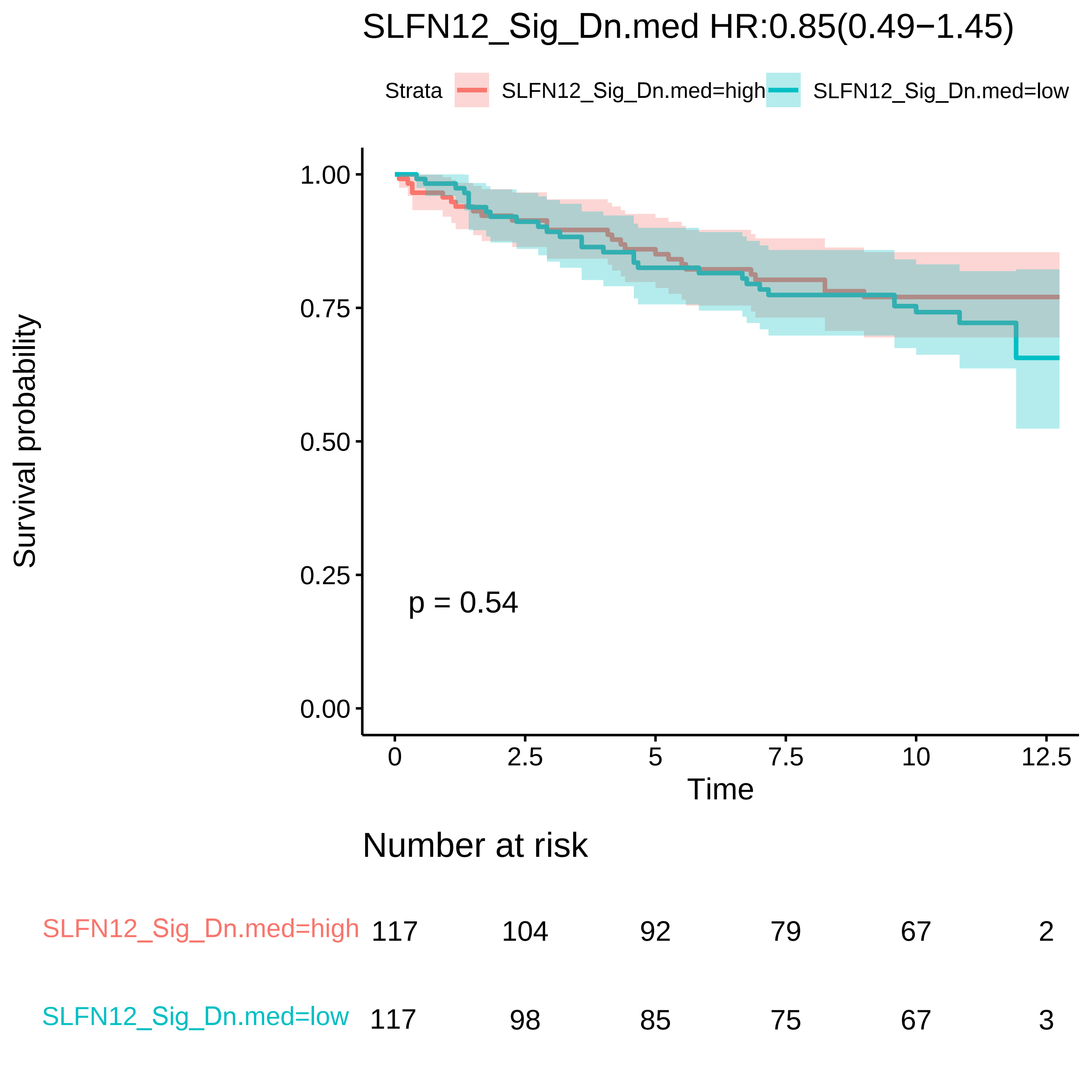
SLFN12_Sig_Dn.med HR:0.85(0.49−1.45)
Strata
SLFN12_Sig_Dn.med=high
SLFN12_Sig_Dn.med=low
Survival probability
1.00
0.75
0.50
0.25
0.00
p = 0.54
0
2.5
5
7.5
10
12.5
Time
Number at risk
SLFN12_Sig_Dn.med=high 117 104 92 79 67 2
SLFN12_Sig_Dn.med=low 117 98 85 75 67 3
