# Supplementary material for: Schlafen 12 Slows TNBC Tumor Growth, Induces Luminal Markers, and Predicts Favorable Survival
Source: Cancers (Basel). 2023 Jan 7;15(2):402. doi: 10.3390/cancers15020402 (PMC9856841; doi:10.3390/cancers15020402)
Supplement: Supplementary file 1 [file cancers-15-00402-s001.zip › Supplemental Figure 5 (1).pdf]

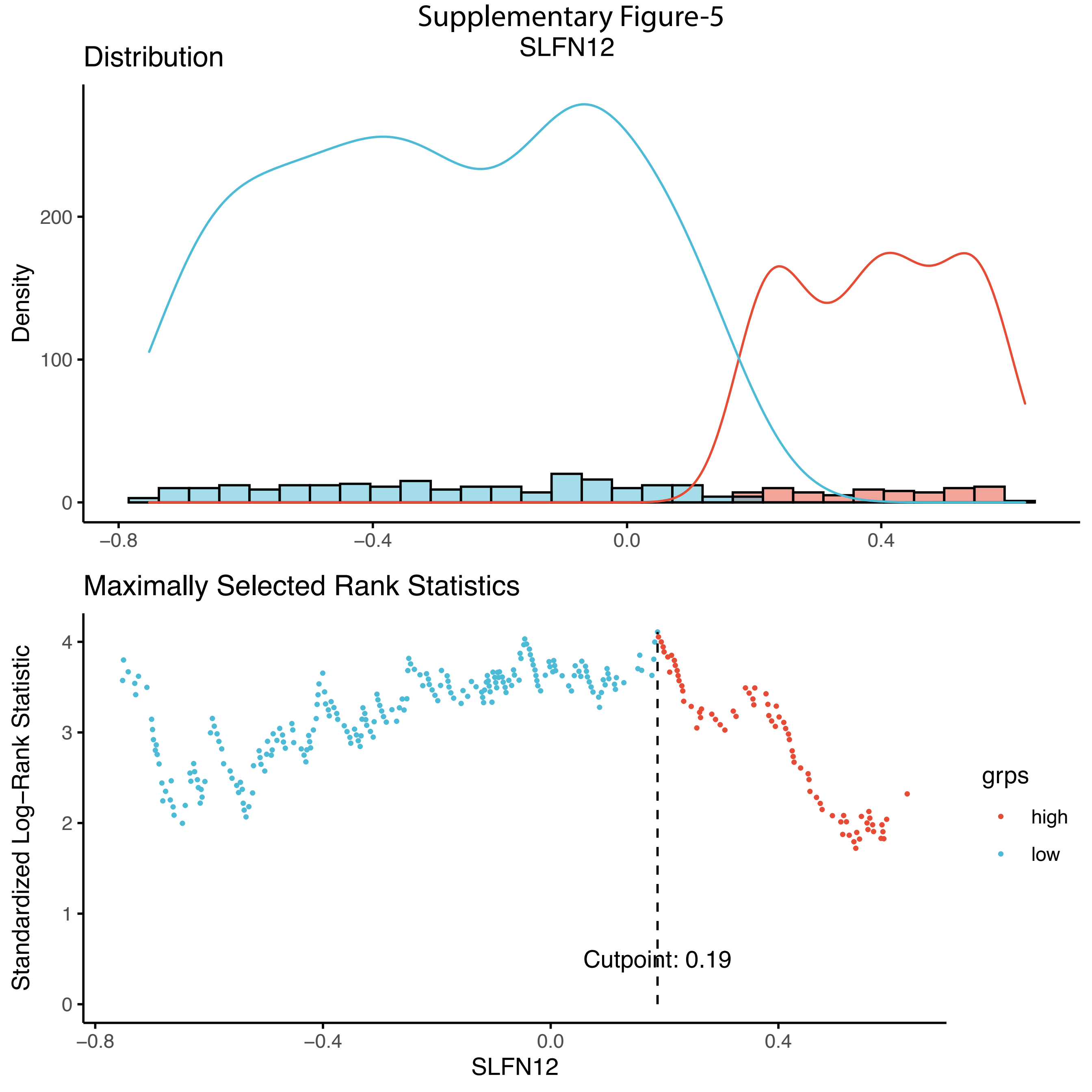
Supplementary Figure-5
SLFN12
Distribution
Density
200
100
0
−0.8
−0.4
0.0
0.4
Maximally Selected Rank Statistics
Standardized Log−Rank Statistic
4
3
2
1
0
Cutpoint: 0.19
−0.8
−0.4
0.0
0.4
SLFN12
grps
high
low

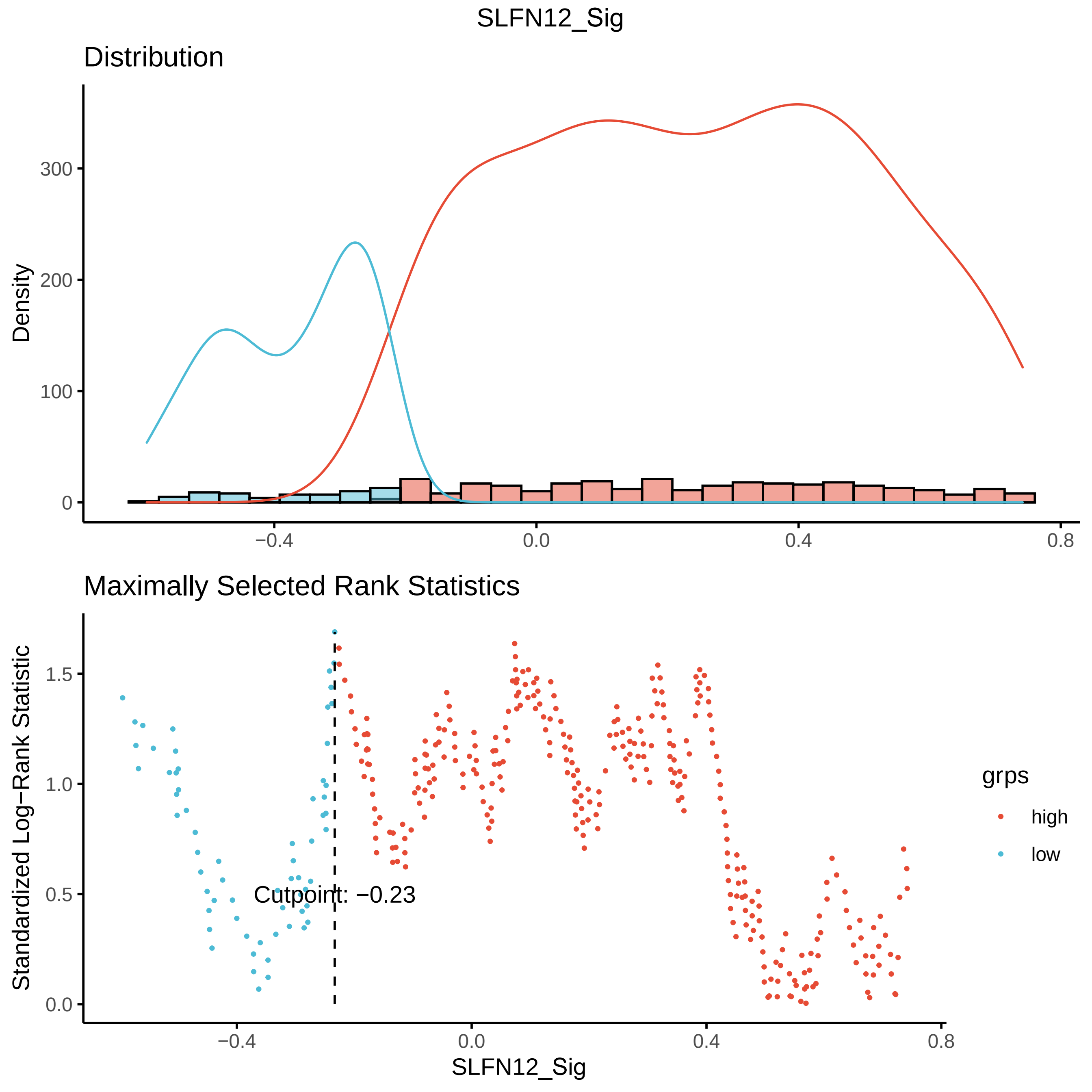

SLFN12_Sig
Distribution
Density
300
200
100
0
−0.4
0.0
0.4
0.8
Maximally Selected Rank Statistics
Standardized Log−Rank Statistic
1.5
1.0
0.5
0.0
Cutpoint: −0.23
−0.4
0.0
0.4
0.8
SLFN12_Sig
grps
high
low

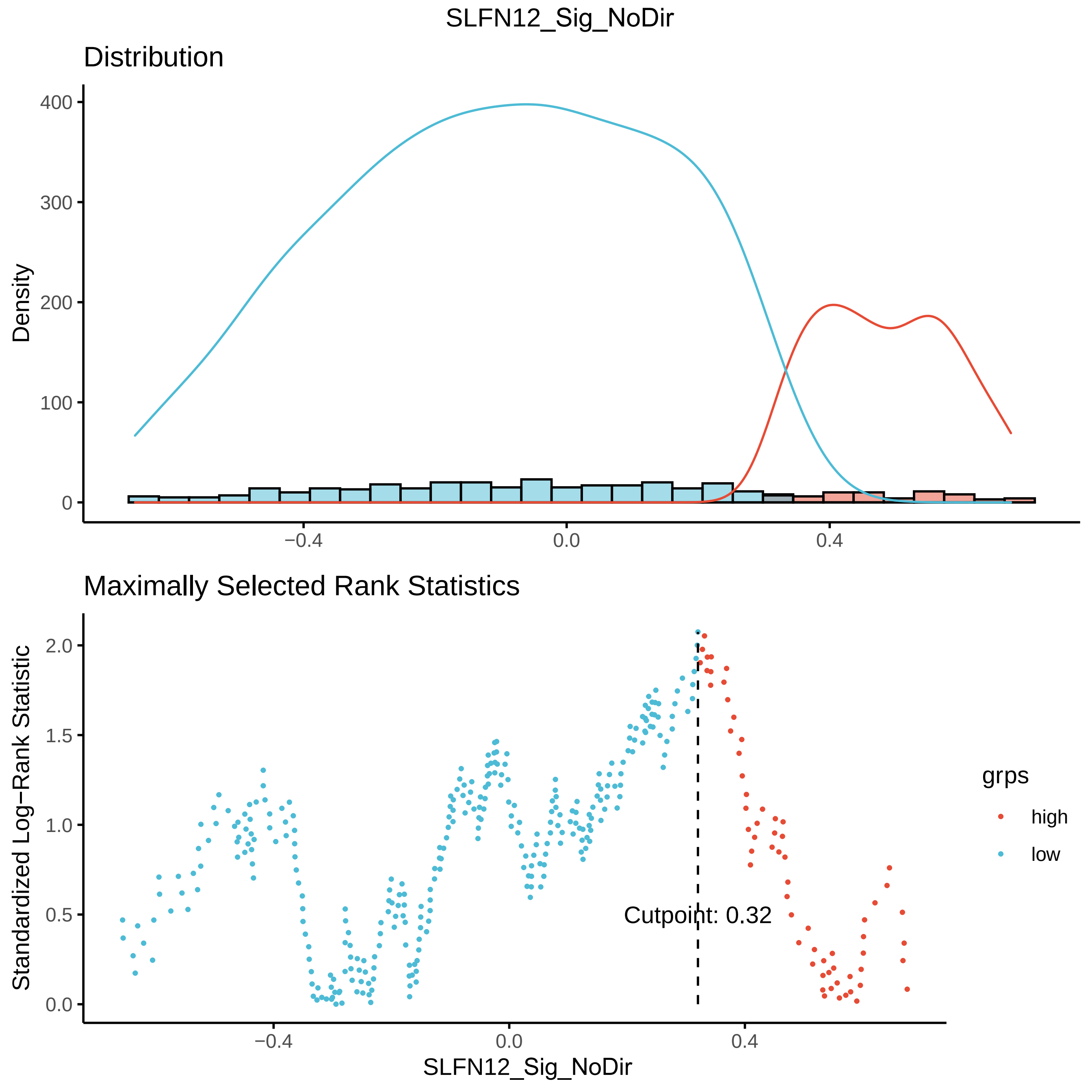
SLFN12_Sig_NoDir
Distribution
Density
400
300
200
100
0
−0.4
0.0
0.4
Maximally Selected Rank Statistics
Standardized Log−Rank Statistic
2.0
1.5
1.0
0.5
0.0
Cutpoint: 0.32
−0.4
0.0
0.4
SLFN12_Sig_NoDir
grps
high
low

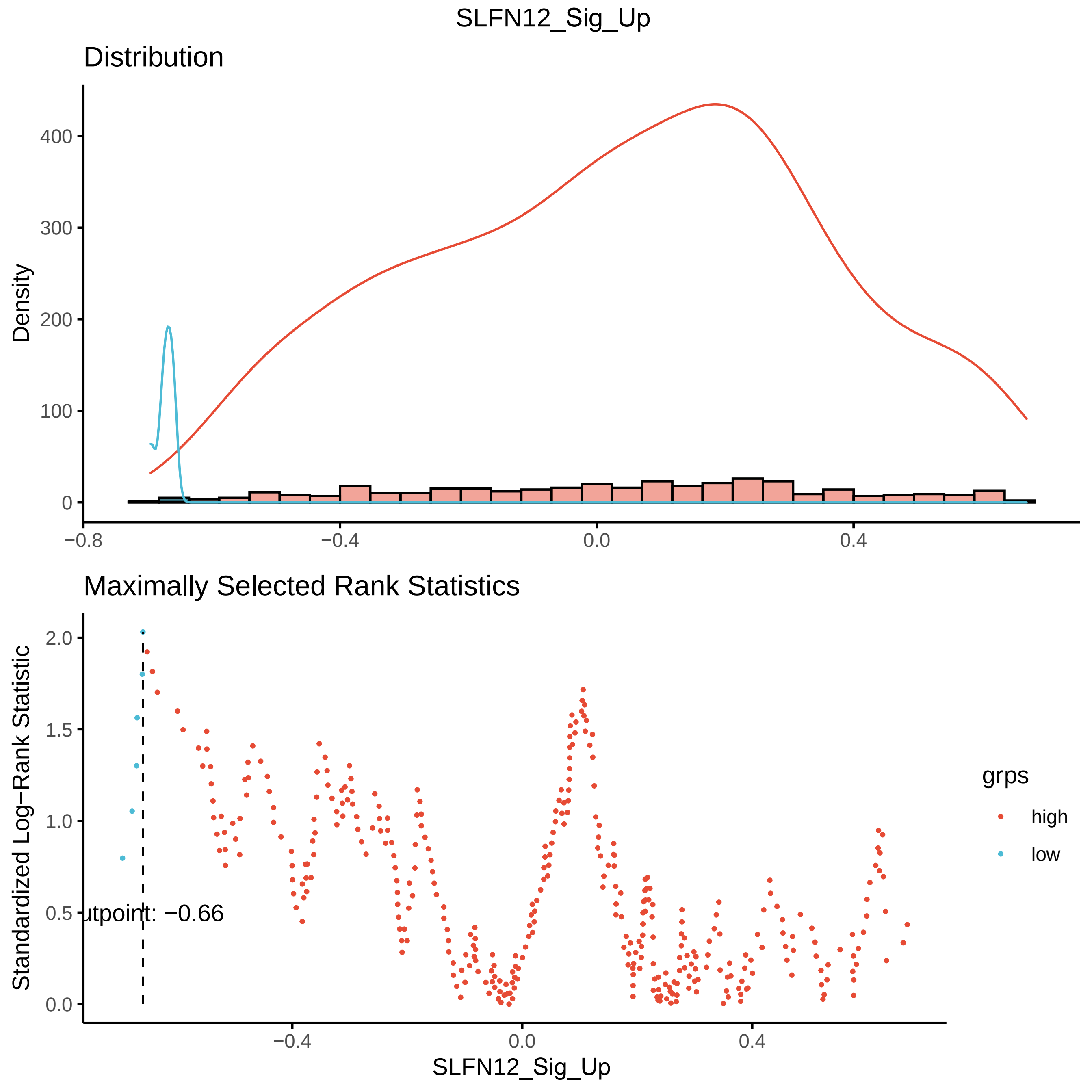

SLFN12_Sig_Up
Distribution
Density
400
300
200
100
0
−0.8
−0.4
0.0
0.4
Maximally Selected Rank Statistics
Standardized Log−Rank Statistic
2.0
1.5
1.0
0.5
0.0
utpoint: −0.66
−0.4
0.0
0.4
SLFN12_Sig_Up
grps
high
low

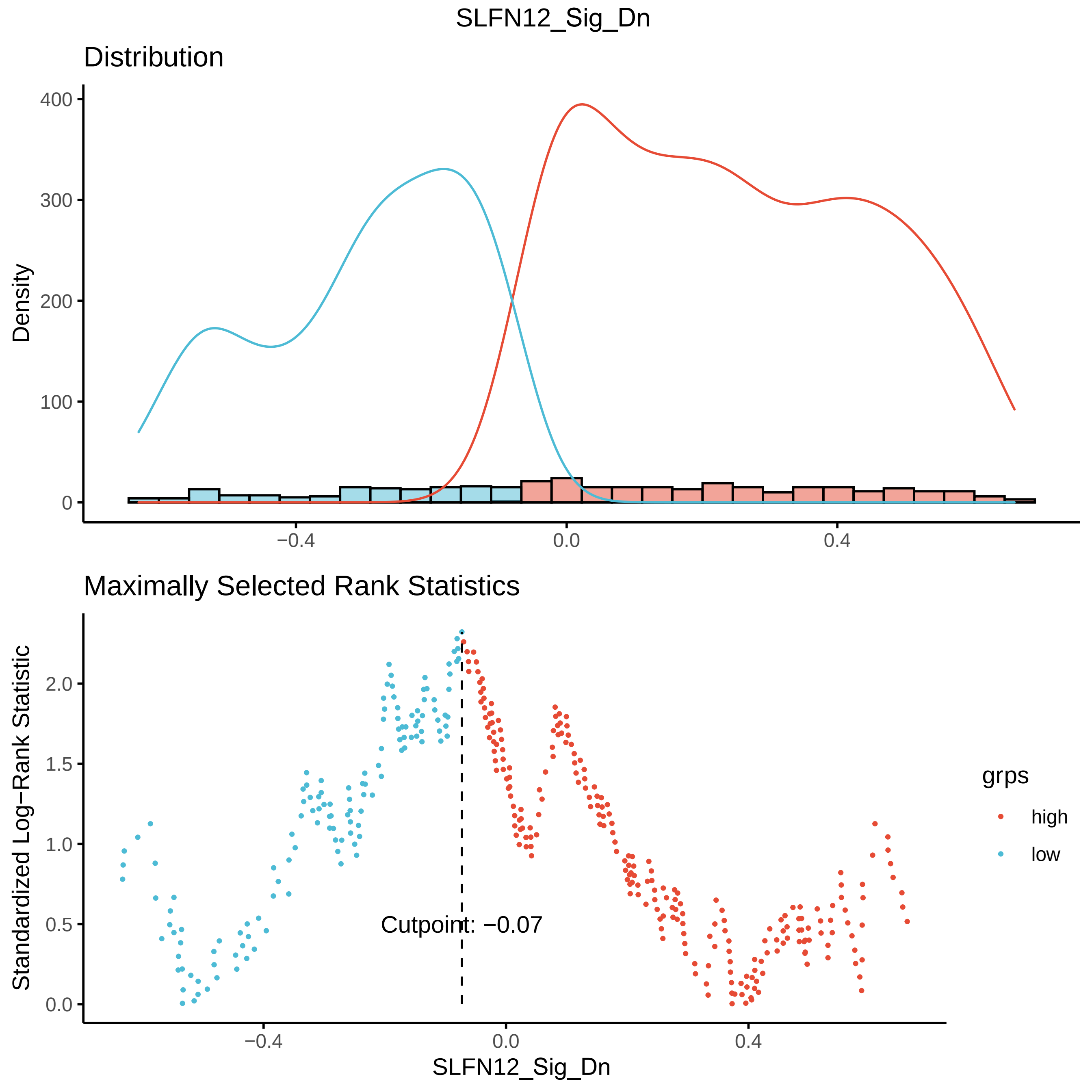

SLFN12_Sig_Dn
Distribution
Density
400
300
200
100
0
−0.4
0.0
0.4
Maximally Selected Rank Statistics
Standardized Log−Rank Statistic
2.0
1.5
1.0
0.5
0.0
Cutpoint: −0.07
−0.4
0.0
0.4
SLFN12_Sig_Dn
grps
high
low

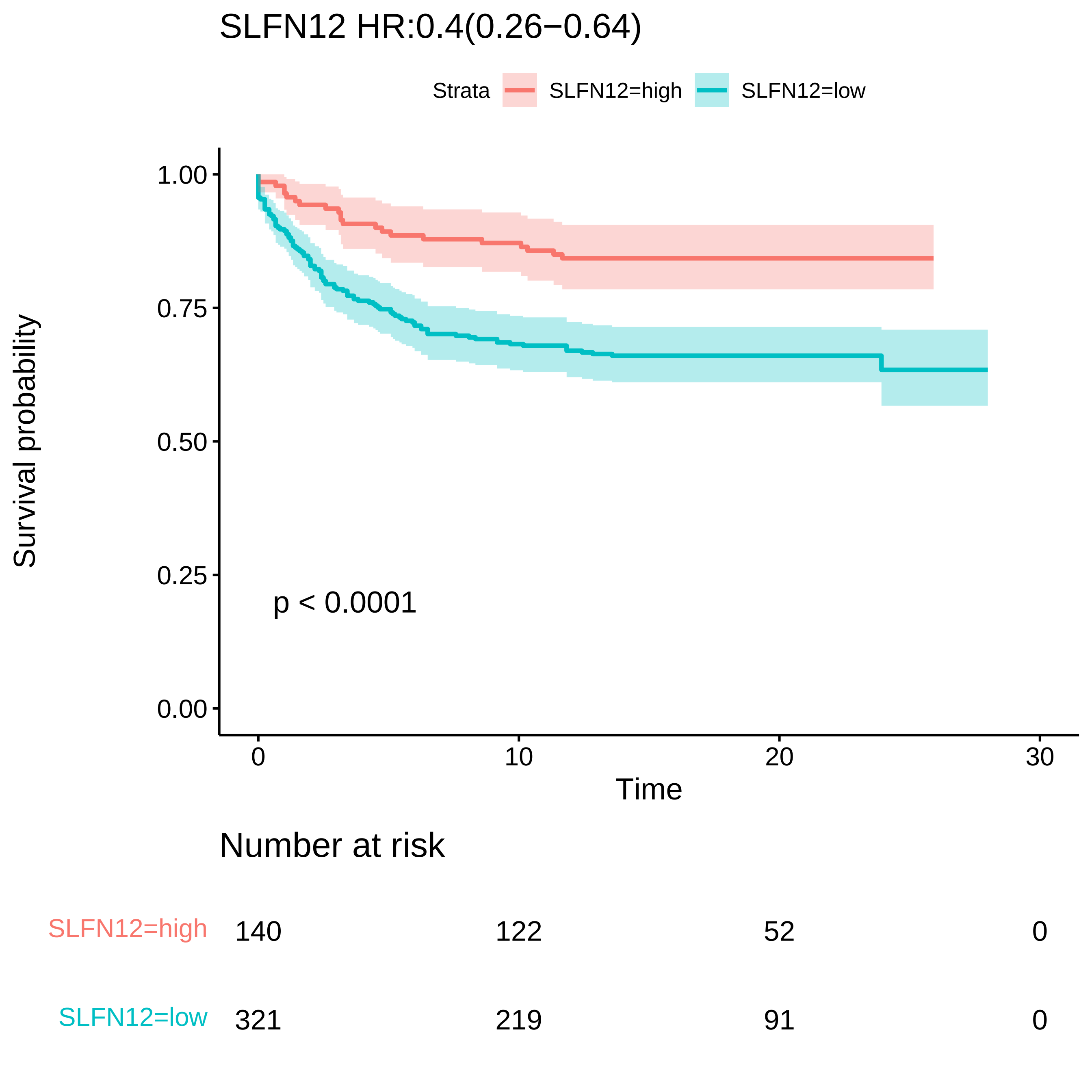
SLFN12 HR:0.4(0.26−0.64)
Strata
SLFN12=high
SLFN12=low
Survival probability
1.00
0.75
0.50
0.25
0.00
p < 0.0001
0
10
20
30
Time
Number at risk
SLFN12=high
140
122
52
0
SLFN12=low
321
219
91
0

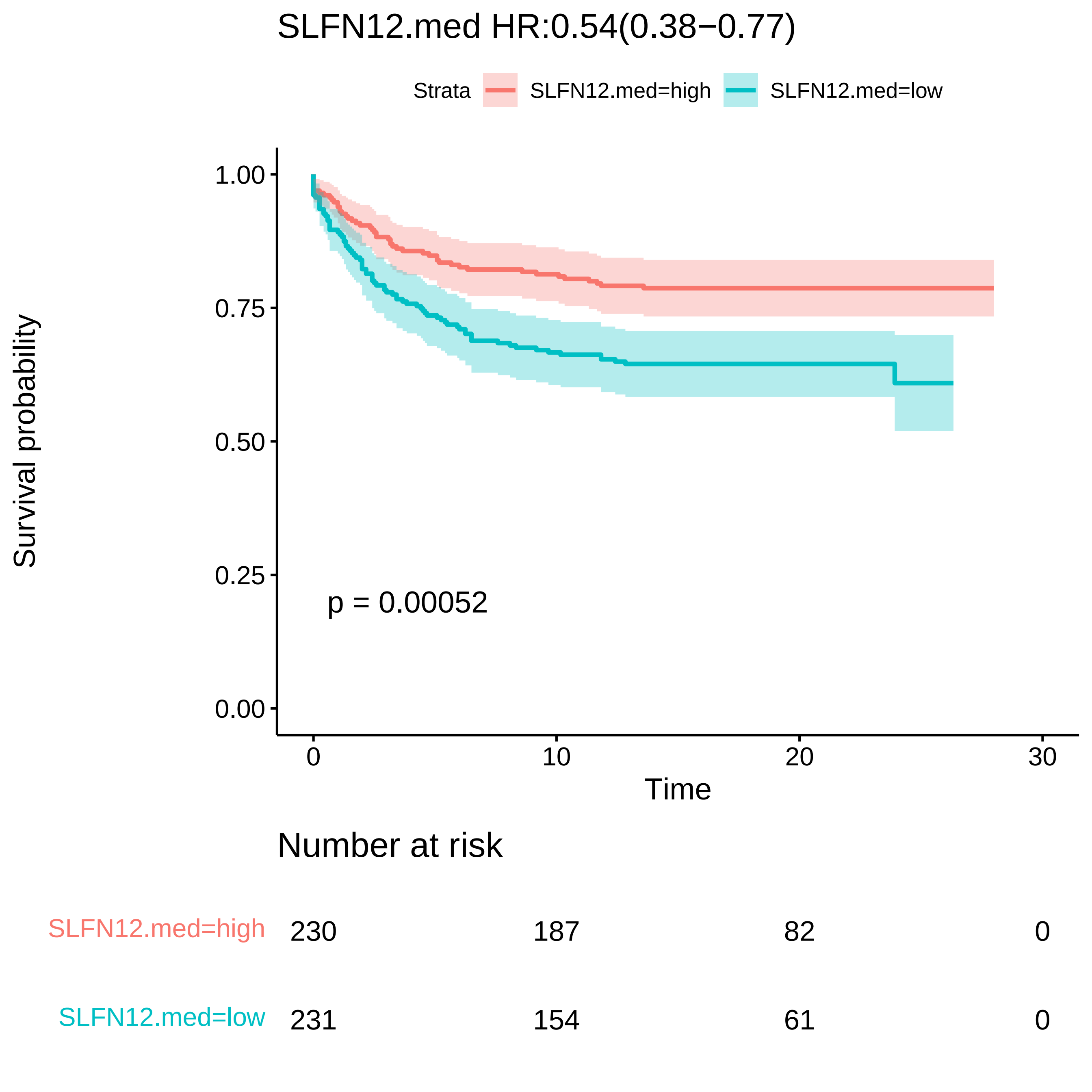
SLFN12.med HR:0.54(0.38−0.77)
Strata
SLFN12.med=high
SLFN12.med=low
Survival probability
1.00
0.75
0.50
0.25
0.00
p = 0.00052
0
10
20
30
Time
Number at risk
SLFN12.med=high 230 187 82 0
SLFN12.med=low 231 154 61 0

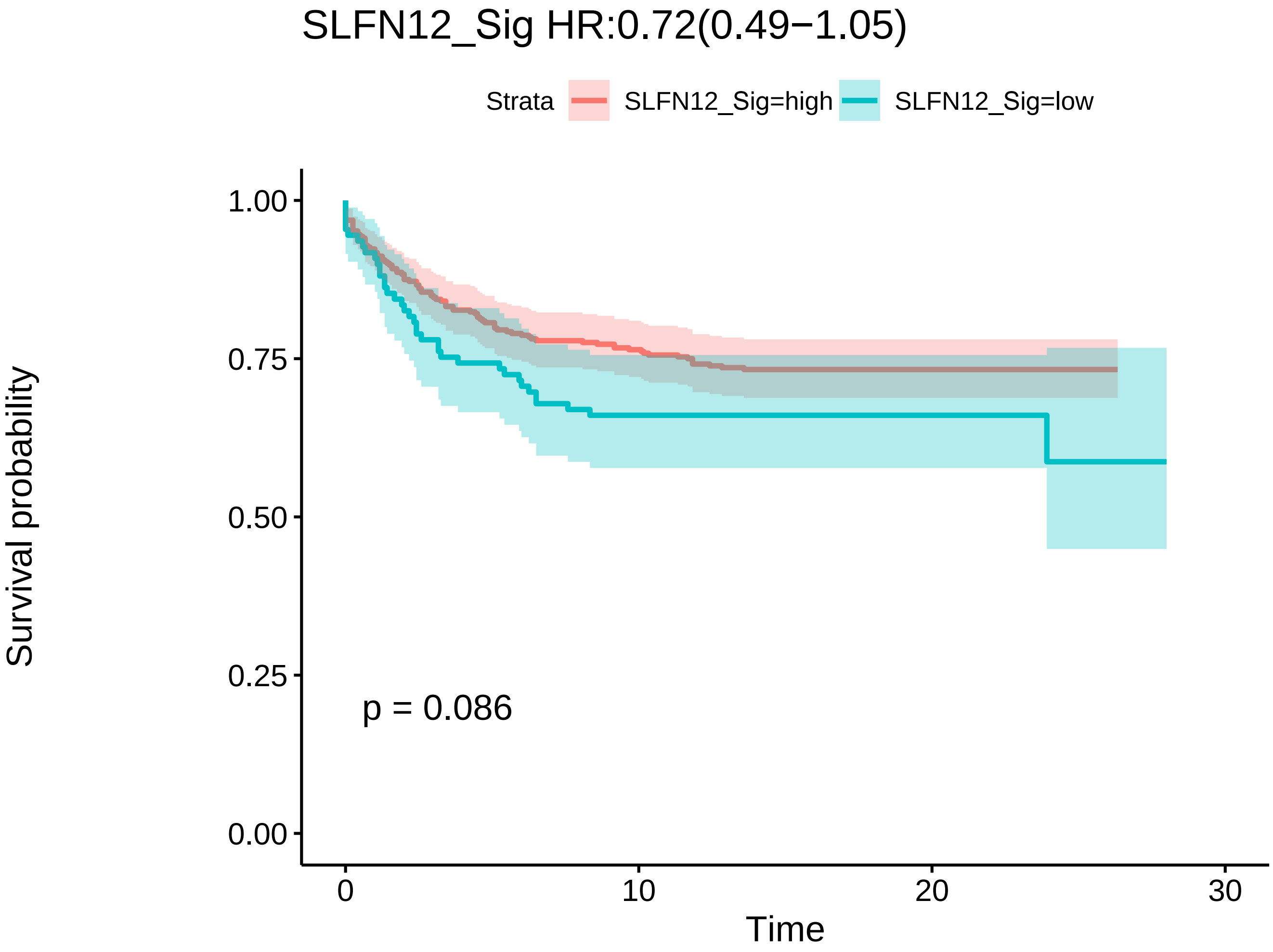

Number at risk

	0	10	20	30
SLFN12_Sig=high	352	269	108	0
SLFN12_Sig=low	109	72	35	0

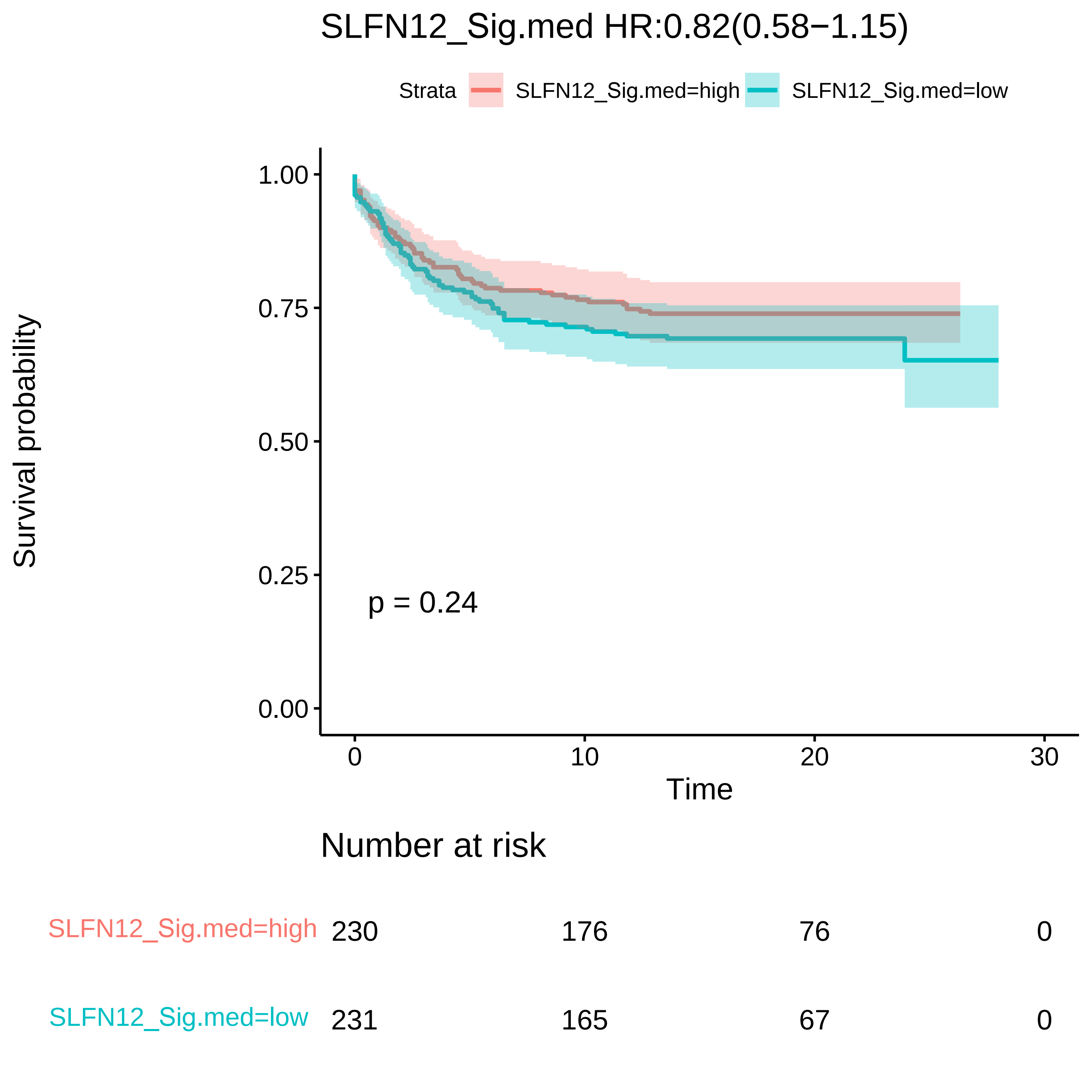
SLFN12_Sig.med HR:0.82(0.58−1.15)
Strata
SLFN12_Sig.med=high
SLFN12_Sig.med=low
Survival probability
1.00
0.75
0.50
0.25
0.00
p = 0.24
0
10
20
30
Time
Number at risk
SLFN12_Sig.med=high 230 176 76 0
SLFN12_Sig.med=low 231 165 67 0

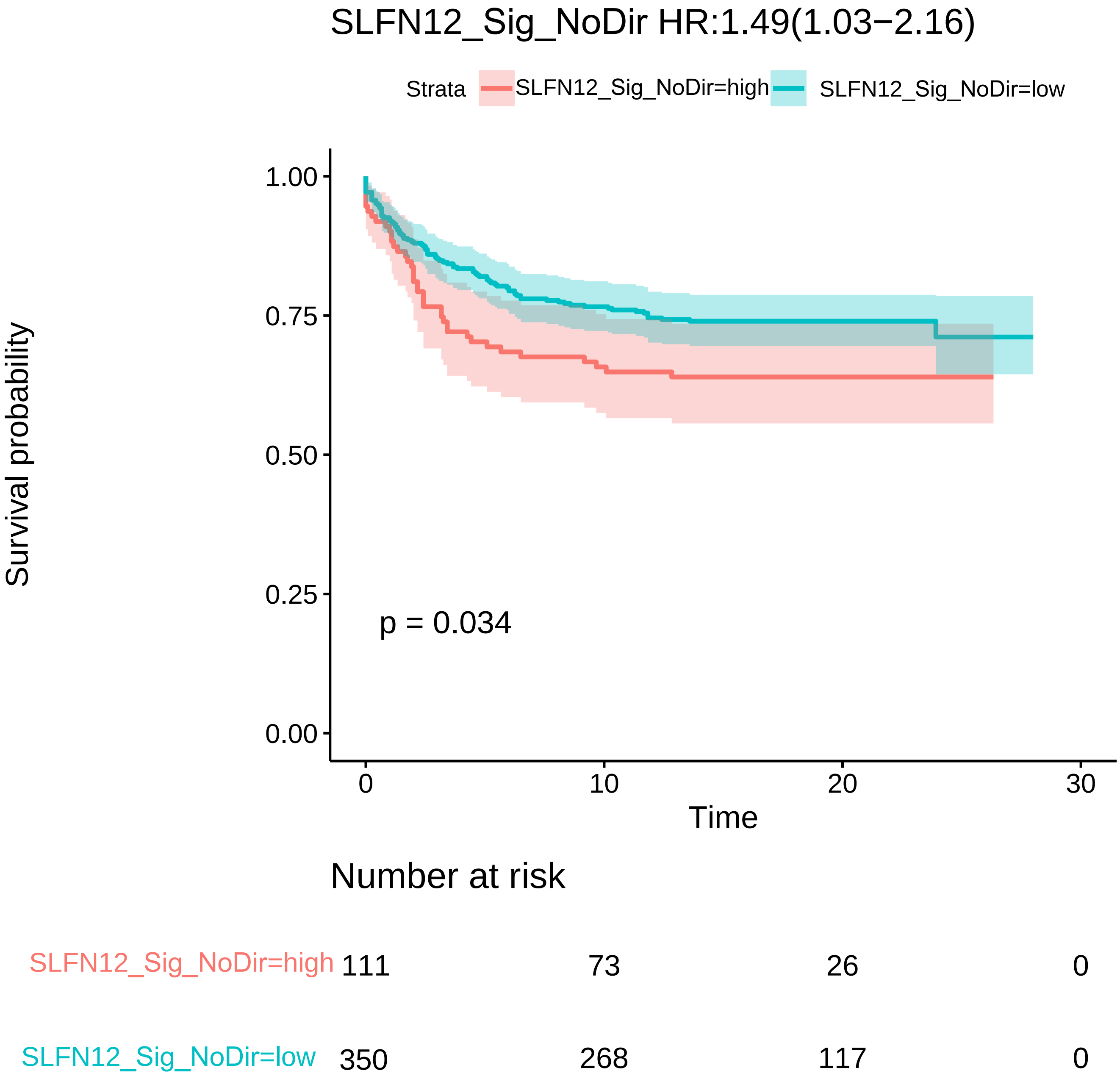
SLFN12_Sig_NoDir HR:1.49(1.03−2.16)
Strata
SLFN12_Sig_NoDir=high
SLFN12_Sig_NoDir=low
Survival probability
1.00
0.75
0.50
0.25
0.00
p = 0.034
0
10
20
30
Time
Number at risk
SLFN12_Sig_NoDir=high 111 73 26 0
SLFN12_Sig_NoDir=low 350 268 117 0

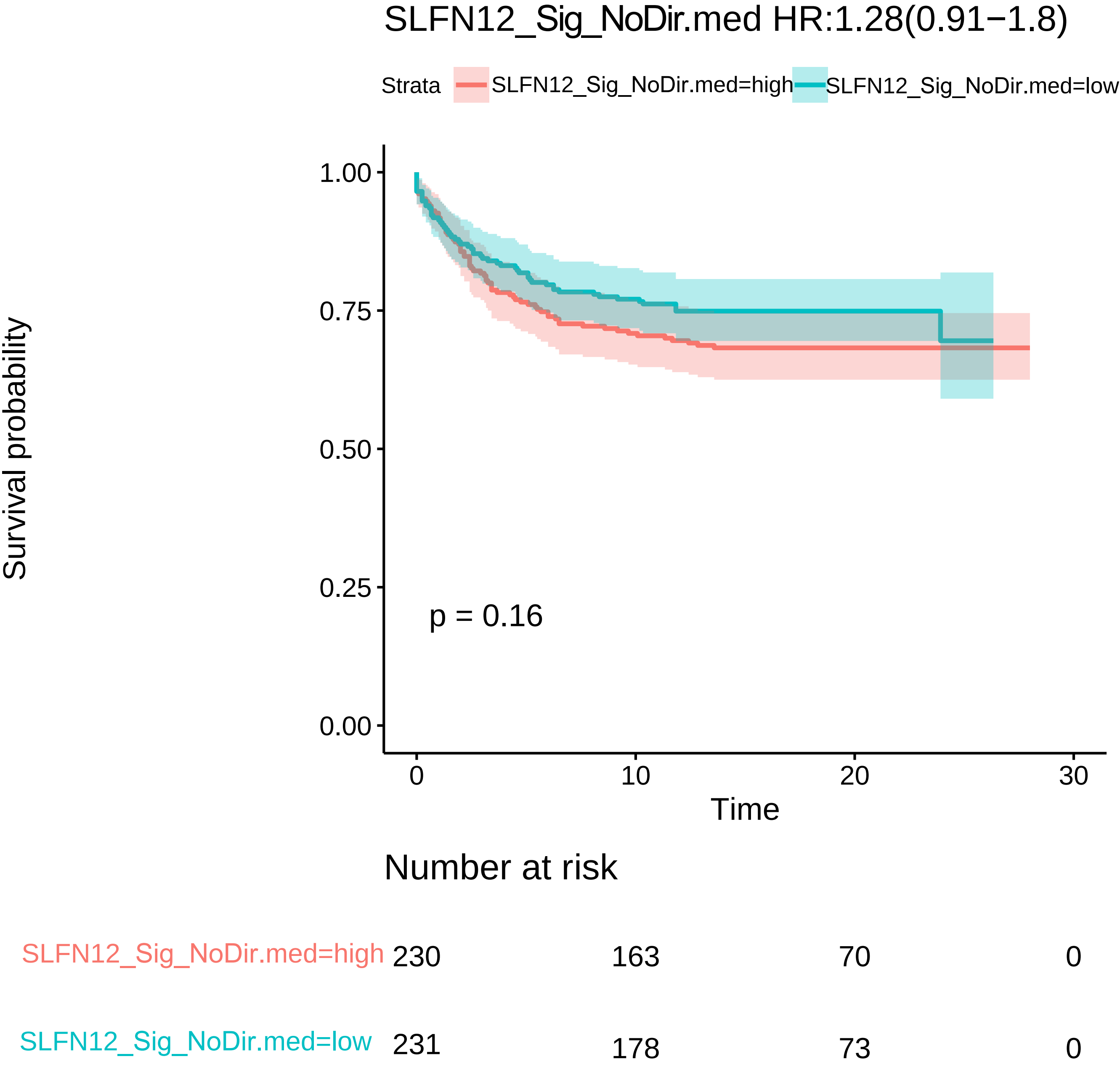
SLFN12_Sig_NoDir.med HR:1.28(0.91−1.8)
Strata
SLFN12_Sig_NoDir.med=high
SLFN12_Sig_NoDir.med=low
Survival probability
1.00
0.75
0.50
0.25
0.00
p = 0.16
0
10
20
30
Time
Number at risk
SLFN12_Sig_NoDir.med=high 230 163 70 0
SLFN12_Sig_NoDir.med=low 231 178 73 0

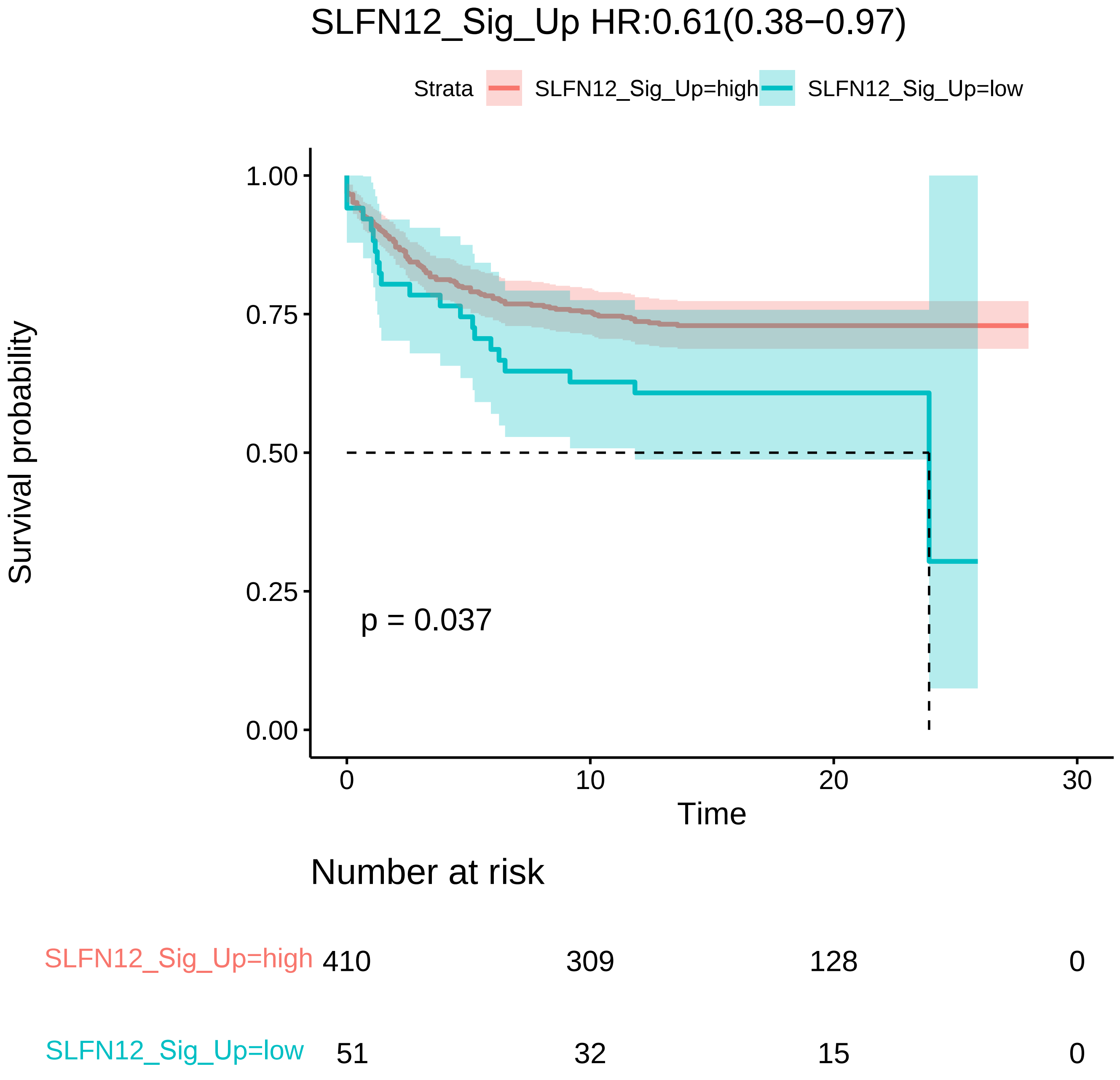
SLFN12_Sig_Up HR:0.61(0.38−0.97)
Strata
SLFN12_Sig_Up=high
SLFN12_Sig_Up=low
Survival probability
1.00
0.75
0.50
0.25
0.00
p = 0.037
0
10
20
30
Time
Number at risk
SLFN12_Sig_Up=high
410
309
128
0
SLFN12_Sig_Up=low
51
32
15
0

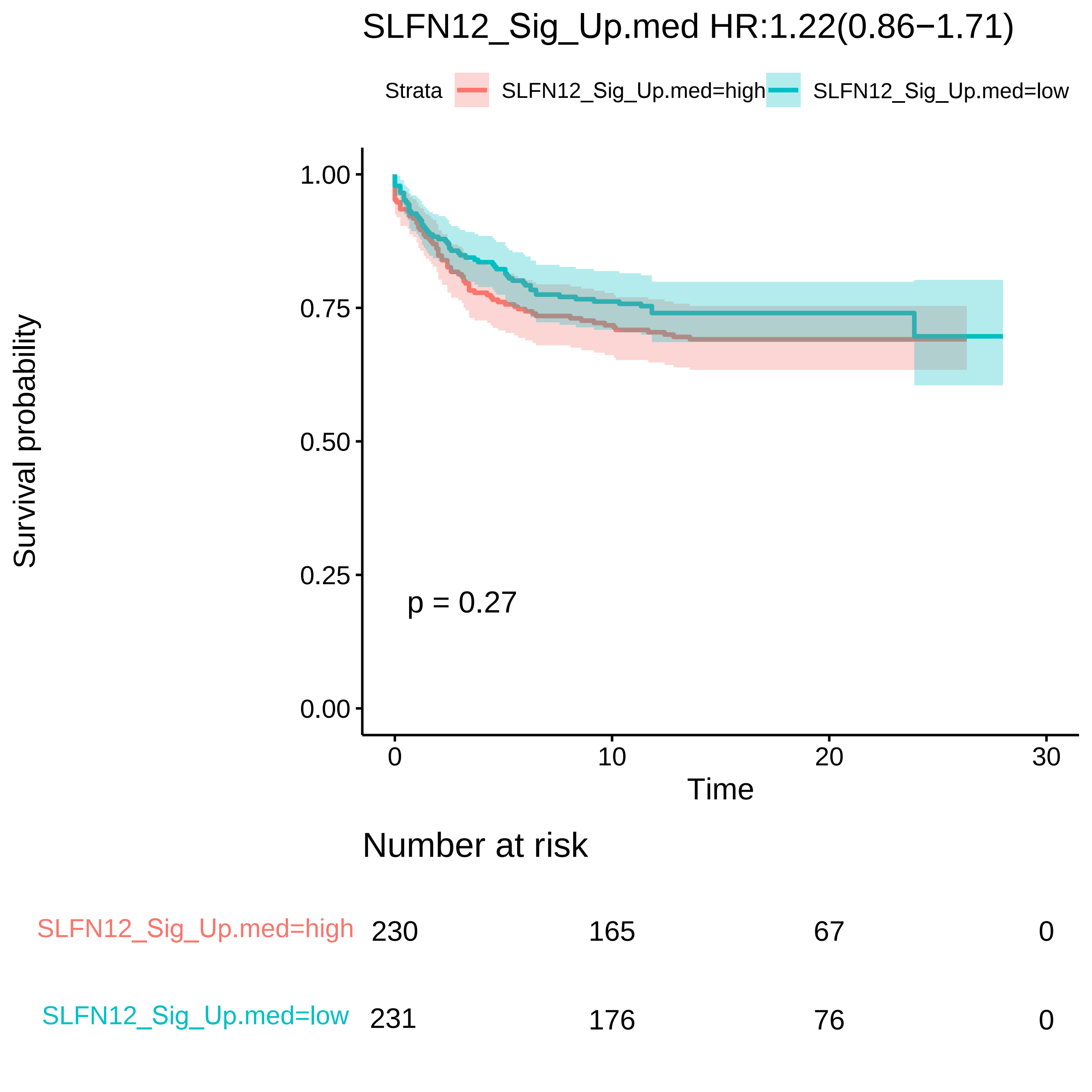

SLFN12_Sig_Up.med HR:1.22(0.86−1.71)
Strata
SLFN12_Sig_Up.med=high
SLFN12_Sig_Up.med=low
Survival probability
1.00
0.75
0.50
0.25
0.00
p = 0.27
0
10
20
30
Time
Number at risk
SLFN12_Sig_Up.med=high 230 165 67 0
SLFN12_Sig_Up.med=low 231 176 76 0

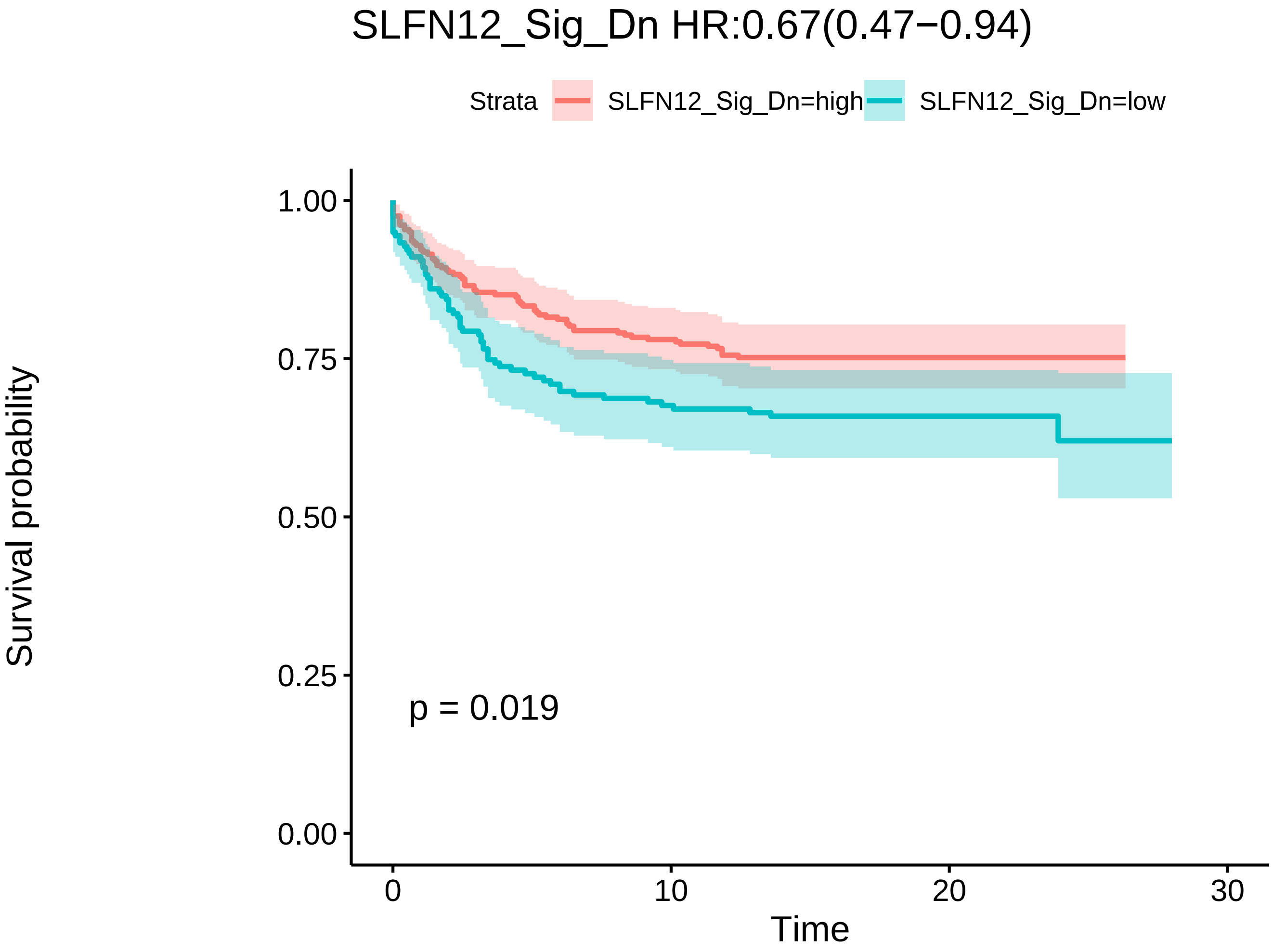

Number at risk

	0	10	20	30
SLFN12_Sig_Dn=high	282	220	91	0
SLFN12_Sig_Dn=low	179	121	52	0

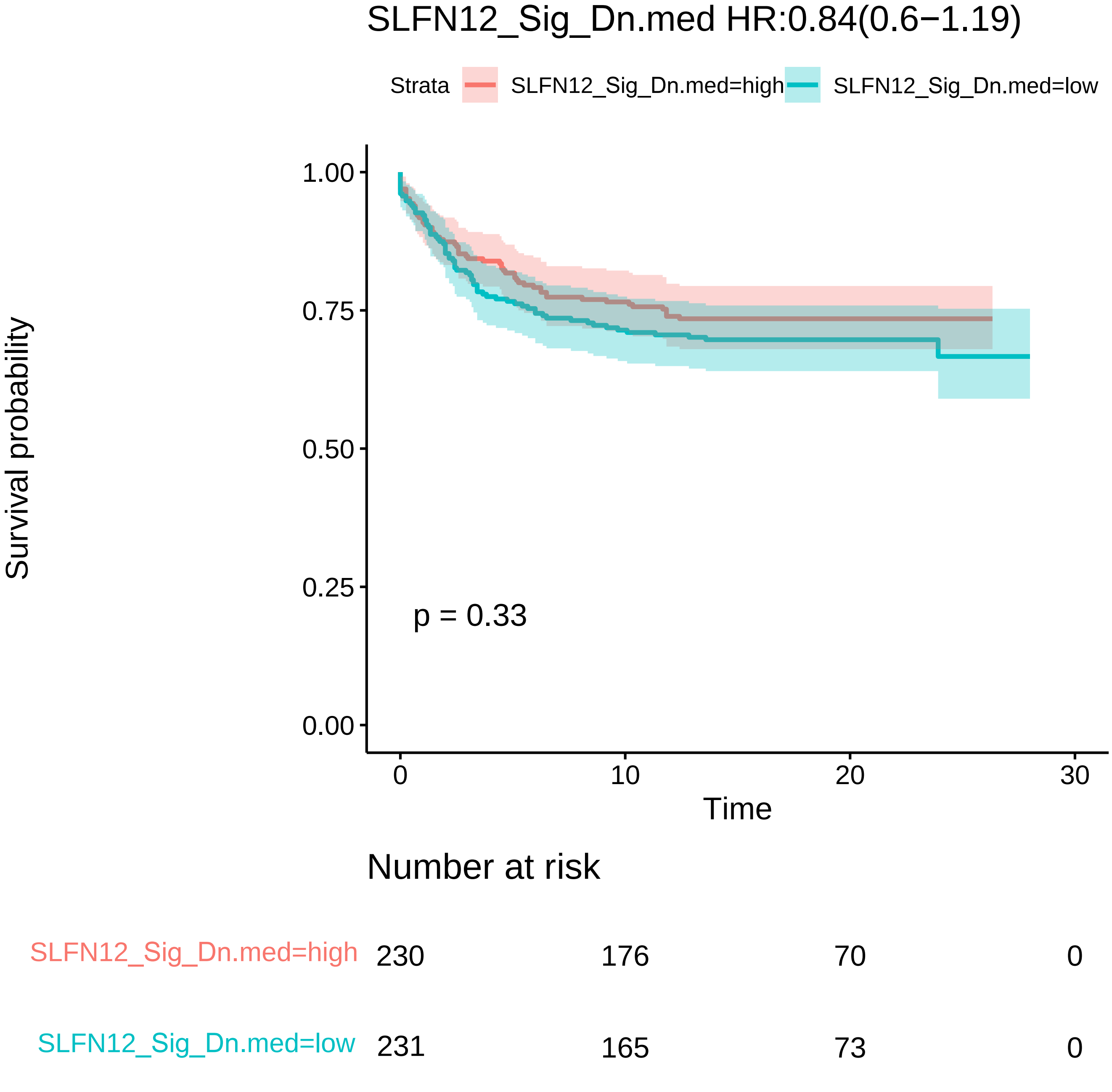
SLFN12_Sig_Dn.med HR:0.84(0.6−1.19)
Strata
SLFN12_Sig_Dn.med=high
SLFN12_Sig_Dn.med=low
Survival probability
1.00
0.75
0.50
0.25
0.00
p = 0.33
0
10
20
30
Time
Number at risk
SLFN12_Sig_Dn.med=high 230 176 70 0
SLFN12_Sig_Dn.med=low 231 165 73 0
