# Supplementary material for: Schlafen 12 Slows TNBC Tumor Growth, Induces Luminal Markers, and Predicts Favorable Survival
Source: Cancers (Basel). 2023 Jan 7;15(2):402. doi: 10.3390/cancers15020402 (PMC9856841; doi:10.3390/cancers15020402)
Supplement: Supplementary file 1 [file cancers-15-00402-s001.zip › Supplemental Figure 6 (1).pdf]

Supplementary Figure-6

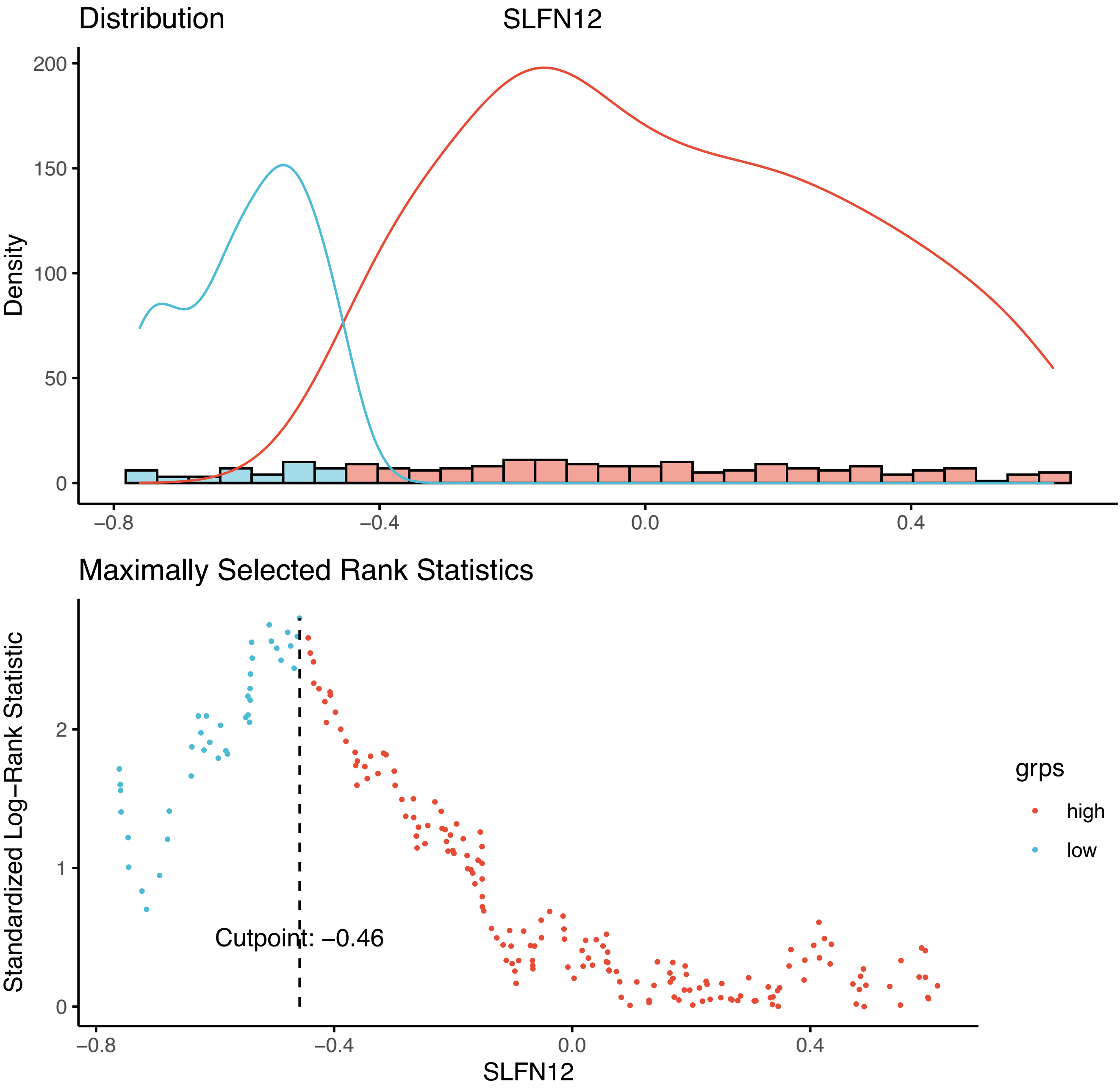

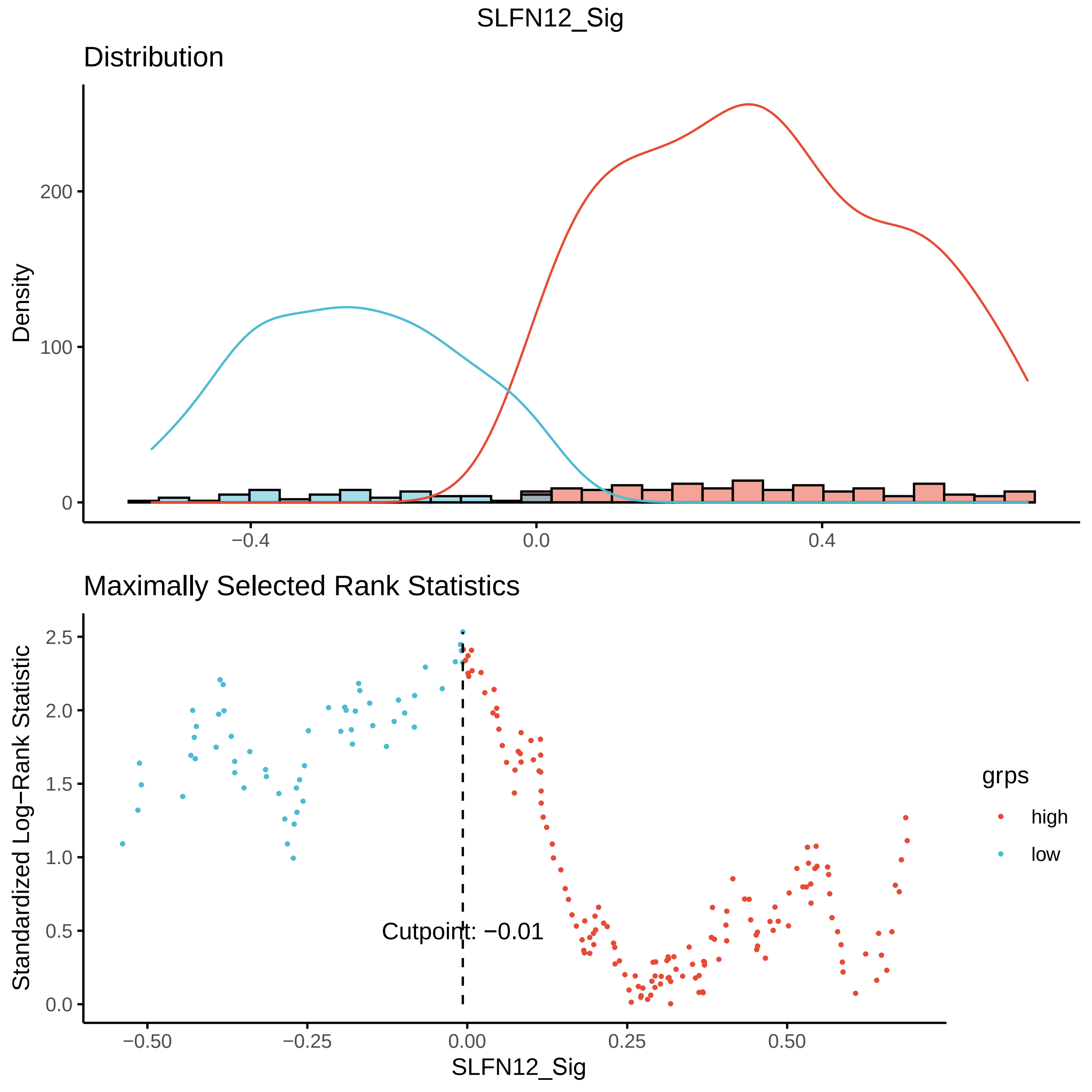

SLFN12_Sig
Distribution
Density
200
100
0
−0.4
0.0
0.4
Maximally Selected Rank Statistics
Standardized Log−Rank Statistic
2.5
2.0
1.5
1.0
0.5
0.0
Cutpoint: −0.01
−0.50
−0.25
0.00
0.25
0.50
SLFN12_Sig
grps
high
low

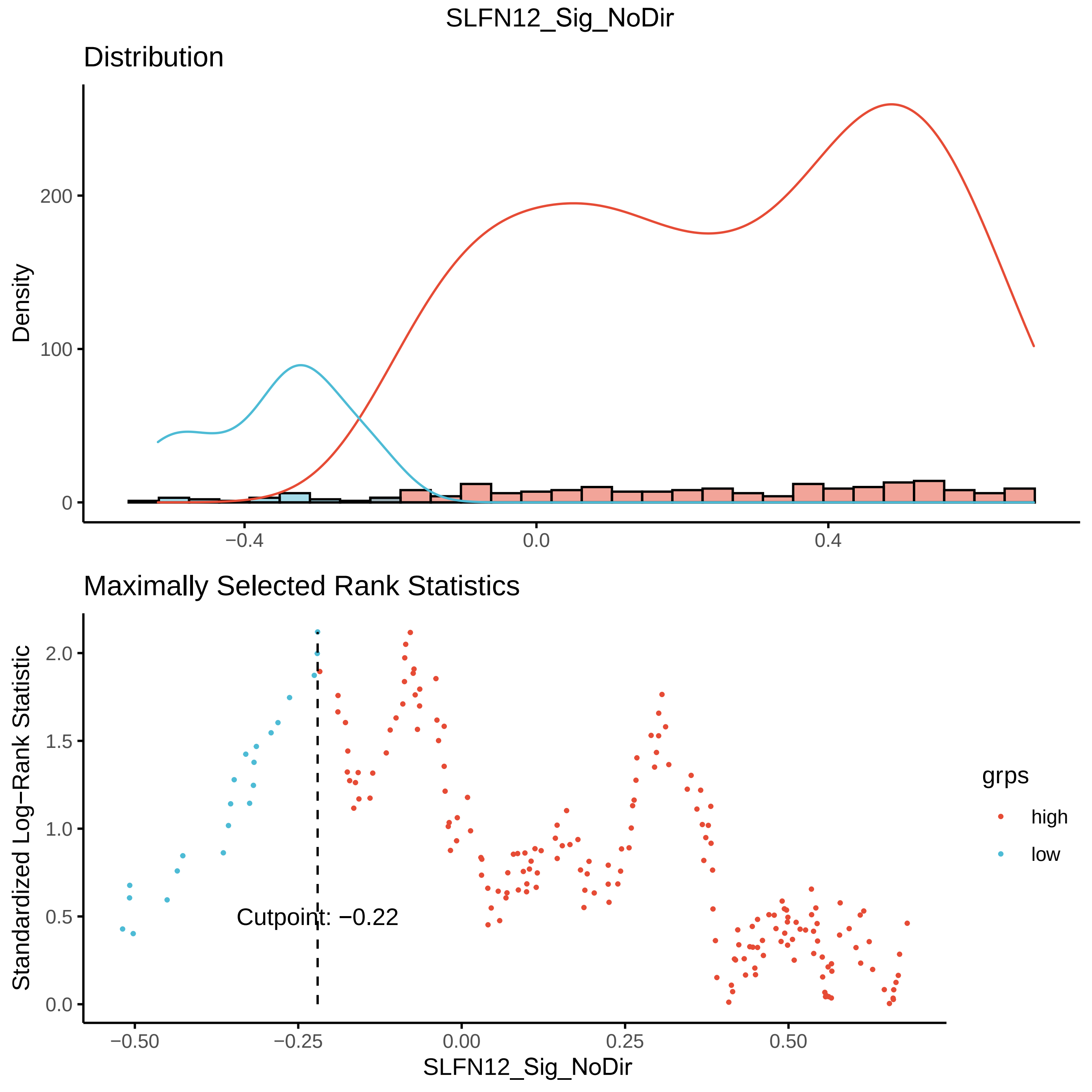

SLFN12_Sig_NoDir
Distribution
Density
200
100
0
−0.4
0.0
0.4
Maximally Selected Rank Statistics
Standardized Log−Rank Statistic
2.0
1.5
1.0
0.5
0.0
Cutpoint: −0.22
−0.50
−0.25
0.00
0.25
0.50
SLFN12_Sig_NoDir
grps
high
low

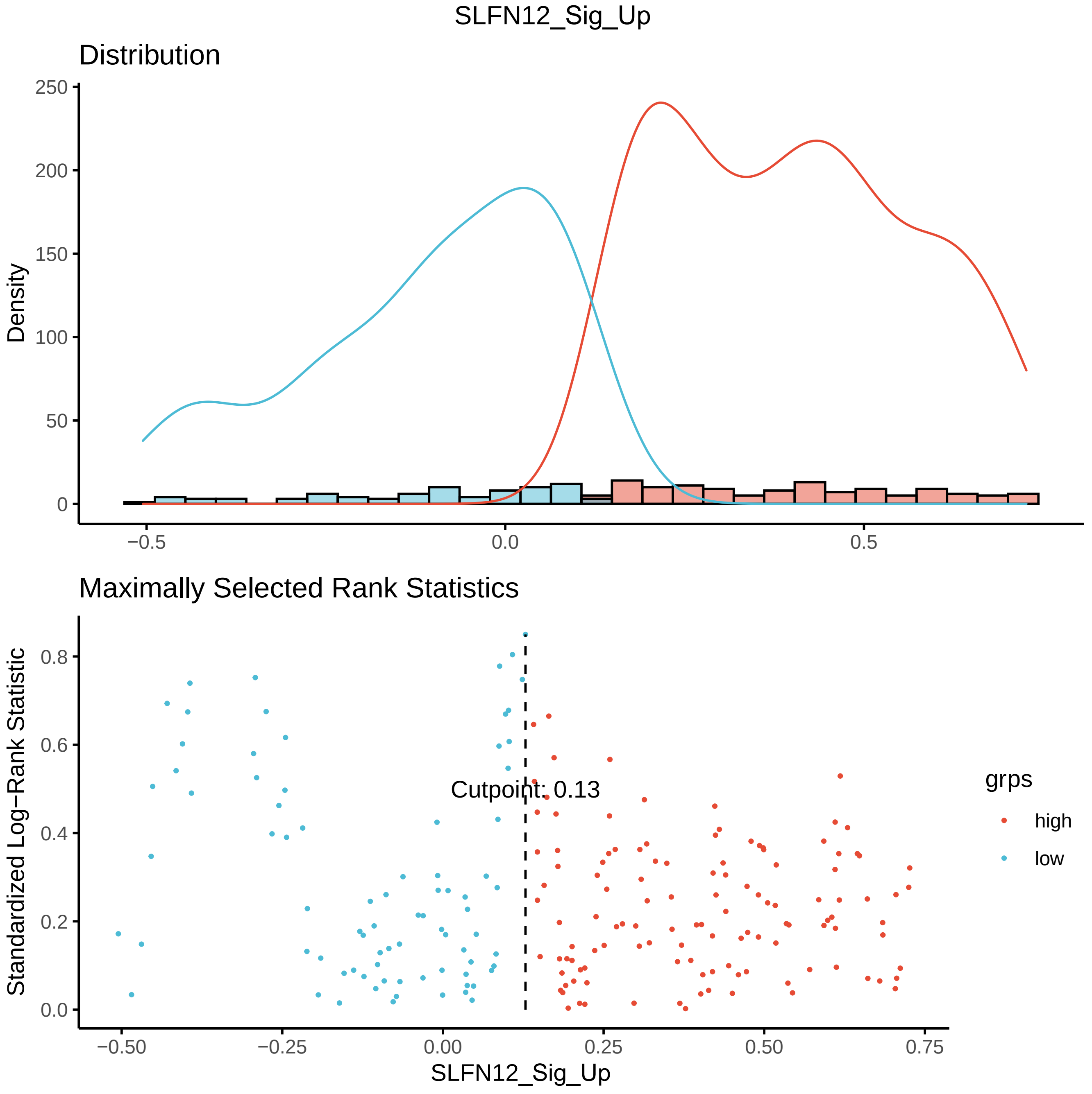

SLFN12_Sig_Up
Distribution
Density
250
200
150
100
50
0
−0.5
0.0
0.5
Maximally Selected Rank Statistics
Standardized Log−Rank Statistic
0.8
0.6
0.4
0.2
0.0
Cutpoint: 0.13
−0.50
−0.25
0.00
0.25
0.50
0.75
SLFN12_Sig_Up
grps
high
low

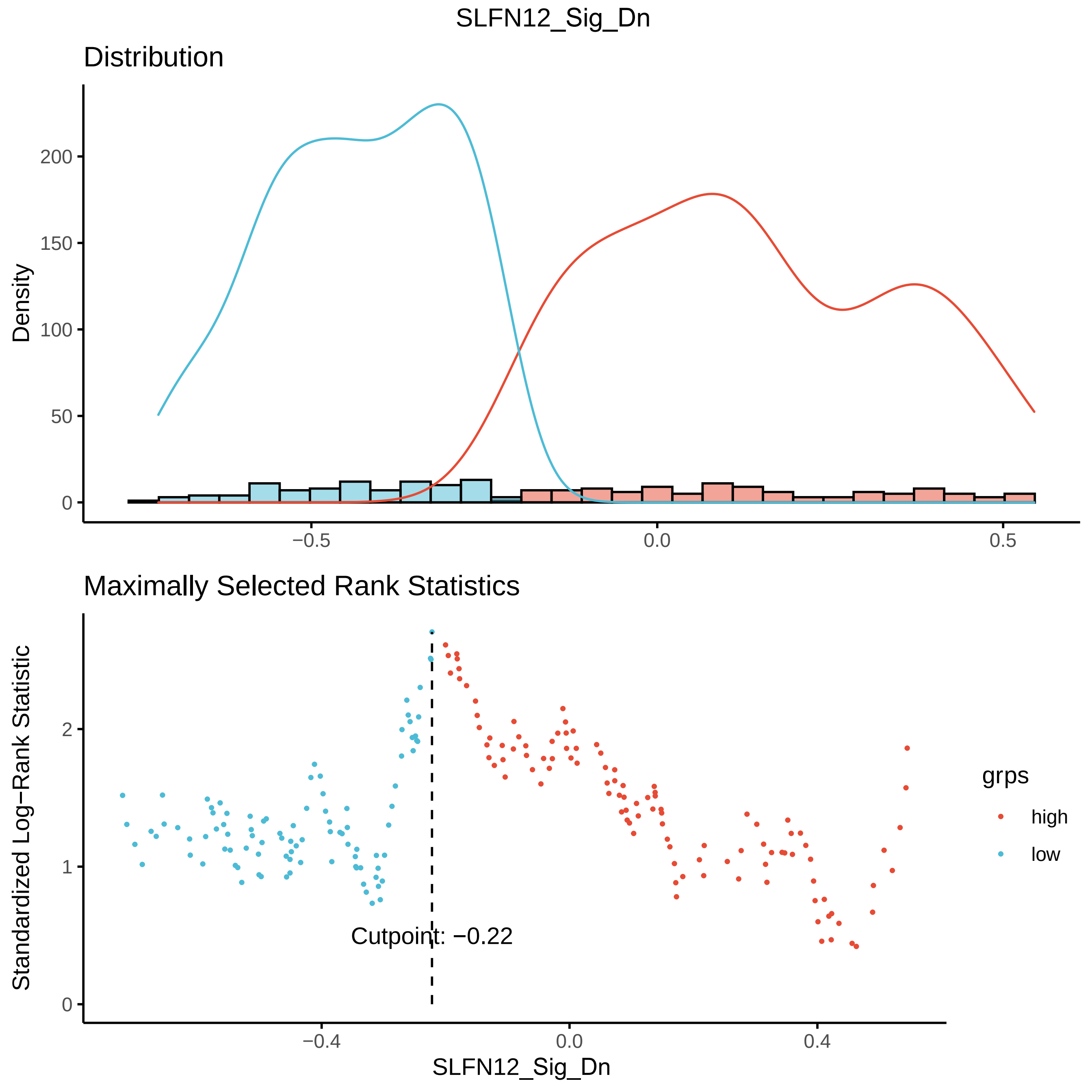
SLFN12_Sig_Dn
Distribution
Density
200
150
100
50
0
−0.5
0.0
0.5
Maximally Selected Rank Statistics
Standardized Log−Rank Statistic
2
1
0
Cutpoint: −0.22
−0.4
0.0
0.4
SLFN12_Sig_Dn
grps
high
low

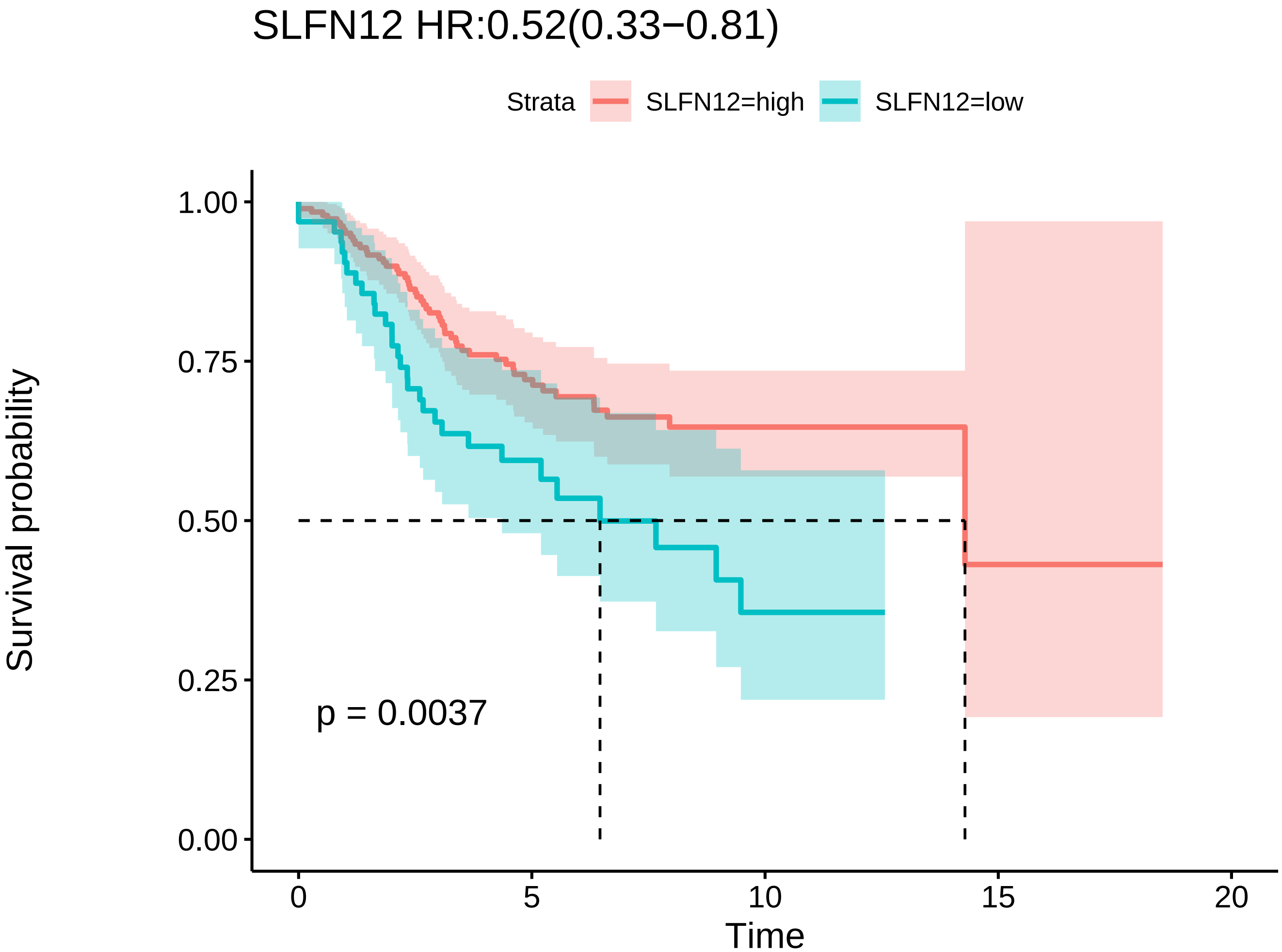

Number at risk

	0	5	10	15	20
SLFN12=high	188	83	16	2	0
SLFN12=low	64	22	6	0	0

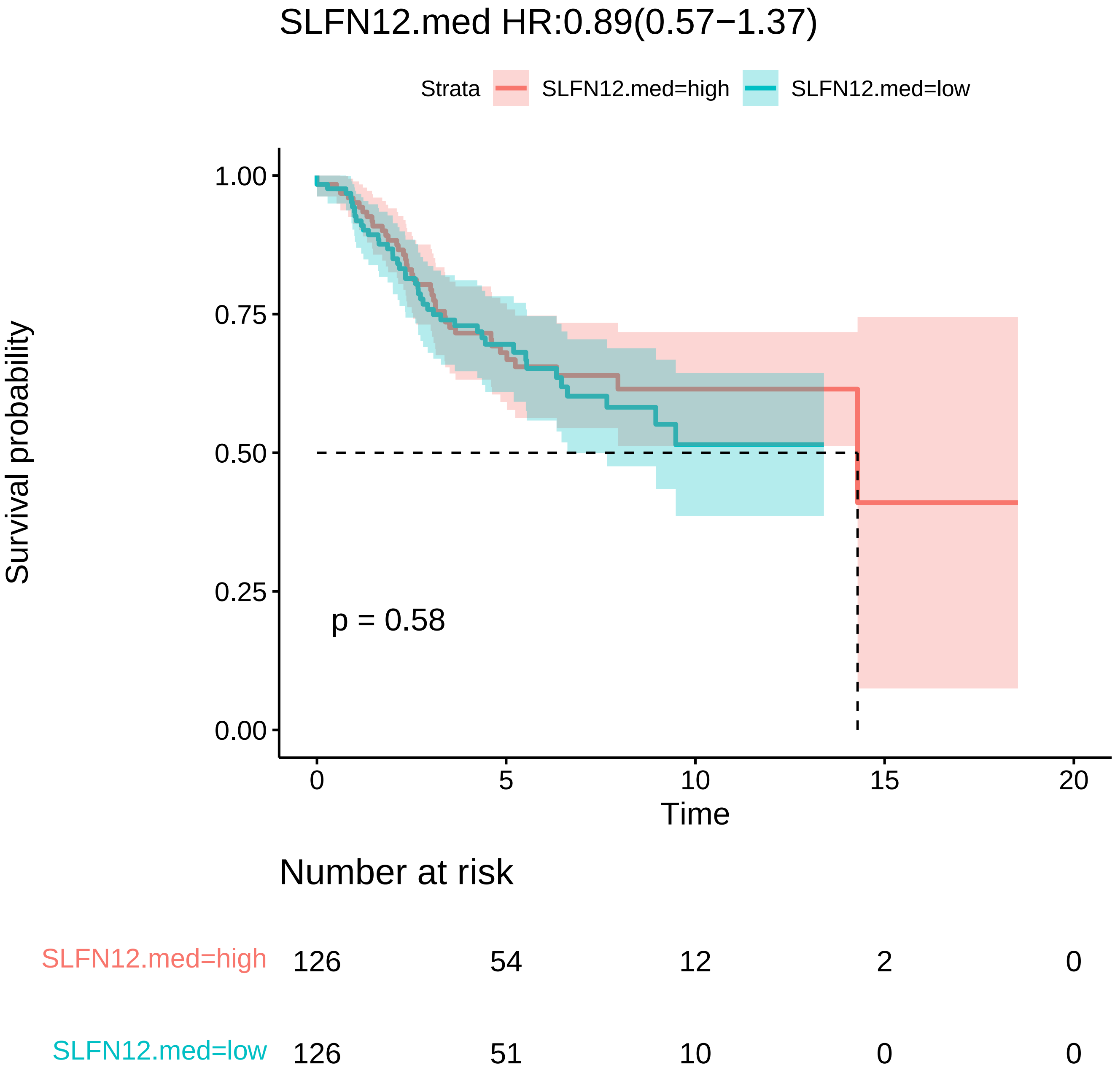
SLFN12.med HR:0.89(0.57−1.37)
Strata
SLFN12.med=high
SLFN12.med=low
Survival probability
1.00
0.75
0.50
0.25
0.00
p = 0.58
0
5
10
15
20
Time
Number at risk
SLFN12.med=high
126
54
12
2
0
SLFN12.med=low
126
51
10
0
0

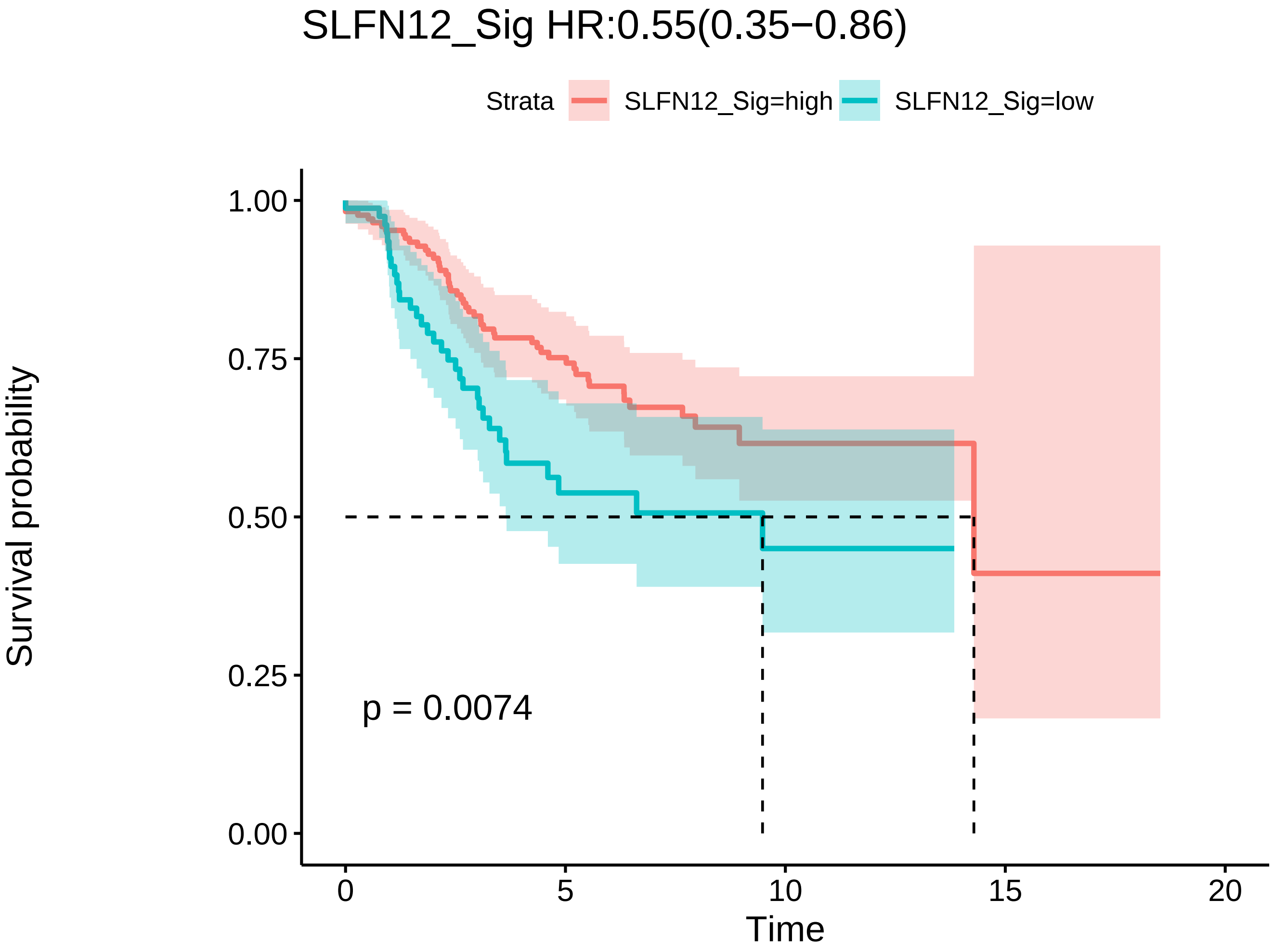

Number at risk

	0	5	10	15	20
SLFN12_Sig=high	171	87	15	2	0
SLFN12_Sig=low	81	18	7	0	0

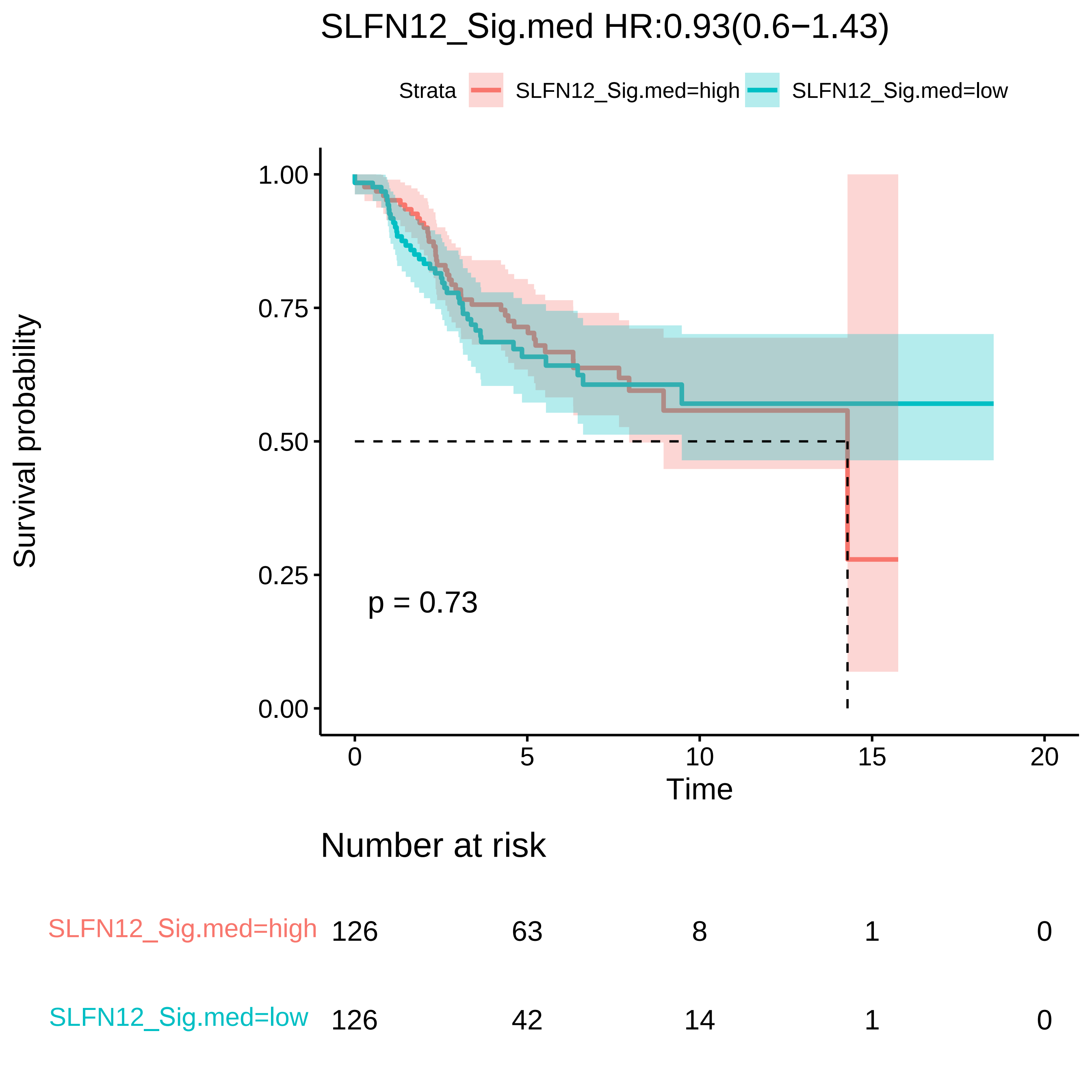

SLFN12_Sig.med HR:0.93(0.6−1.43)
Strata
SLFN12_Sig.med=high
SLFN12_Sig.med=low
Survival probability
1.00
0.75
0.50
0.25
0.00
p = 0.73
0
5
10
15
20
Time
Number at risk
SLFN12_Sig.med=high 126 63 8 1 0
SLFN12_Sig.med=low 126 42 14 1 0

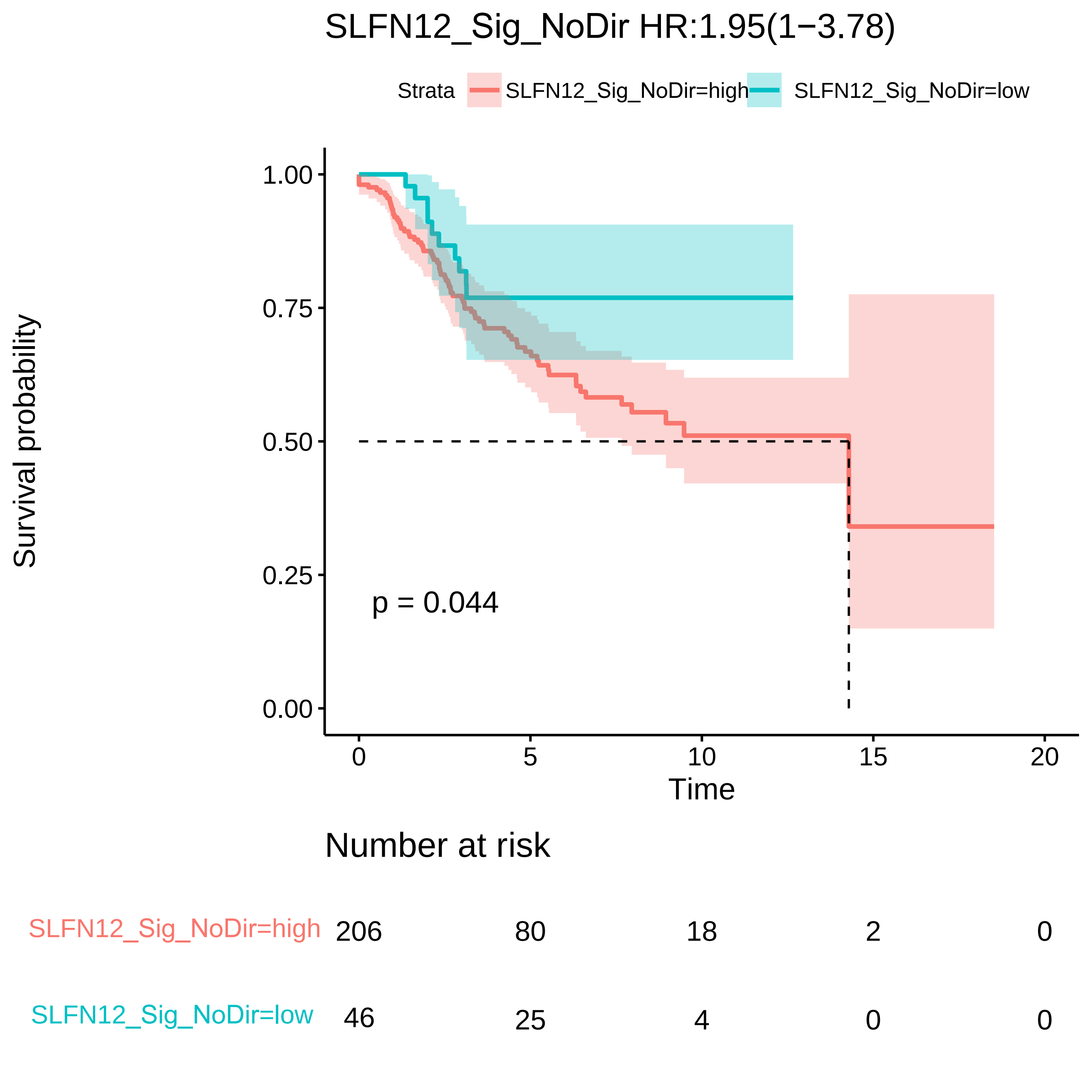
SLFN12_Sig_NoDir HR:1.95(1−3.78)
Strata
SLFN12_Sig_NoDir=high
SLFN12_Sig_NoDir=low
Survival probability
1.00
0.75
0.50
0.25
0.00
p = 0.044
0
5
10
15
20
Time
Number at risk
SLFN12_Sig_NoDir=high
206
80
18
2
0
SLFN12_Sig_NoDir=low
46
25
4
0
0

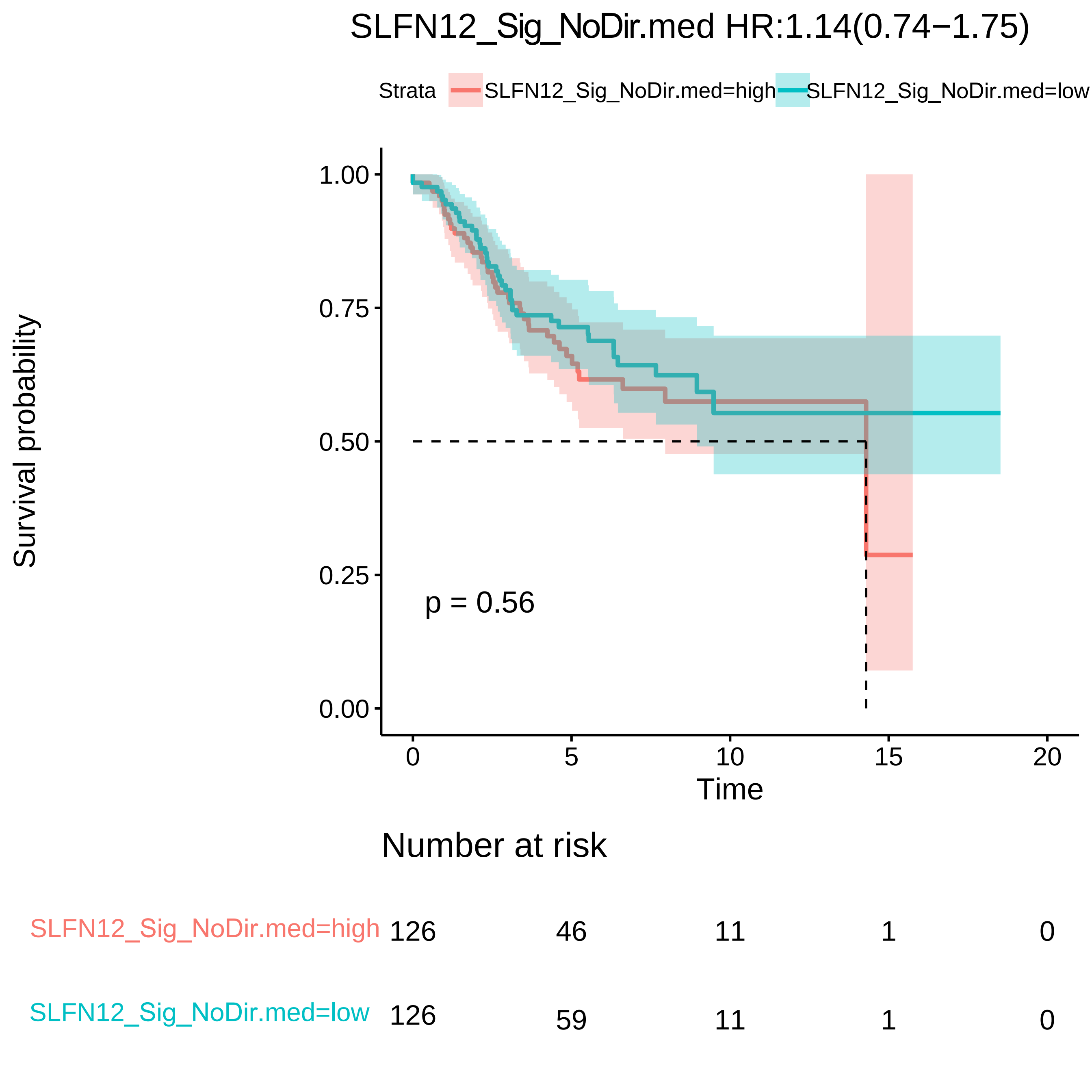
SLFN12_Sig_NoDir.med HR:1.14(0.74−1.75)
Strata
SLFN12_Sig_NoDir.med=high
SLFN12_Sig_NoDir.med=low
Survival probability
1.00
0.75
0.50
0.25
0.00
p = 0.56
0
5
10
15
20
Time
Number at risk
SLFN12_Sig_NoDir.med=high 126 46 11 1 0
SLFN12_Sig_NoDir.med=low 126 59 11 1 0

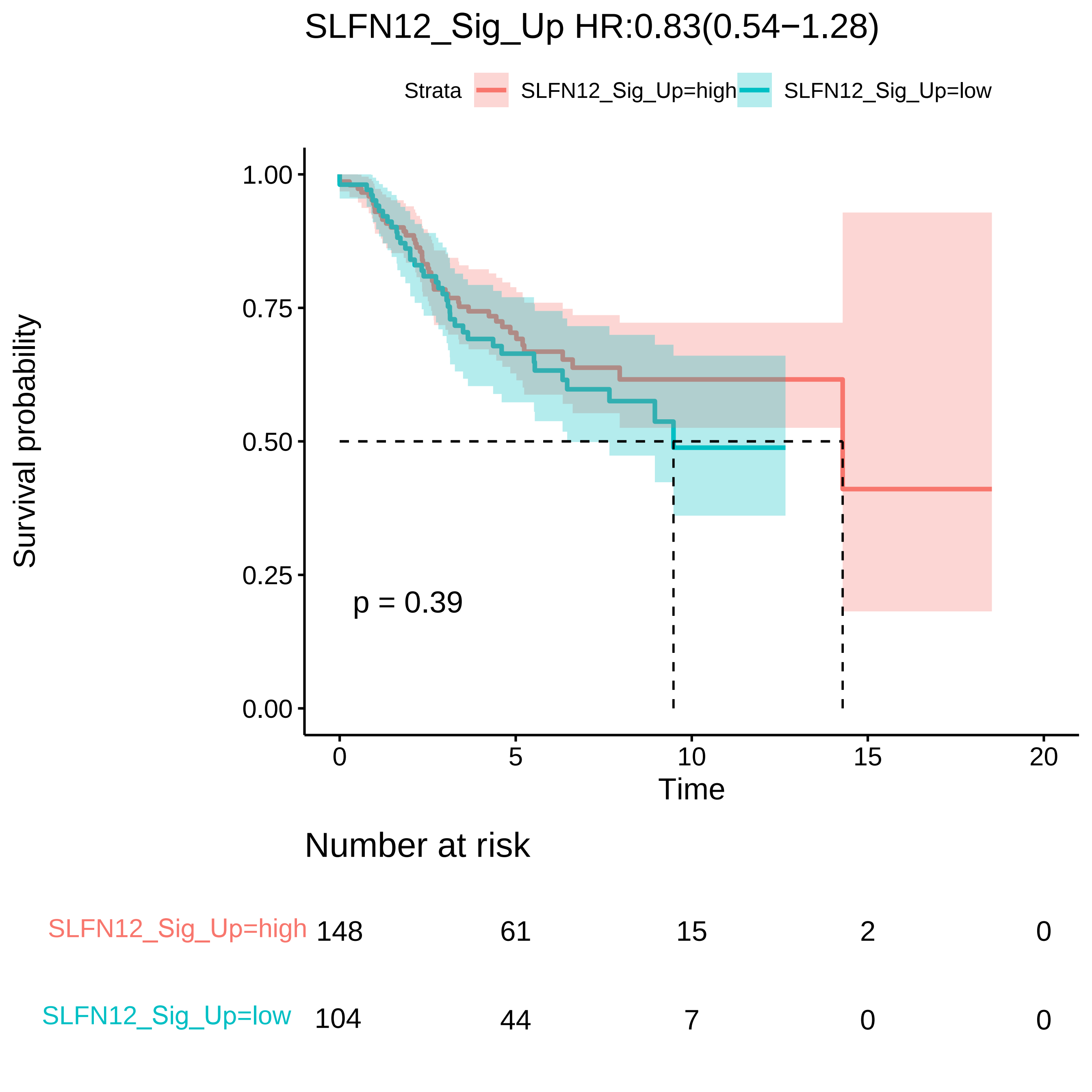
SLFN12_Sig_Up HR:0.83(0.54−1.28)
Strata
SLFN12_Sig_Up=high
SLFN12_Sig_Up=low
Survival probability
1.00
0.75
0.50
0.25
0.00
p = 0.39
0
5
10
15
20
Time
Number at risk
SLFN12_Sig_Up=high 148 61 15 2 0
SLFN12_Sig_Up=low 104 44 7 0 0

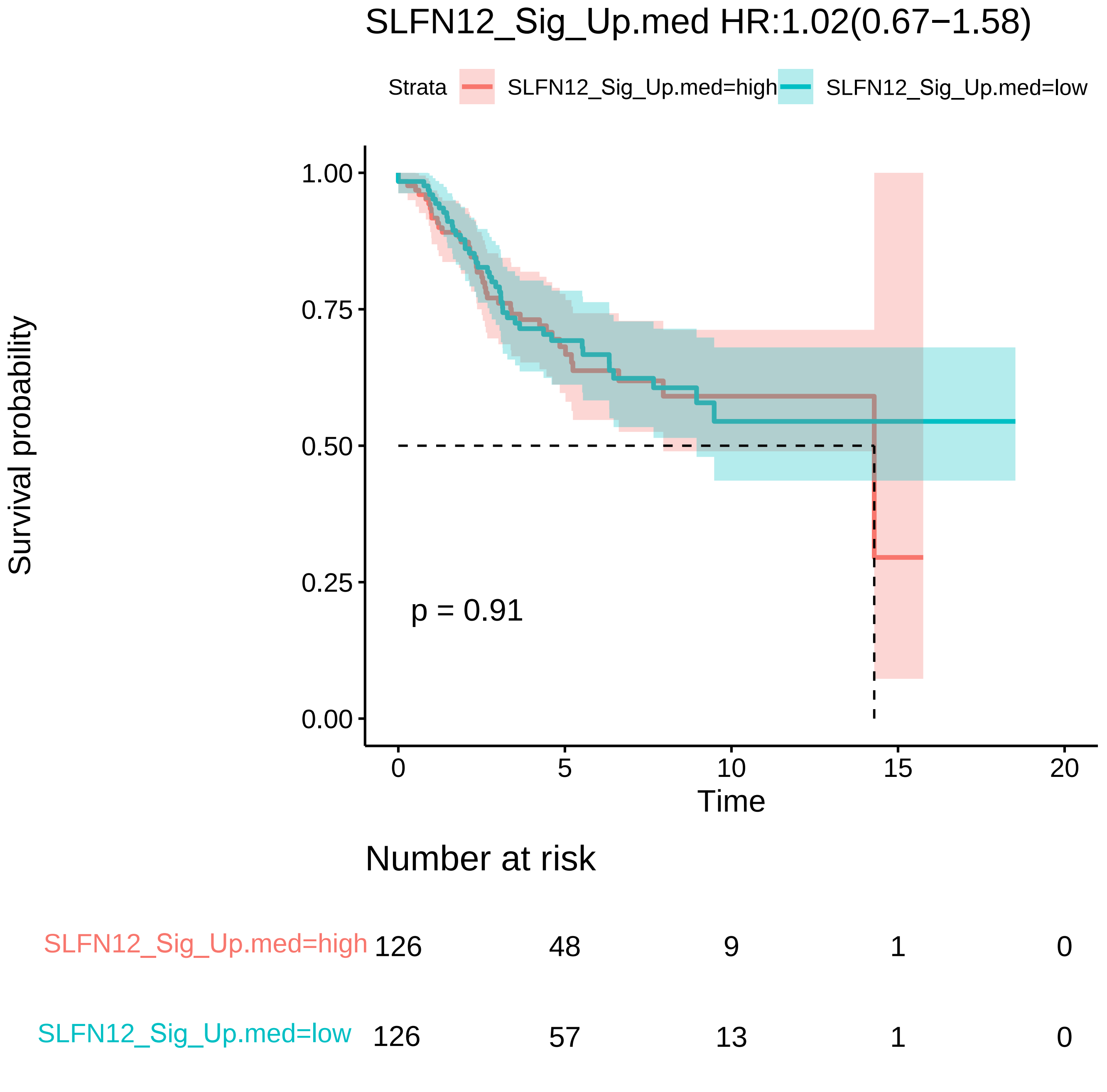

SLFN12_Sig_Up.med HR:1.02(0.67−1.58)
Strata
SLFN12_Sig_Up.med=high
SLFN12_Sig_Up.med=low
Survival probability
1.00
0.75
0.50
0.25
0.00
p = 0.91
0
5
10
15
20
Time
Number at risk
SLFN12_Sig_Up.med=high 126 48 9 1 0
SLFN12_Sig_Up.med=low 126 57 13 1 0

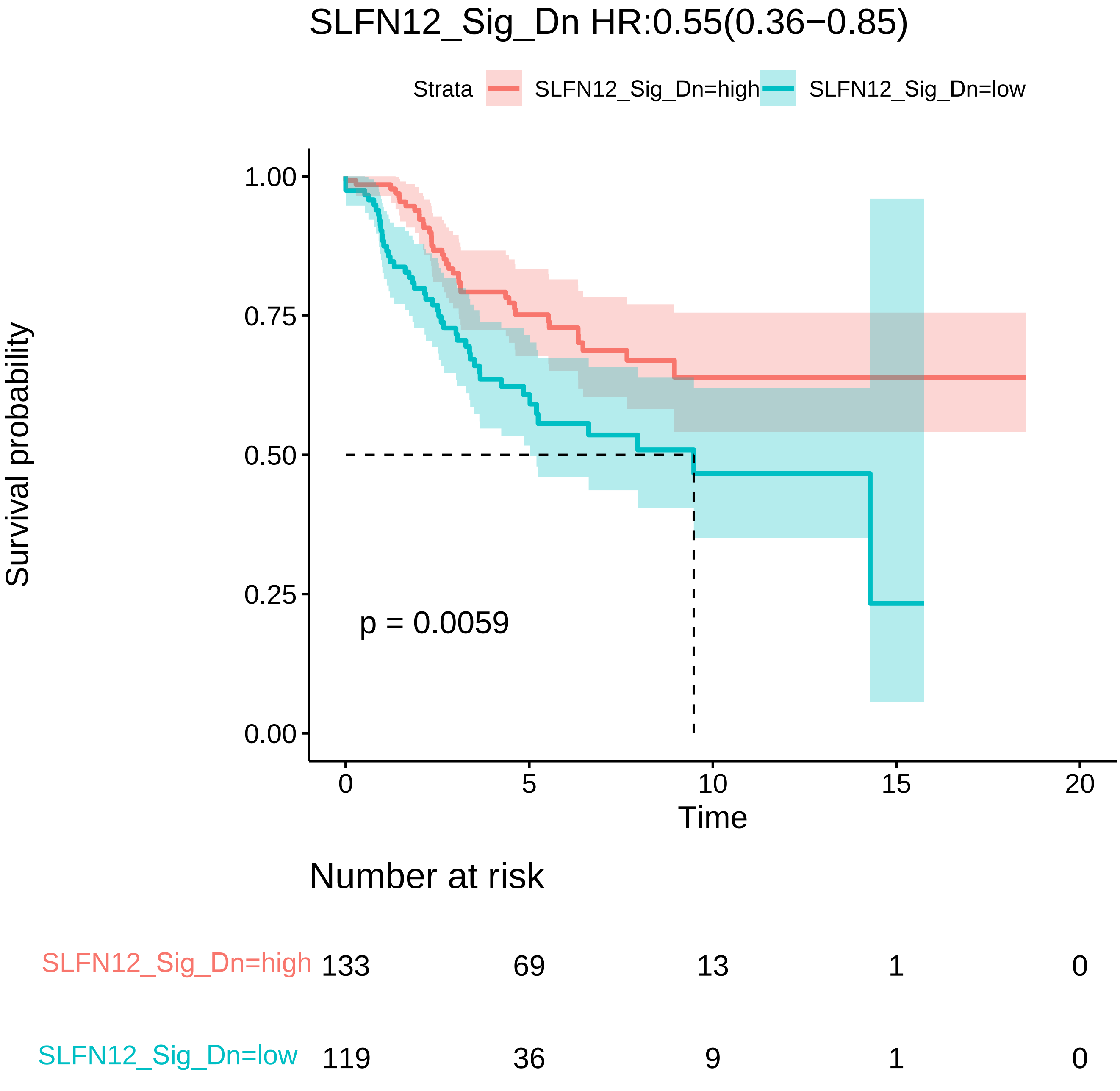
SLFN12_Sig_Dn HR:0.55(0.36−0.85)
Strata
SLFN12_Sig_Dn=high
SLFN12_Sig_Dn=low
Survival probability
1.00
0.75
0.50
0.25
0.00
p = 0.0059
0
5
10
15
20
Time
Number at risk
SLFN12_Sig_Dn=high 133 69 13 1 0
SLFN12_Sig_Dn=low 119 36 9 1 0

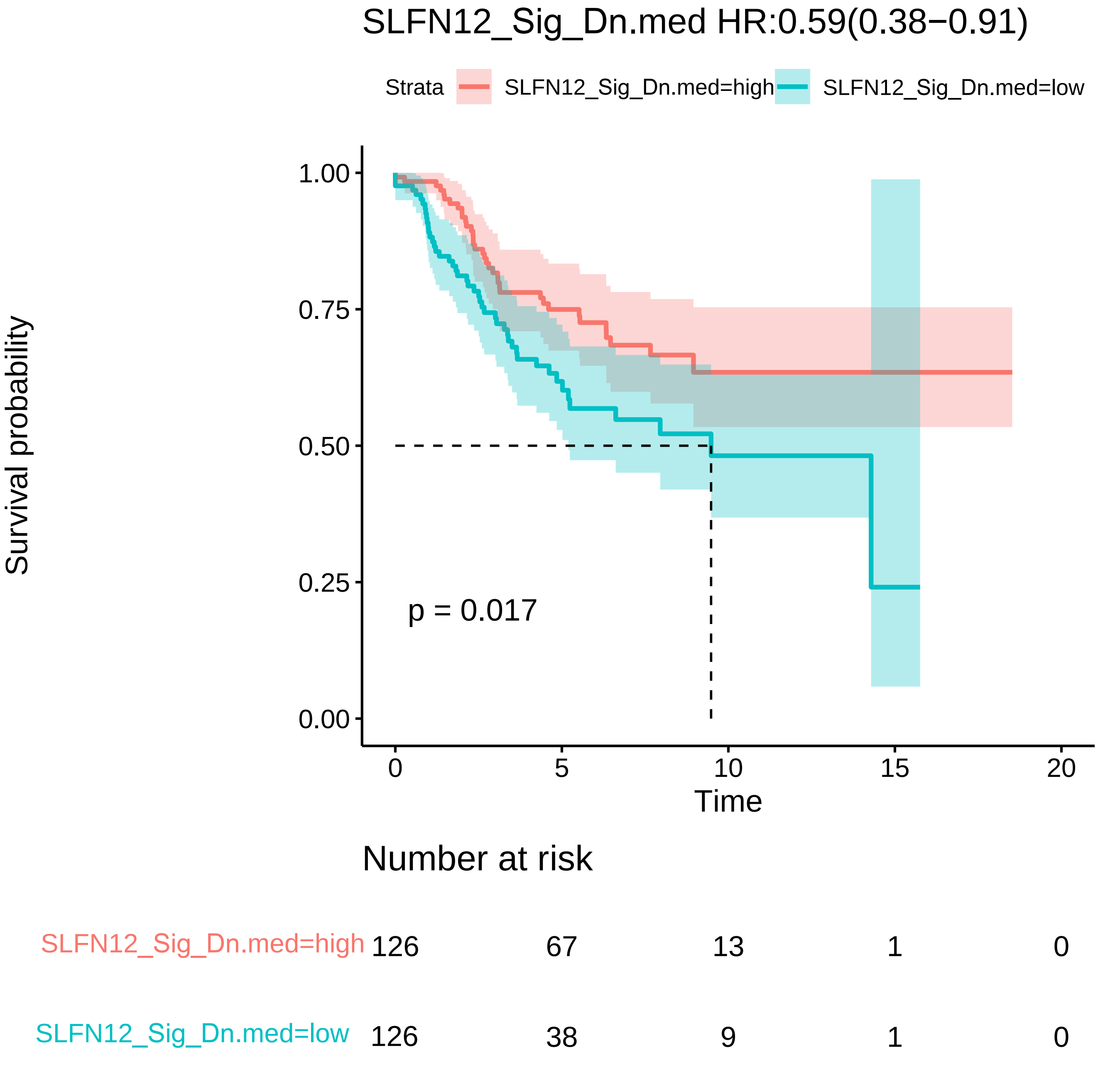

SLFN12_Sig_Dn.med HR:0.59(0.38−0.91)
Strata
SLFN12_Sig_Dn.med=high
SLFN12_Sig_Dn.med=low
Survival probability
1.00
0.75
0.50
0.25
0.00
p = 0.017
0
5
10
15
20
Time
Number at risk
SLFN12_Sig_Dn.med=high 126 67 13 1 0
SLFN12_Sig_Dn.med=low 126 38 9 1 0
