# Supplementary material for: Schlafen 12 Slows TNBC Tumor Growth, Induces Luminal Markers, and Predicts Favorable Survival
Source: Cancers (Basel). 2023 Jan 7;15(2):402. doi: 10.3390/cancers15020402 (PMC9856841; doi:10.3390/cancers15020402)
Supplement: Supplementary file 1 [file cancers-15-00402-s001.zip › Supplemental Figure 7 (1).pdf]

Supplementary Figure-7

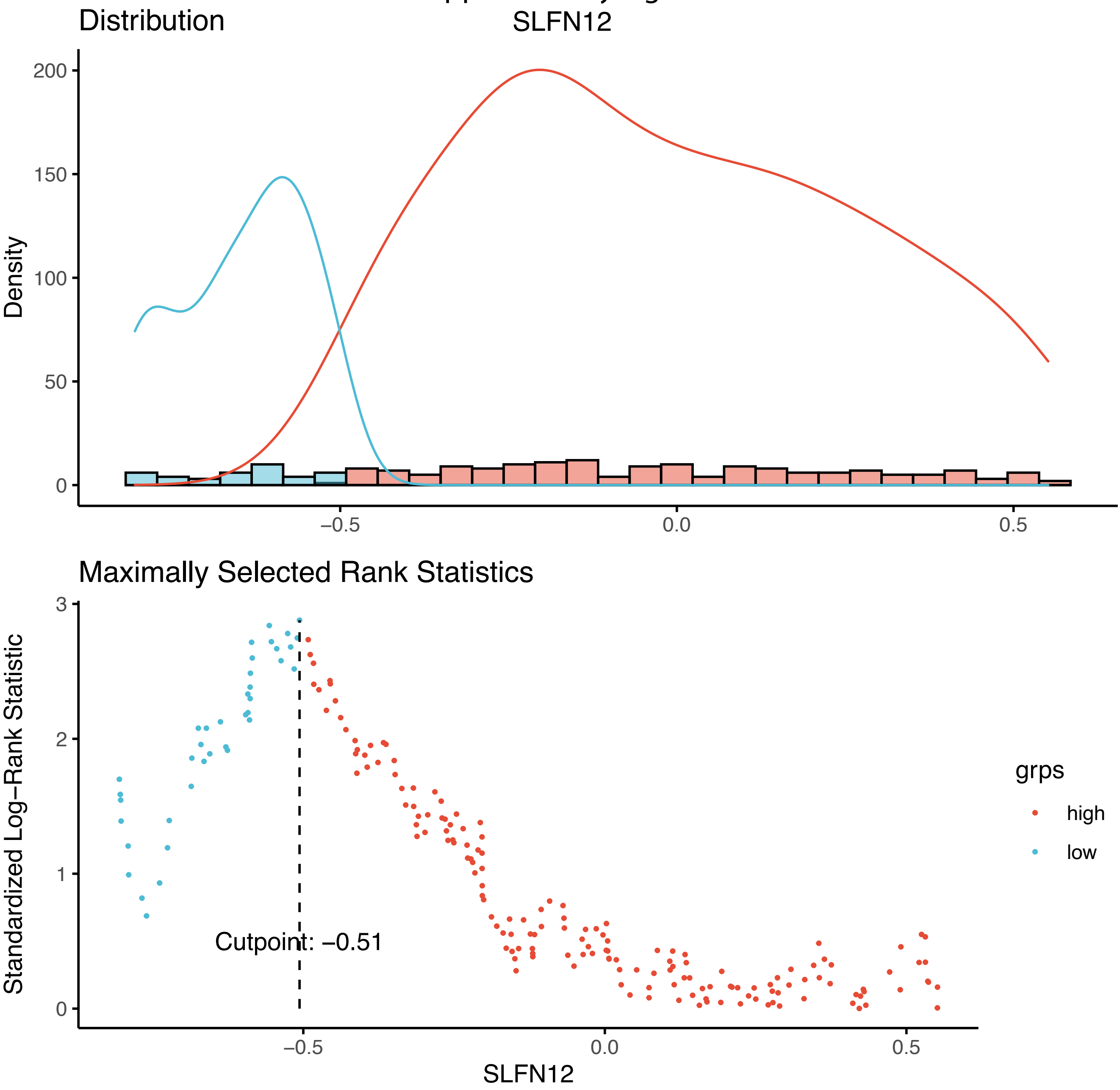

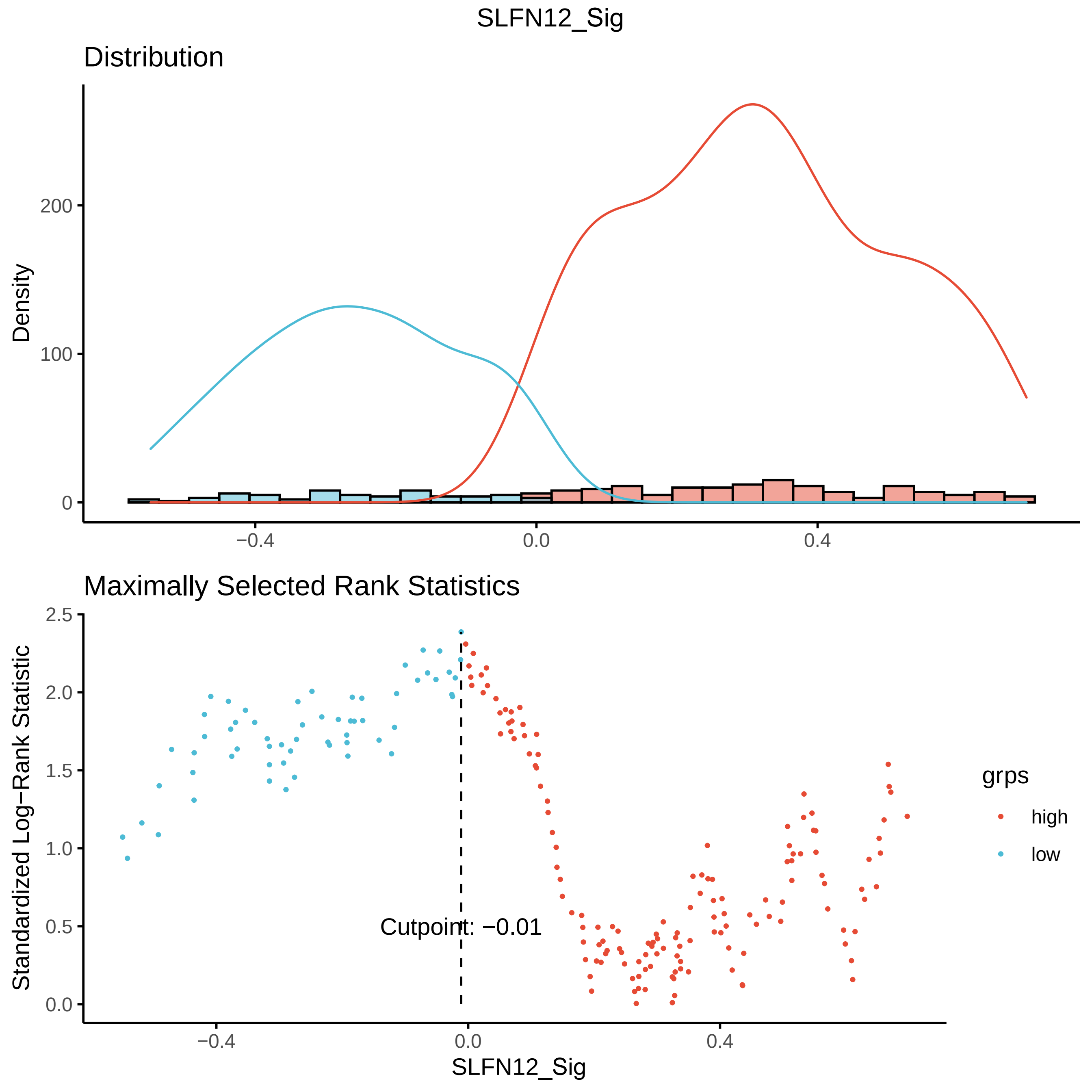

SLFN12_Sig
Distribution
Density
200
100
0
−0.4
0.0
0.4
Maximally Selected Rank Statistics
Standardized Log−Rank Statistic
2.5
2.0
1.5
1.0
0.5
0.0
Cutpoint: −0.01
−0.4
0.0
0.4
SLFN12_Sig
grps
high
low

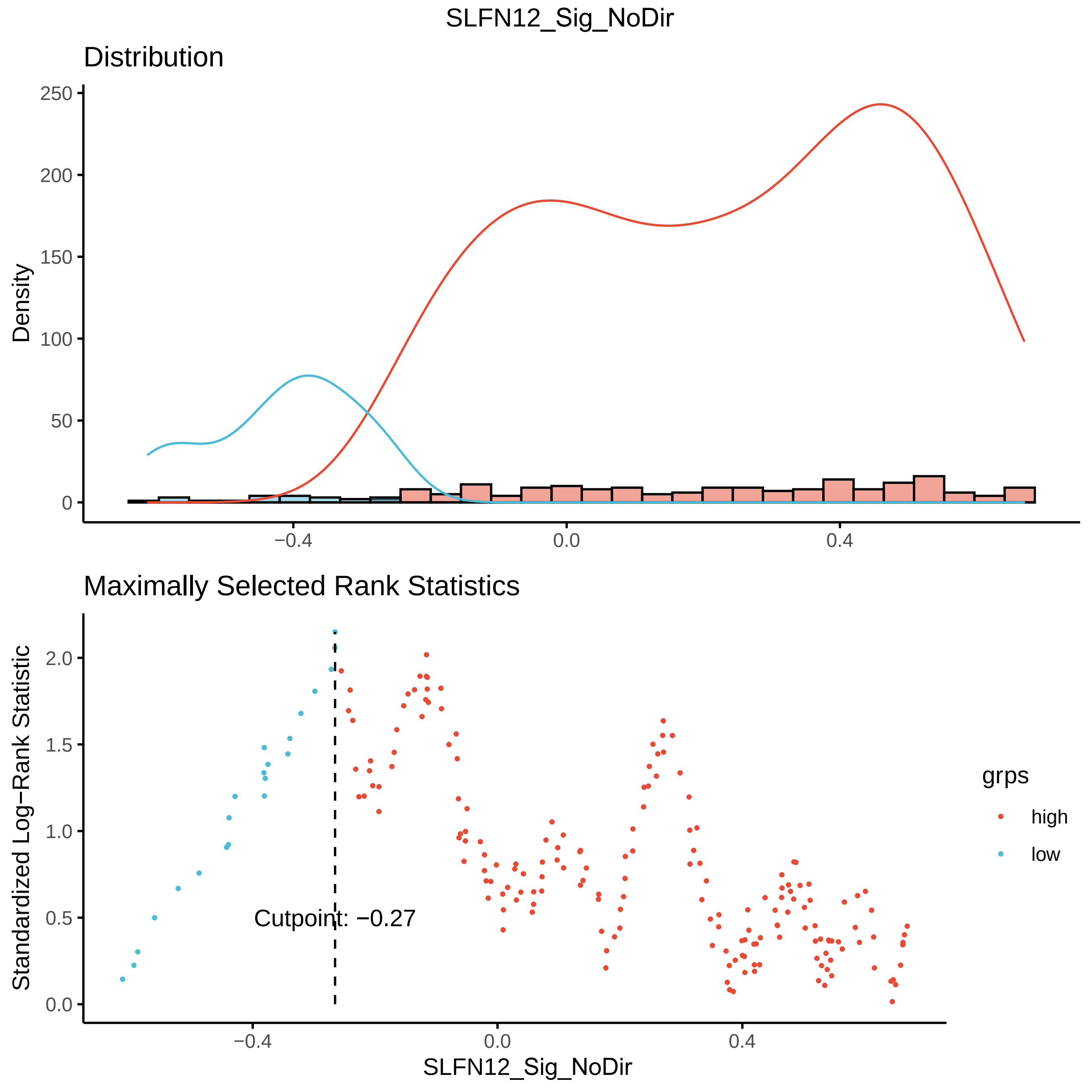
SLFN12_Sig_NoDir
Distribution
Density
250
200
150
100
50
0
−0.4
0.0
0.4
Maximally Selected Rank Statistics
Standardized Log−Rank Statistic
2.0
1.5
1.0
0.5
0.0
Cutpoint: −0.27
−0.4
0.0
0.4
SLFN12_Sig_NoDir
grps
high
low

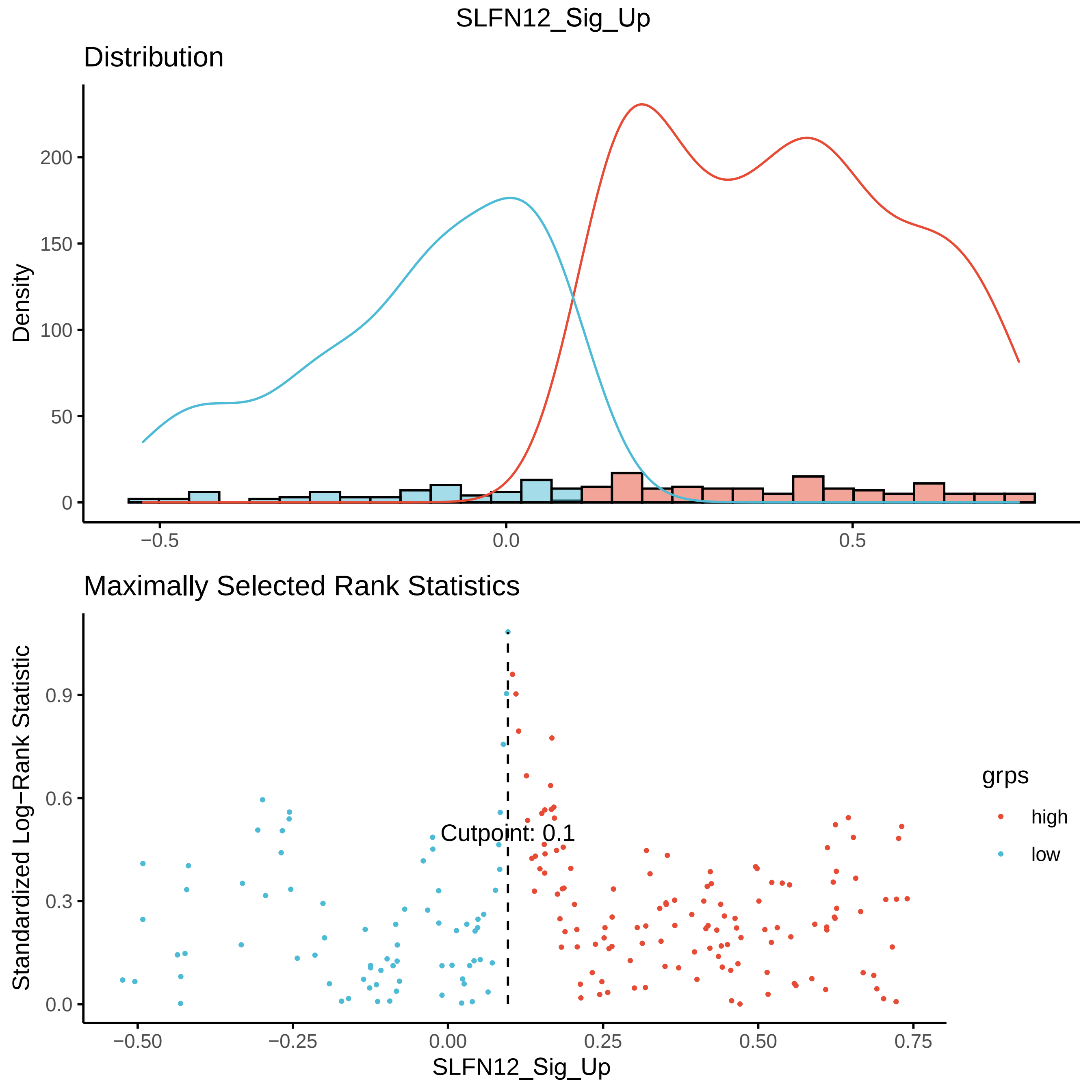

SLFN12_Sig_Up
Distribution
Density
200
150
100
50
0
−0.5
0.0
0.5
Maximally Selected Rank Statistics
Standardized Log−Rank Statistic
0.9
0.6
0.3
0.0
Cutpoint: 0.1
−0.50
−0.25
0.00
0.25
0.50
0.75
SLFN12_Sig_Up
grps
high
low

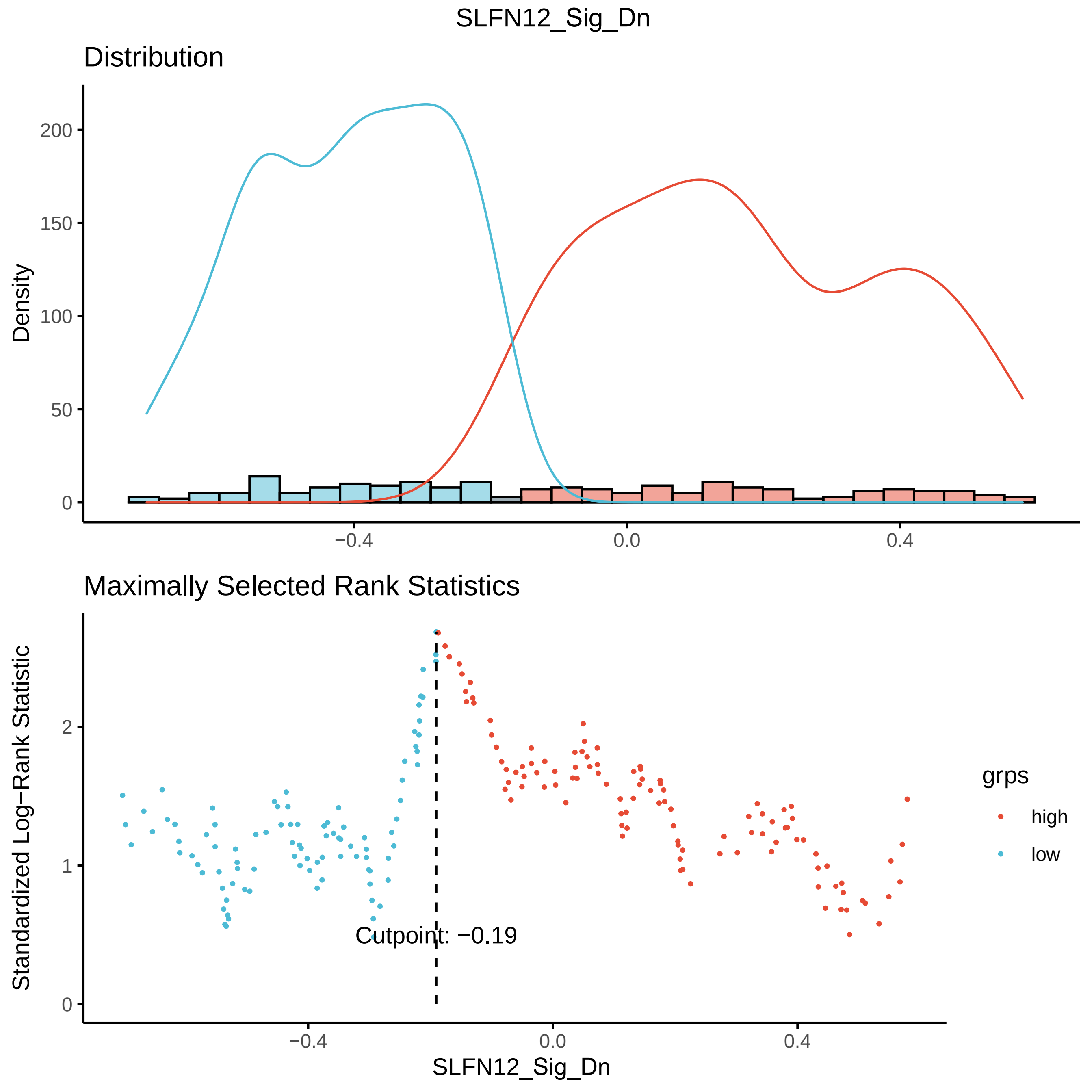

SLFN12_Sig_Dn
Distribution
Density
200
150
100
50
0
−0.4
0.0
0.4
Maximally Selected Rank Statistics
Standardized Log−Rank Statistic
2
1
0
Cutpoint: −0.19
−0.4
0.0
0.4
SLFN12_Sig_Dn
grps
high
low

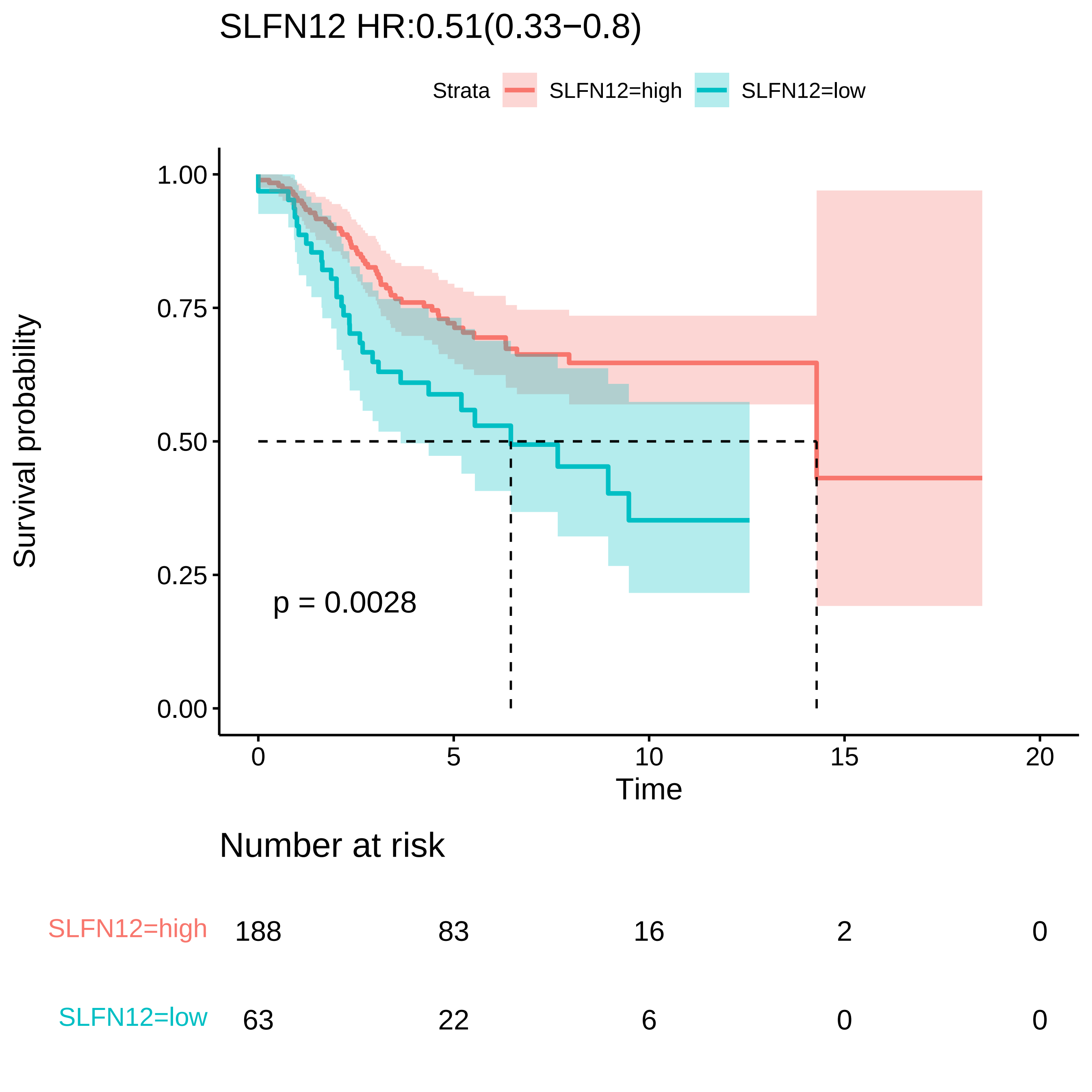
SLFN12 HR:0.51(0.33−0.8)
Strata
SLFN12=high
SLFN12=low
1.00
0.75
0.50
0.25
0.00
Survival probability
p = 0.0028
0
5
10
15
20
Time
Number at risk
SLFN12=high 188 83 16 2 0
SLFN12=low 63 22 6 0 0

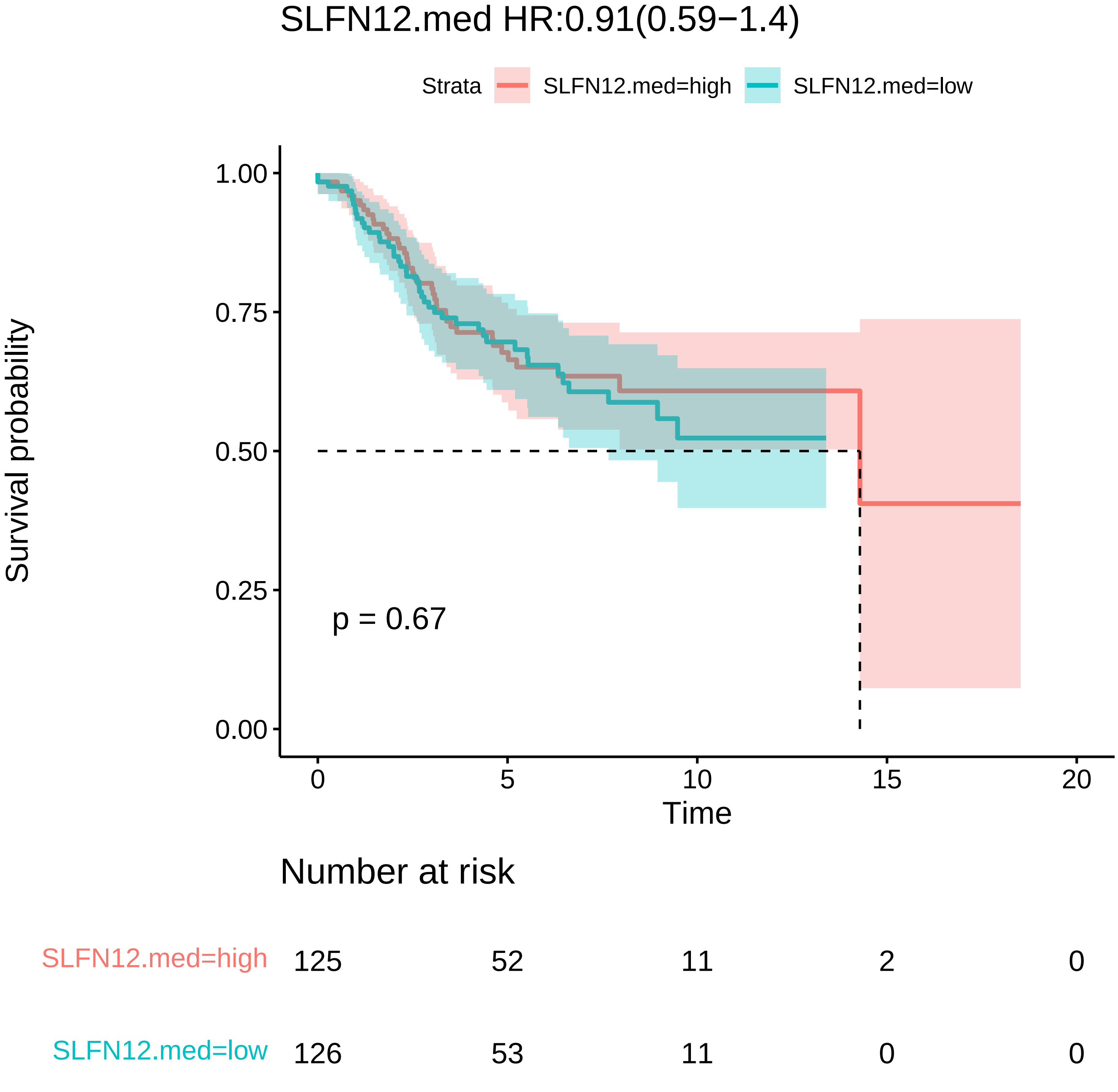

SLFN12.med HR:0.91(0.59−1.4)
Strata
SLFN12.med=high
SLFN12.med=low
Survival probability
1.00
0.75
0.50
0.25
0.00
p = 0.67
0
5
10
15
20
Time
Number at risk
SLFN12.med=high
125
52
11
2
0
SLFN12.med=low
126
53
11
0
0

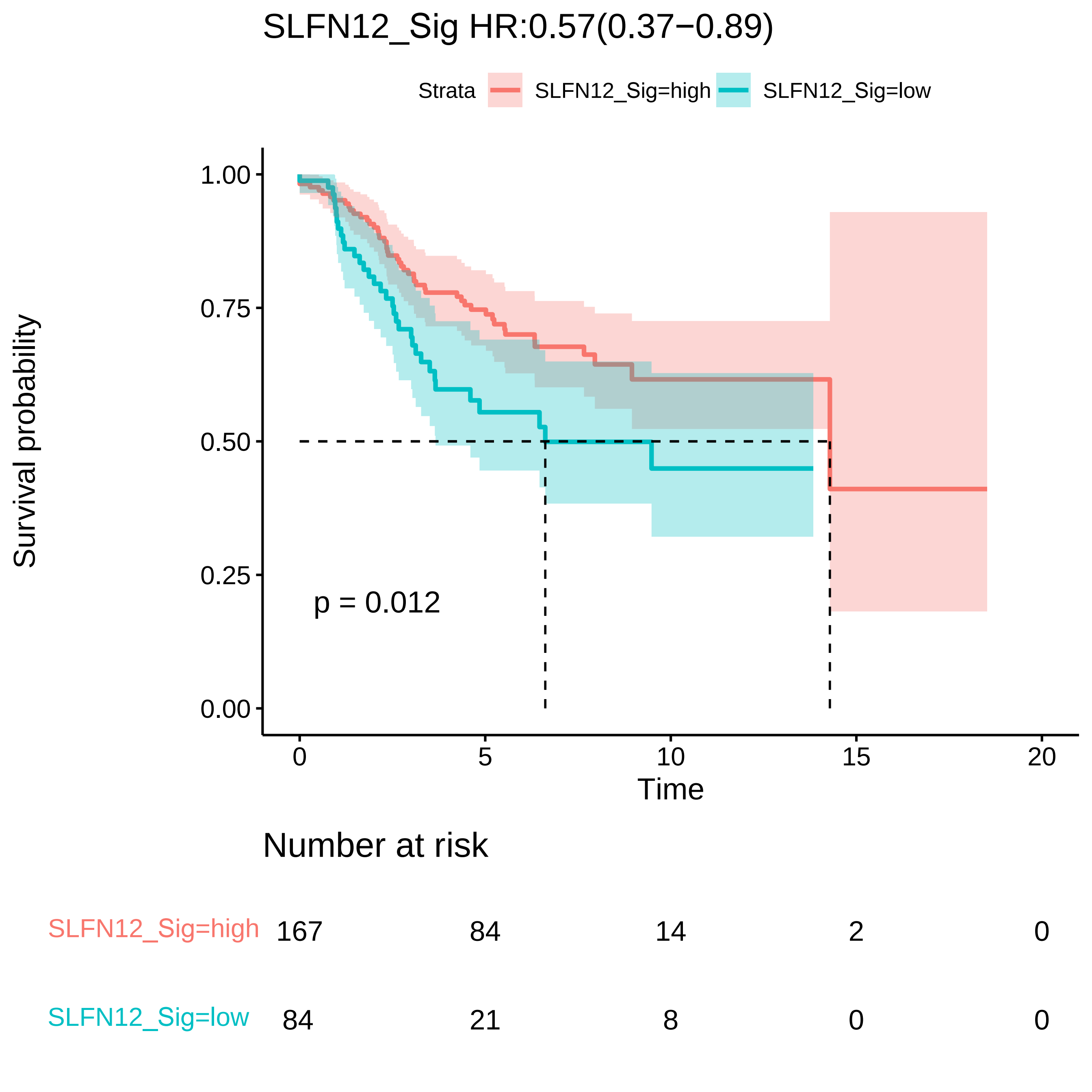

SLFN12_Sig HR:0.57(0.37−0.89)
Strata
SLFN12_Sig=high
SLFN12_Sig=low
Survival probability
1.00
0.75
0.50
0.25
0.00
p = 0.012
0
5
10
15
20
Time
Number at risk
SLFN12_Sig=high 167 84 14 2 0
SLFN12_Sig=low 84 21 8 0 0

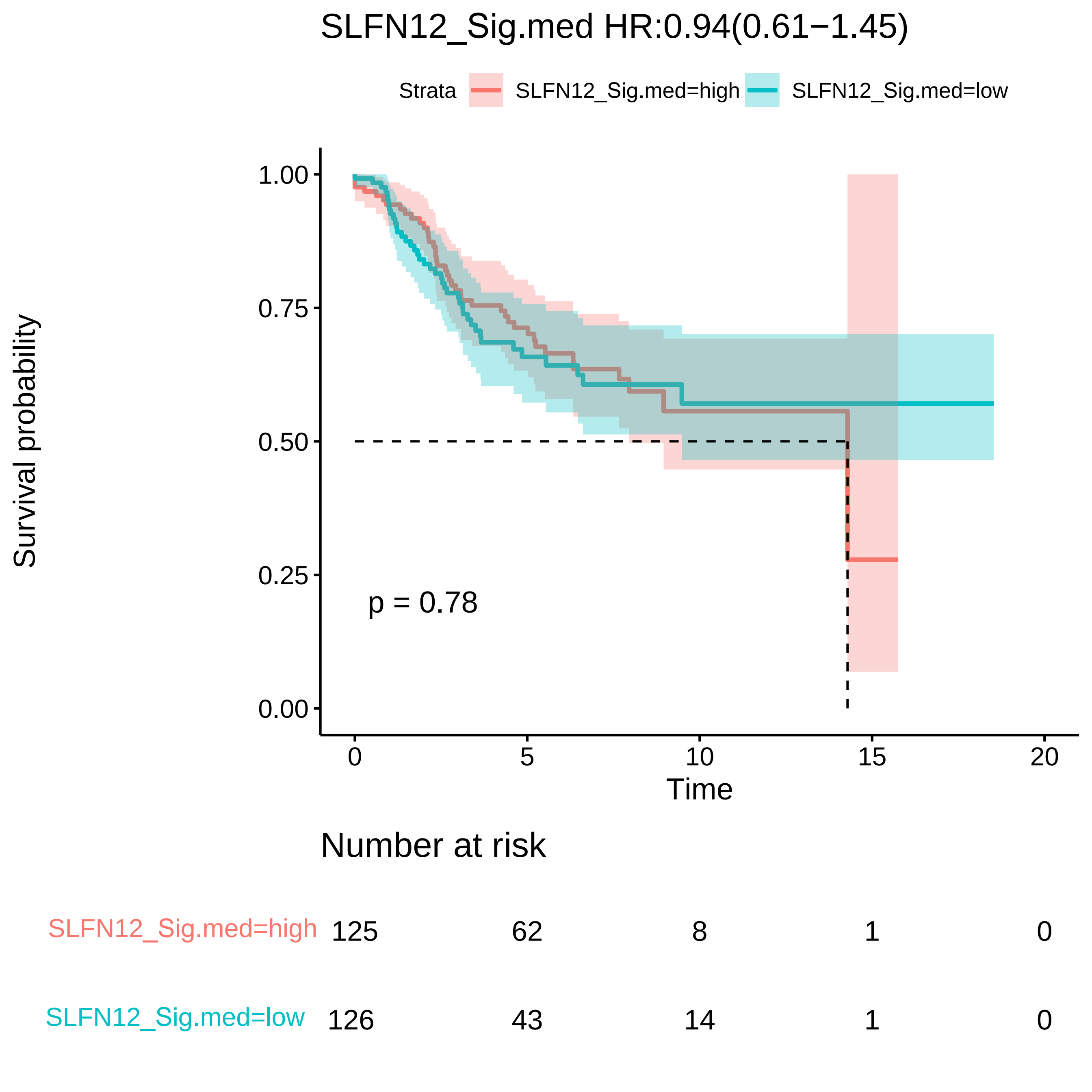

SLFN12_Sig.med HR:0.94(0.61−1.45)
Strata
SLFN12_Sig.med=high
SLFN12_Sig.med=low
Survival probability
1.00
0.75
0.50
0.25
0.00
p = 0.78
0
5
10
15
20
Time
Number at risk
SLFN12_Sig.med=high
125
62
8
1
0
SLFN12_Sig.med=low
126
43
14
1
0

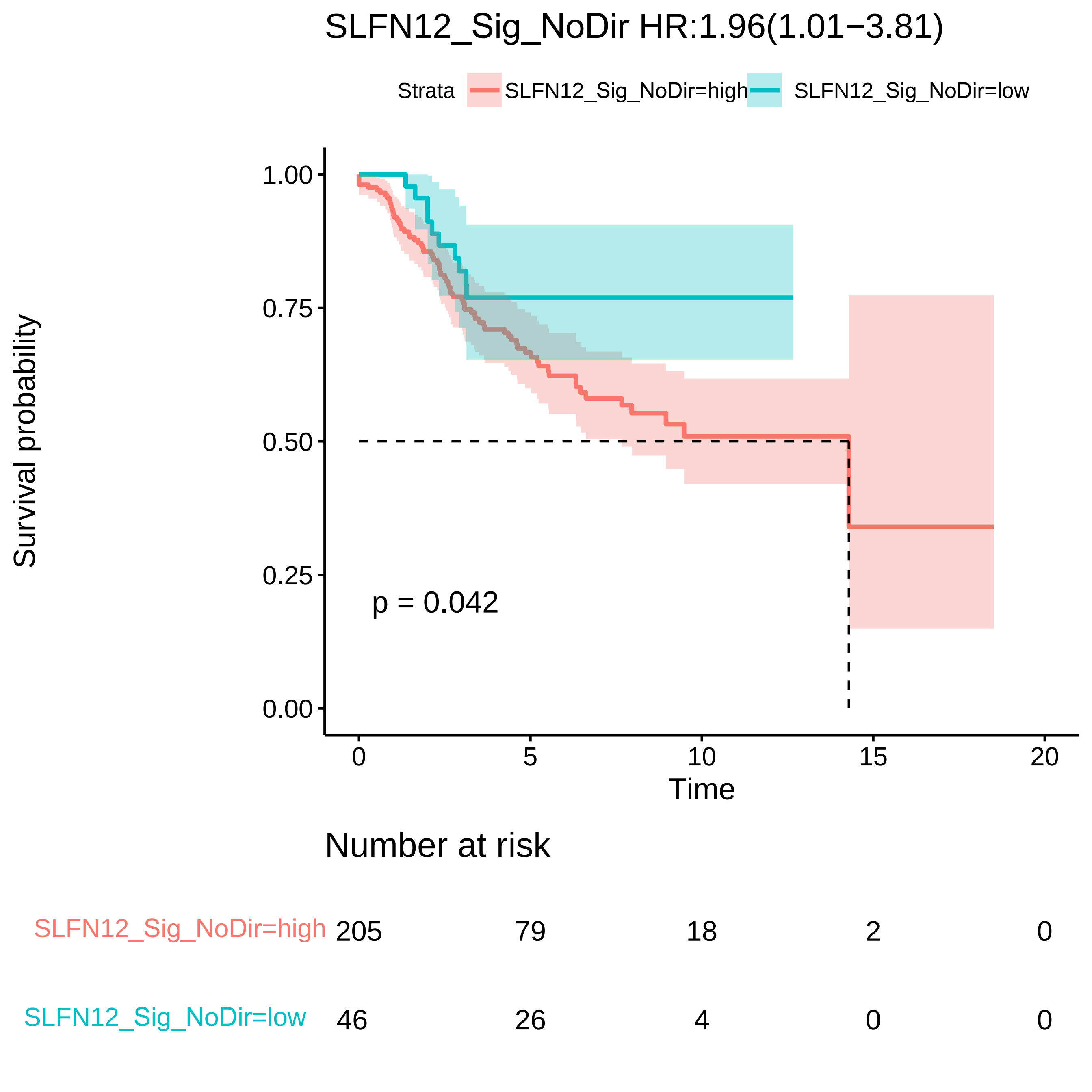
SLFN12_Sig_NoDir HR:1.96(1.01−3.81)
Strata
SLFN12_Sig_NoDir=high
SLFN12_Sig_NoDir=low
Survival probability
1.00
0.75
0.50
0.25
0.00
p = 0.042
0
5
10
15
20
Time
Number at risk
SLFN12_Sig_NoDir=high 205 79 18 2 0
SLFN12_Sig_NoDir=low 46 26 4 0 0

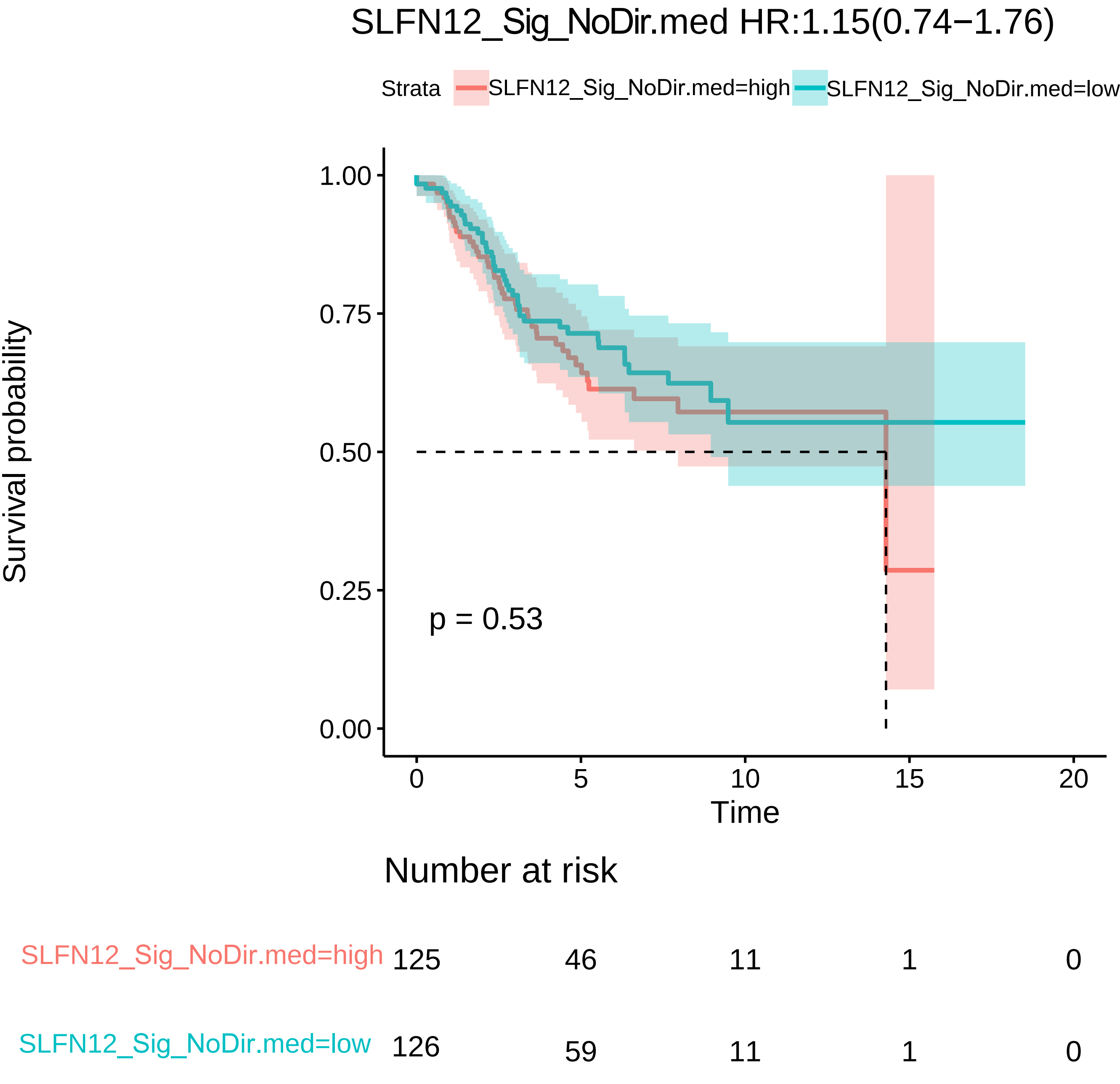

SLFN12_Sig_NoDir.med HR:1.15(0.74−1.76)
Strata
SLFN12_Sig_NoDir.med=high
SLFN12_Sig_NoDir.med=low
Survival probability
1.00
0.75
0.50
0.25
0.00
p = 0.53
0
5
10
15
20
Time
Number at risk
SLFN12_Sig_NoDir.med=high 125 46 11 1 0
SLFN12_Sig_NoDir.med=low 126 59 11 1 0

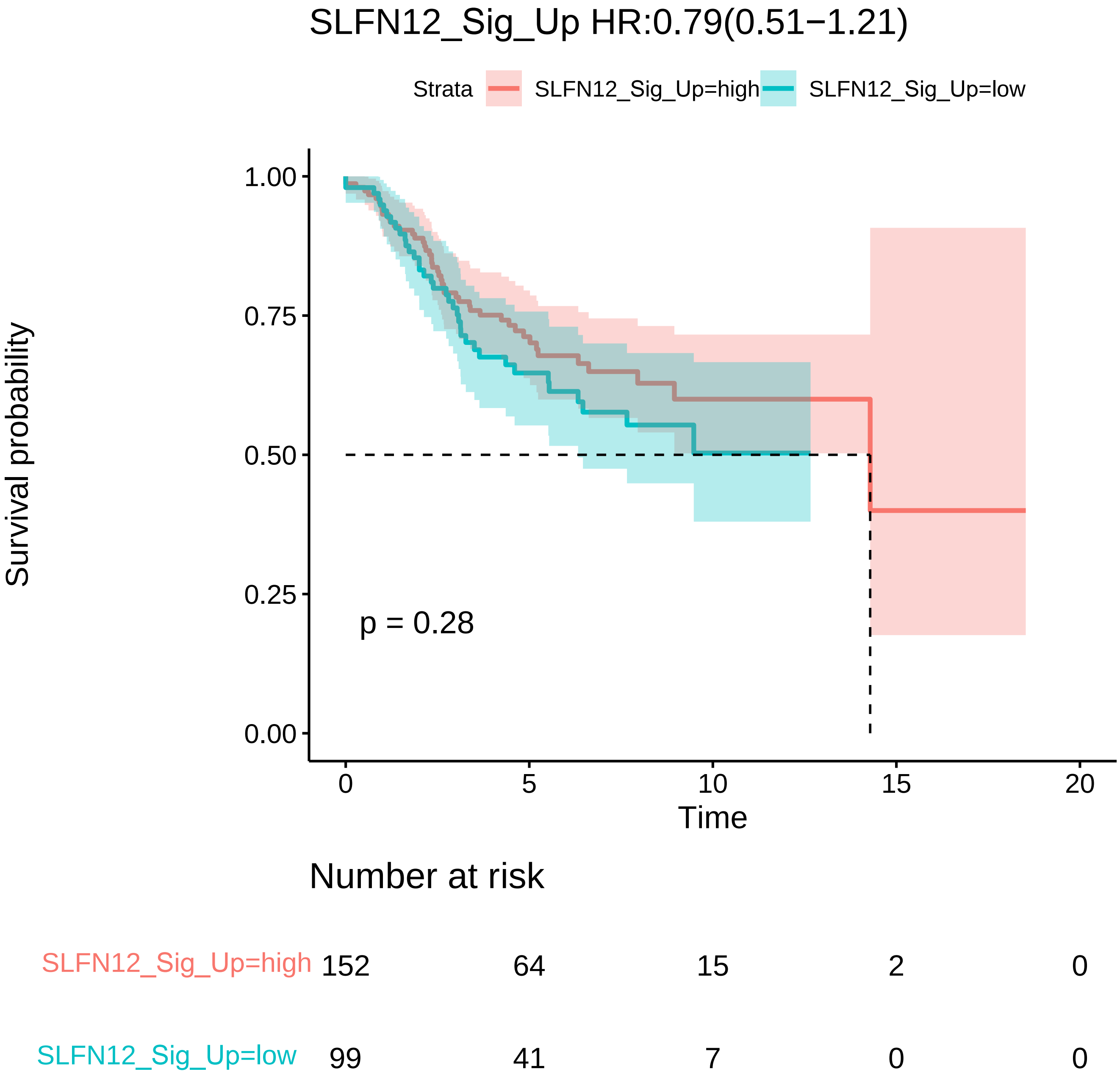
SLFN12_Sig_Up HR:0.79(0.51−1.21)
Strata
SLFN12_Sig_Up=high
SLFN12_Sig_Up=low
Survival probability
1.00
0.75
0.50
0.25
0.00
p = 0.28
0
5
10
15
20
Time
Number at risk
SLFN12_Sig_Up=high 152 64 15 2 0
SLFN12_Sig_Up=low 99 41 7 0 0

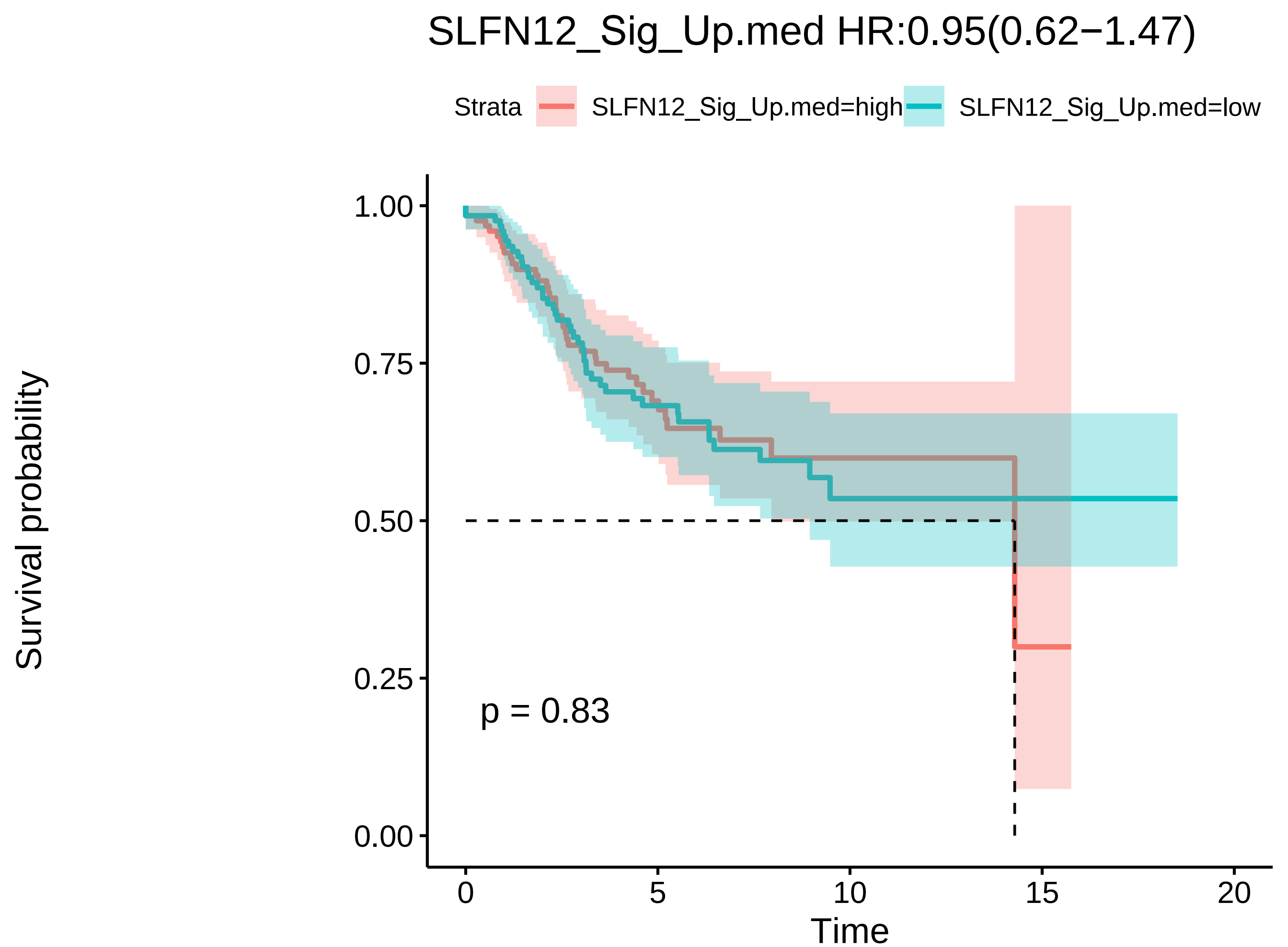

Number at risk

	0	5	10	15	20
SLFN12_Sig_Up.med=high	125	49	9	1	0
SLFN12_Sig_Up.med=low	126	56	13	1	0

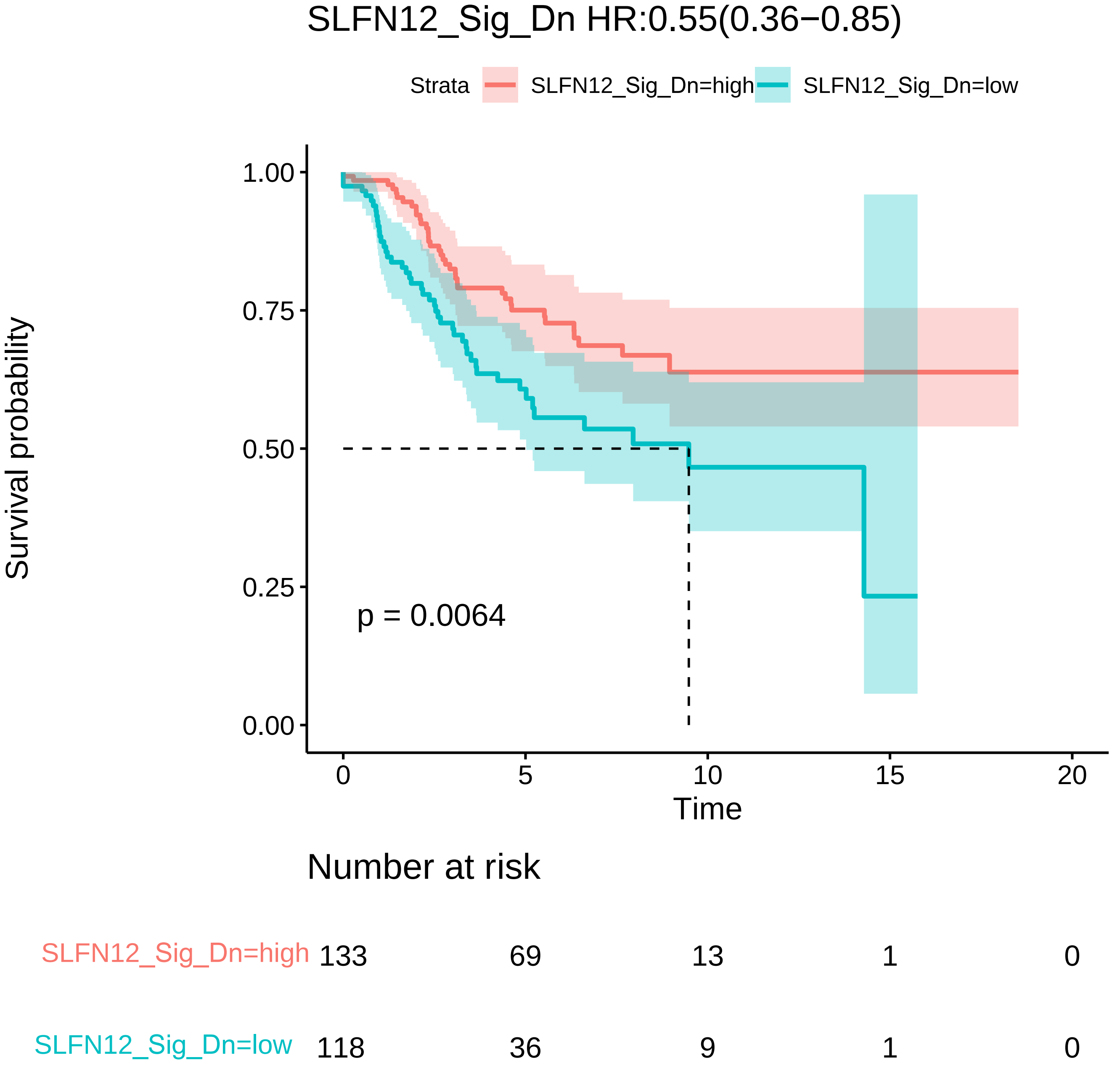

SLFN12_Sig_Dn HR:0.55(0.36−0.85)
Strata
SLFN12_Sig_Dn=high
SLFN12_Sig_Dn=low
Survival probability
1.00
0.75
0.50
0.25
0.00
p = 0.0064
0
5
10
15
20
Time
Number at risk
SLFN12_Sig_Dn=high 133 69 13 1 0
SLFN12_Sig_Dn=low 118 36 9 1 0

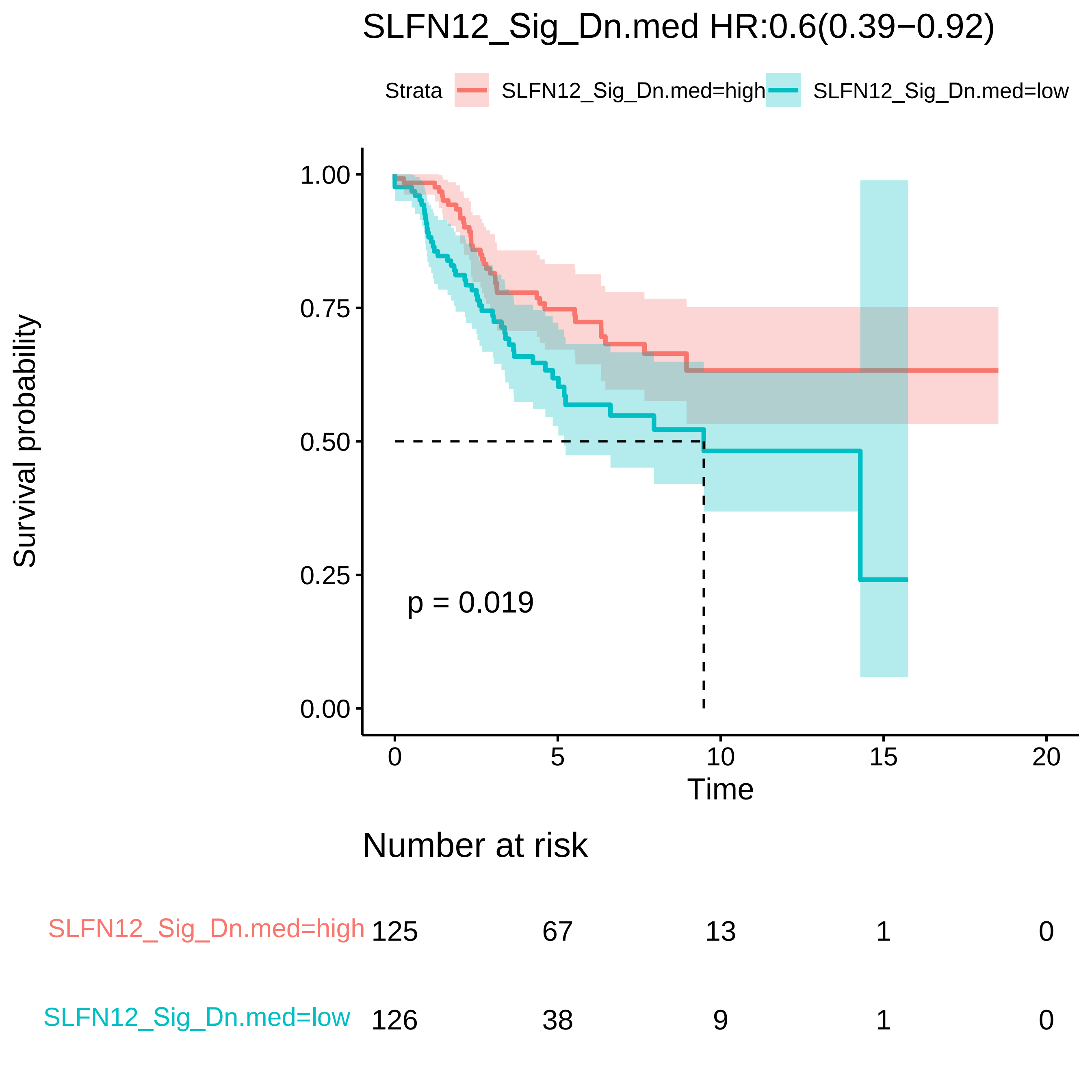

SLFN12_Sig_Dn.med HR:0.6(0.39−0.92)
Strata
SLFN12_Sig_Dn.med=high
SLFN12_Sig_Dn.med=low
Survival probability
1.00
0.75
0.50
0.25
0.00
p = 0.019
0
5
10
15
20
Time
Number at risk
SLFN12_Sig_Dn.med=high 125 67 13 1 0
SLFN12_Sig_Dn.med=low 126 38 9 1 0
