# Supplementary material for: Schlafen 12 Slows TNBC Tumor Growth, Induces Luminal Markers, and Predicts Favorable Survival
Source: Cancers (Basel). 2023 Jan 7;15(2):402. doi: 10.3390/cancers15020402 (PMC9856841; doi:10.3390/cancers15020402)
Supplement: Supplementary file 1 [file cancers-15-00402-s001.zip › 102422- Supplemental Figures Captions.pdf]

Supplemental Figure 1: Flowchart of study design.

Supplemental Figure 2: Cell type single cell analysis. A) KRT14 gene expression (Basal epithelial cell type gene) B) Epithelial cell type overall expression C) EPCAM gene expression (Epithelial cell type gene) D) ERBB2 gene expression (Epithelial cell type gene) E) EGFR gene expression (Epithelial cell type gene) F) Luminal epithelial cell type overall expression G) MUC1 gene expression (Luminal epithelial cell type gene) H) KRT8 gene expression (Luminal epithelial cell type gene) I) KRT18 gene expression (Luminal epithelial cell type gene). Log2 expression from 0 – 9.0. P-values given for significant clusters identified by single cell analysis.

Supplemental Figure 3: Hazards Ratios (HRs) based on median cutoffs of Data-1, Data-2, Data-3, and Data-4 for signatures A) SLFN12 B) SLFN12_All C) SLFN12_AllnoDir D) SLFN12_UpReg E) SLFN12_DnReg as defined in the methods. Horizontal bars represent the 95% CIs of HRs.

Supplemental Figure 4: Distribution, Maximally Selected Rank Statistics, and Kaplan Meier survival plots for Data-1. Optimal cuff-plots describes by solid lines and histogram present data for samples with levels higher (red) or lower (blue); the dashed lines present data for samples divided into two groups (higher-red or lower-blue) based on the "optimal cut-off" algorithm.

Supplemental Figure 5: Distribution, Maximally Selected Rank Statistics, and Kaplan Meier survival plots for Data-2. Optimal cuff-plots describes by solid lines and histogram present data for samples with levels higher (red) or lower (blue); the dashed lines present data for samples divided into two groups (higher-red or lower-blue) based on the "optimal cut-off" algorithm.

Supplemental Figure 6: Distribution, Maximally Selected Rank Statistics, and Kaplan Meier survival plots for Data-3. Optimal cuff-plots describes by solid lines and histogram present data for samples with levels higher (red) or lower (blue); the dashed lines present data for samples divided into two groups (higher-red or lower-blue) based on the "optimal cut-off" algorithm.

Supplemental Figure 7: Distribution, Maximally Selected Rank Statistics, and Kaplan Meier survival plots for Data-4. Optimal cuff-plots describes by solid lines and histogram present data for samples with levels higher (red) or lower (blue); the dashed lines present data for samples divided into two groups (higher-red or lower-blue) based on the "optimal cut-off" algorithm.

Supplemental Figure 8: A box plot describing the race difference A) SLFN12, B) SLFN12_Sig, C) SLFN12_Sig_NoDir, D) SLFN12_Sig_Up and E) SLFN12_Sig_Dn genes for White and Black patients for Data-2.

Supplemental Table 1: Dataset characteristics.

Supplemental Table 2: Global gene expression analysis of genes with $p < 0.05$. Genes are shown from smallest p-value to greatest p-value.

Supplemental Table 3: Global Gene Expression Analysis of Human Genes with $p < 0.05$ and FC >2 and FC < 0.5, and genes with FC > 4-fold. Genes are shown from largest raw p-value to smallest.

Supplemental Table 4: GSEA identified gene sets with minimum size 5 and nominal p-value < 0.05 based on Table 1's 1182 genes ($p < 0.05$) with A) upregulated based on human genes B) downregulated based on human genes. FWER p-value < 0.05 are highlighted in yellow.

Supplemental Table 5: GSEA identified gene sets with minimum size 50 and nominal p-value < 0.05 based on Table 1's 1182 genes ($p < 0.05$). FWER p-value < 0.05 are highlighted in yellow.

Supplemental Table 6: GSEA identified gene sets with minimum size 5 and nominal p-value < 0.05 based on Table 2's 549 genes with A) upregulated based on human genes B) downregulated based on human genes FWER p-value < 0.05 are highlighted in yellow.

Supplemental Table 7: GSEA identified gene sets with minimum size 50 and nominal p-value < 0.05 based on Table 2's 549 genes with A) upregulated based on human genes B) downregulated based on human genes FWER p-value < 0.05 are highlighted in yellow.

Supplemental Table 8: p-values from Single Cell analysis of C1 and R1.
