# Supplementary figures and images for: Schlafen 12 Slows TNBC Tumor Growth, Induces Luminal Markers, and Predicts Favorable Survival
Source: Cancers (Basel). 2023 Jan 7;15(2):402. doi: 10.3390/cancers15020402 (PMC9856841; doi:10.3390/cancers15020402)

Supplementary Figure-1

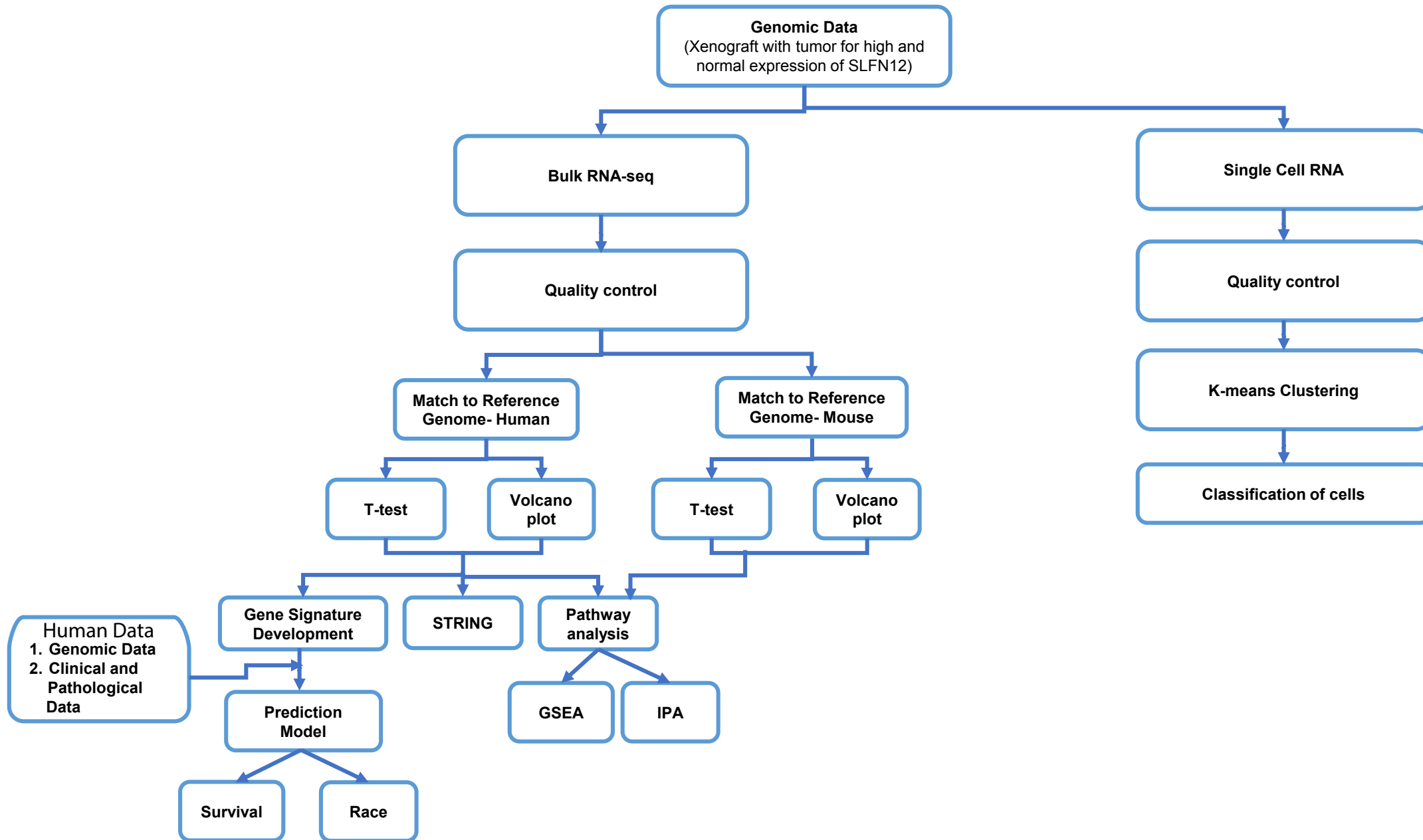

Supplement: Supplementary file 1 [file cancers-15-00402-s001.zip › Supplemental Figure 1 (1).pdf]

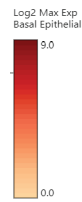

# Supplementary Figure-2

## KRT14

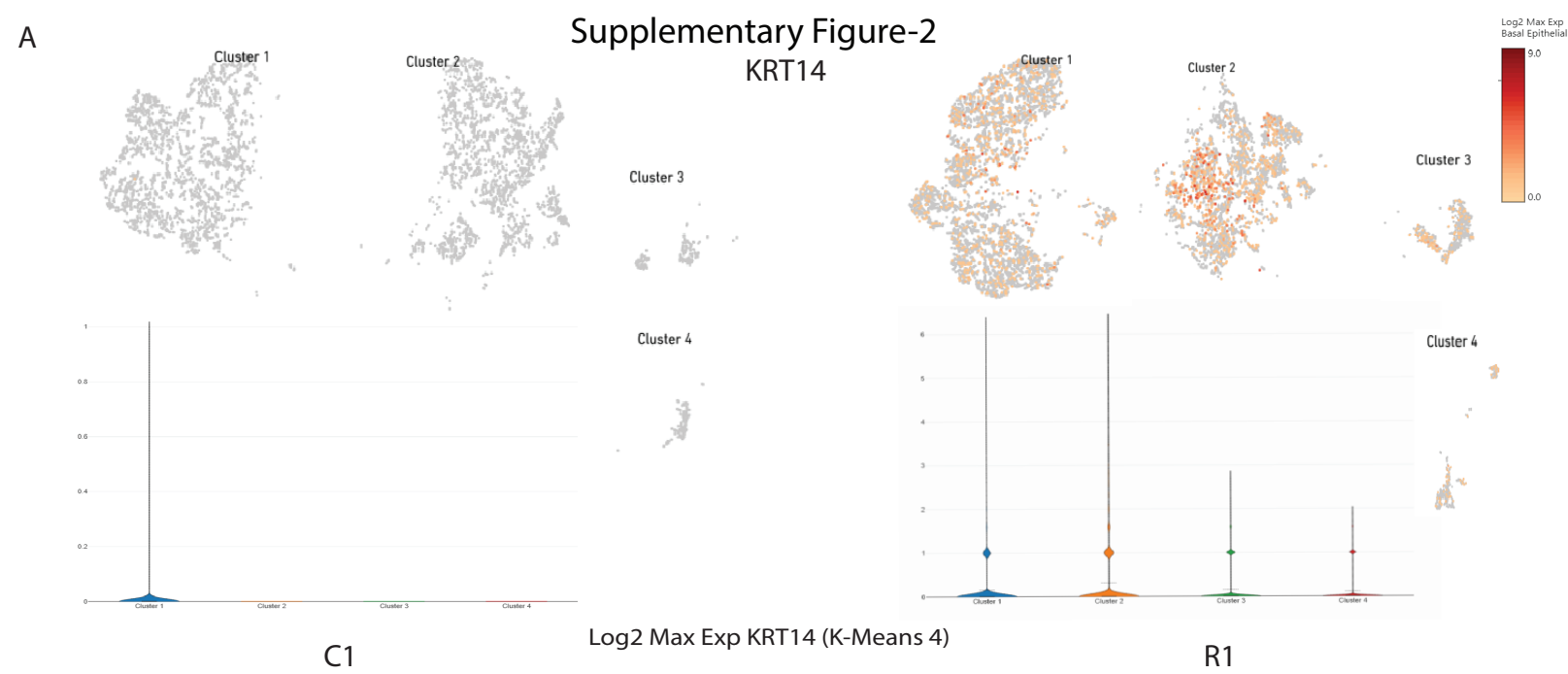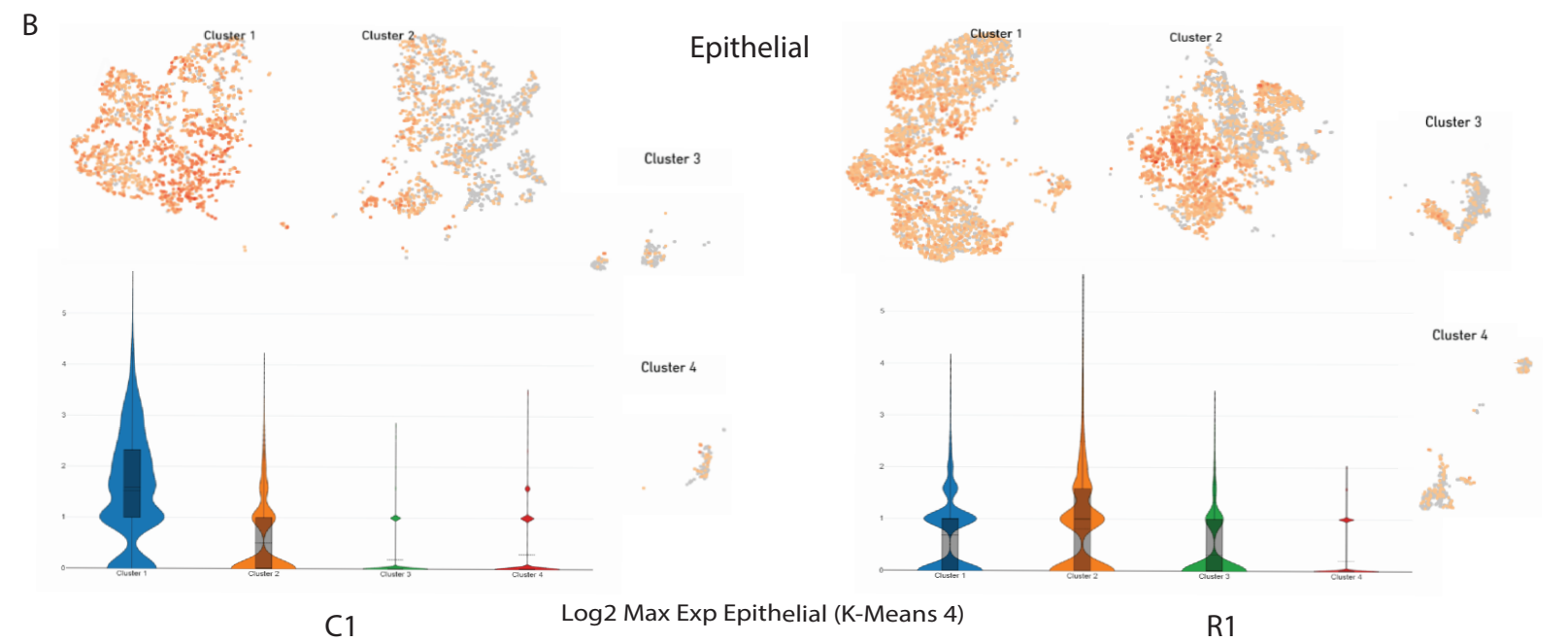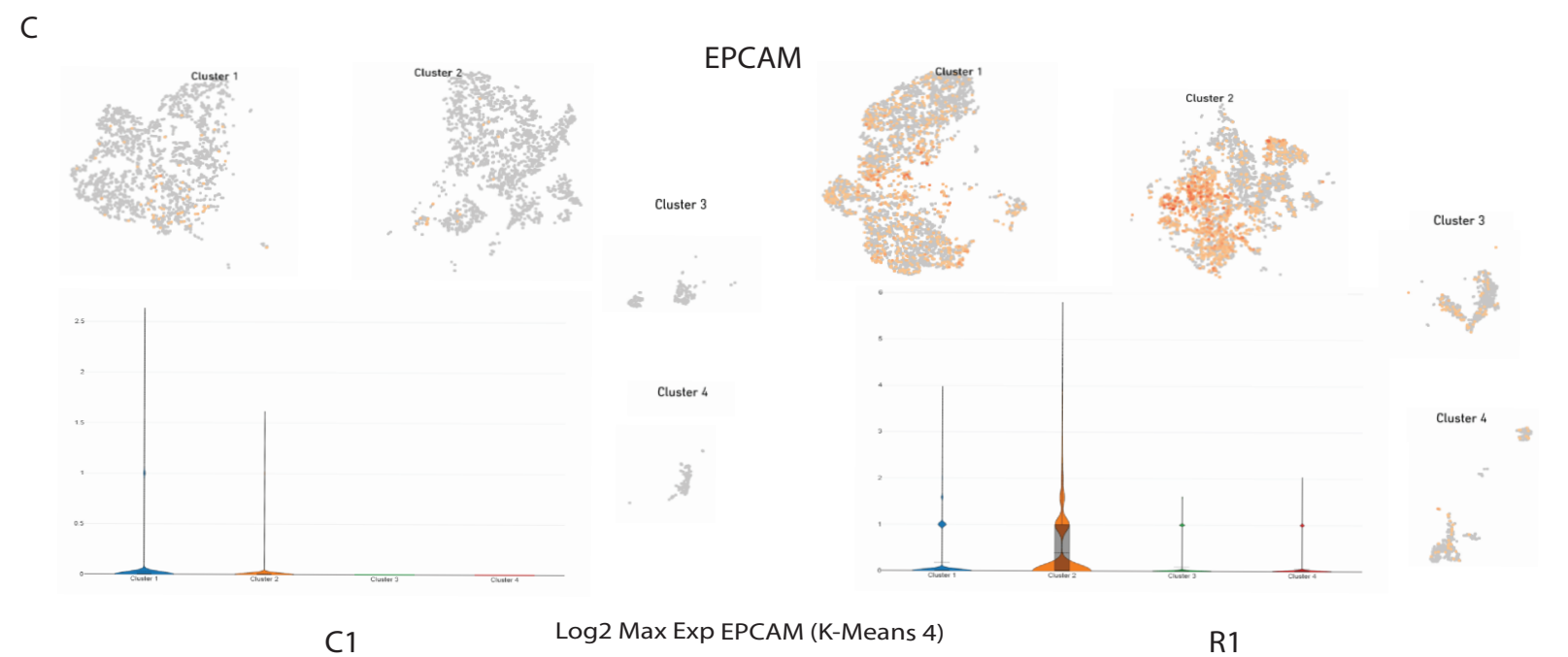

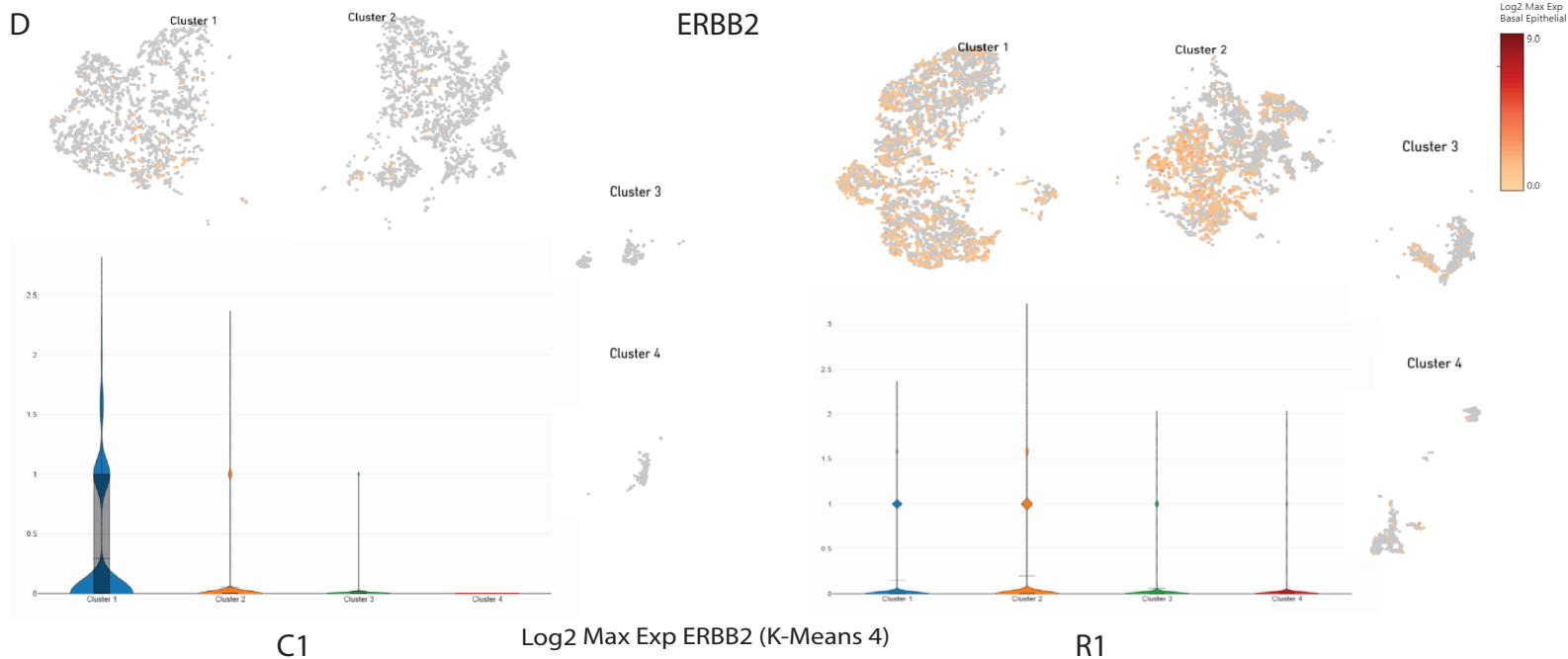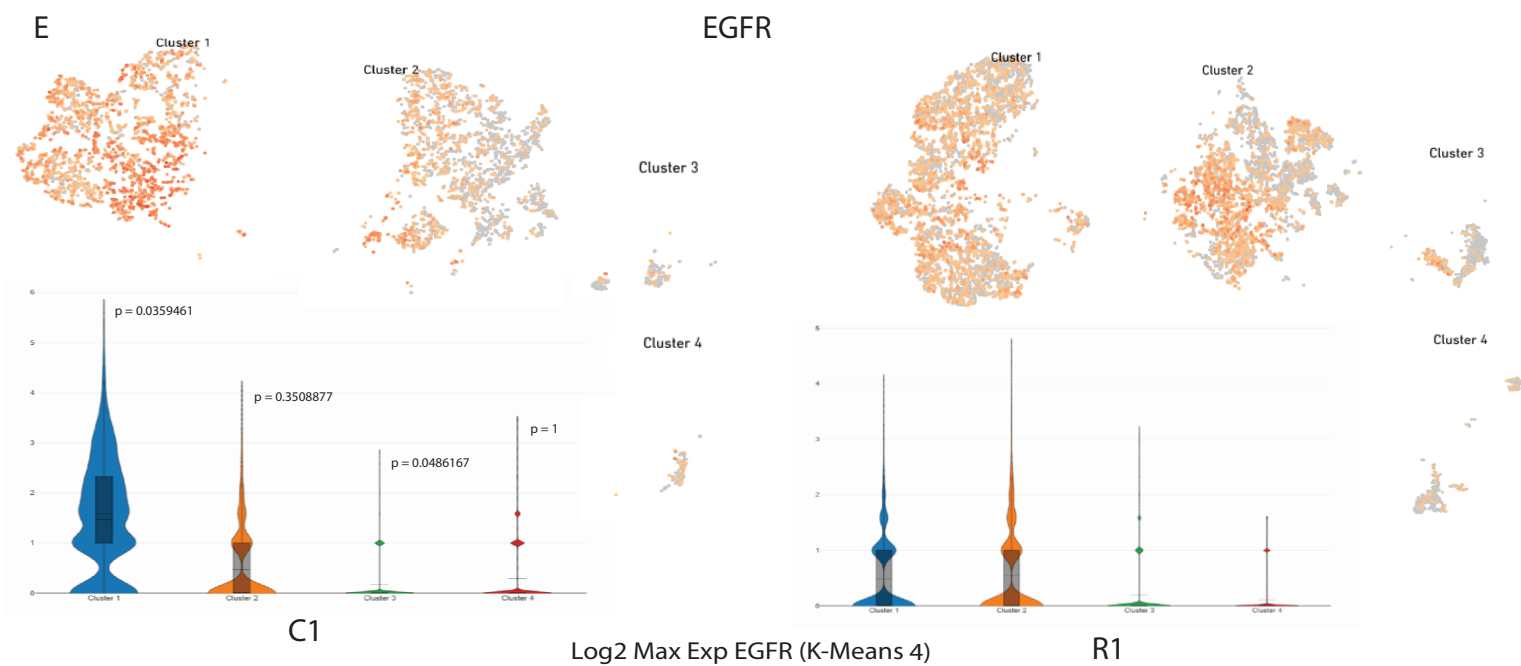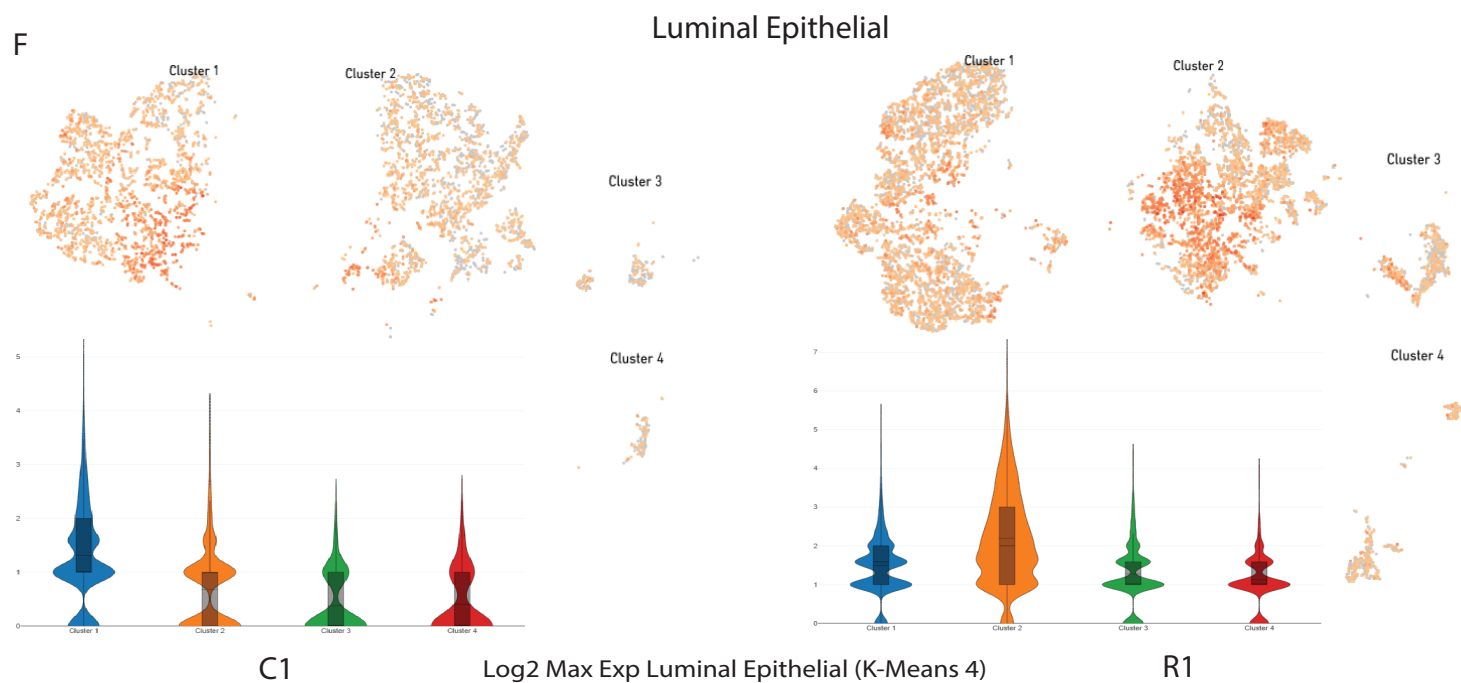

G

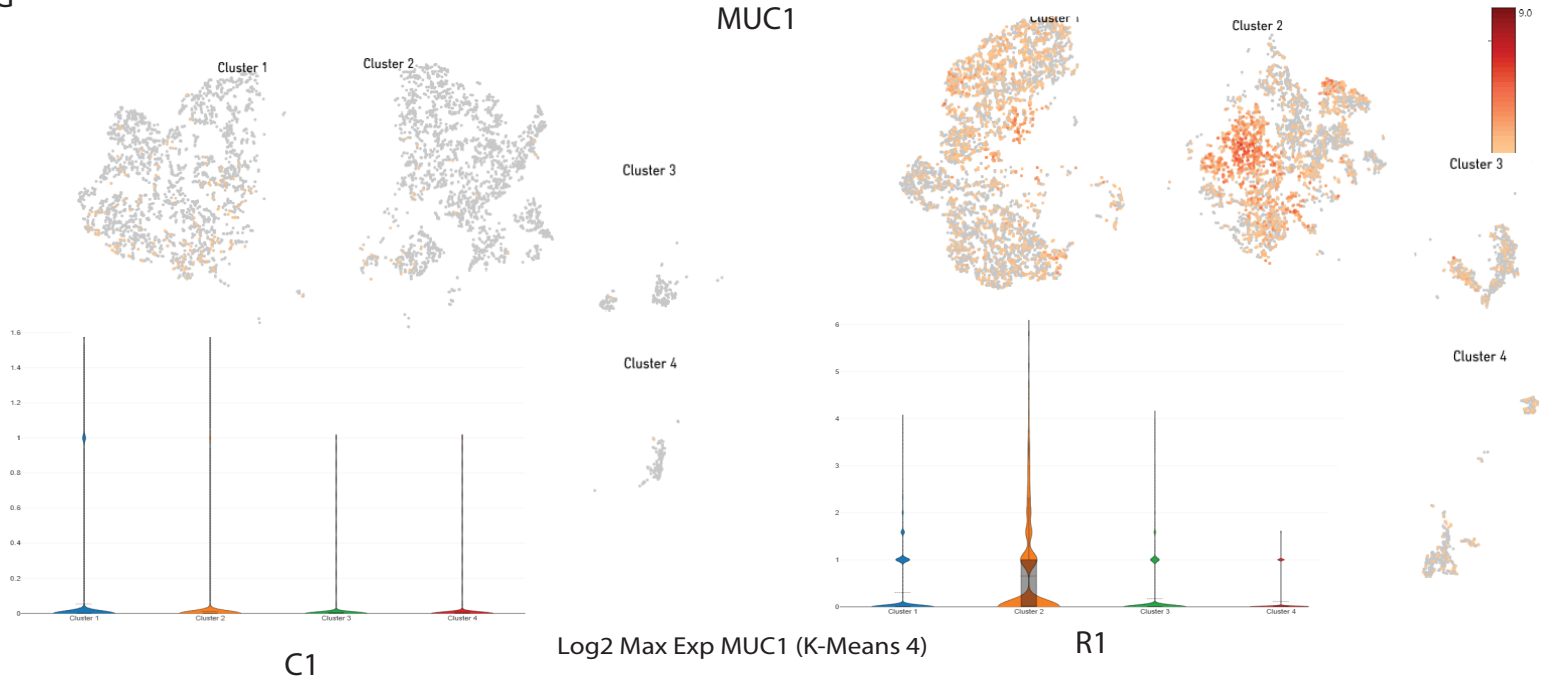

H

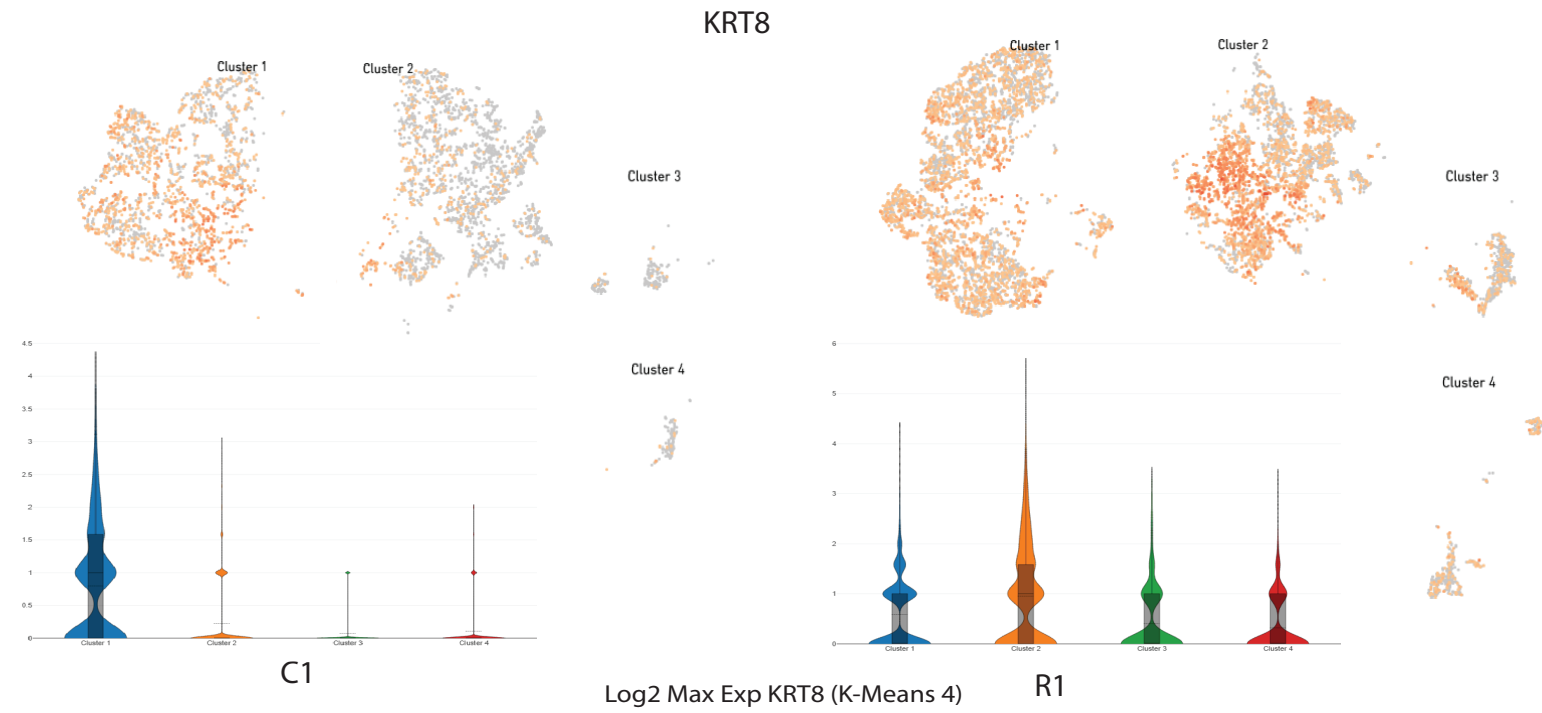

I

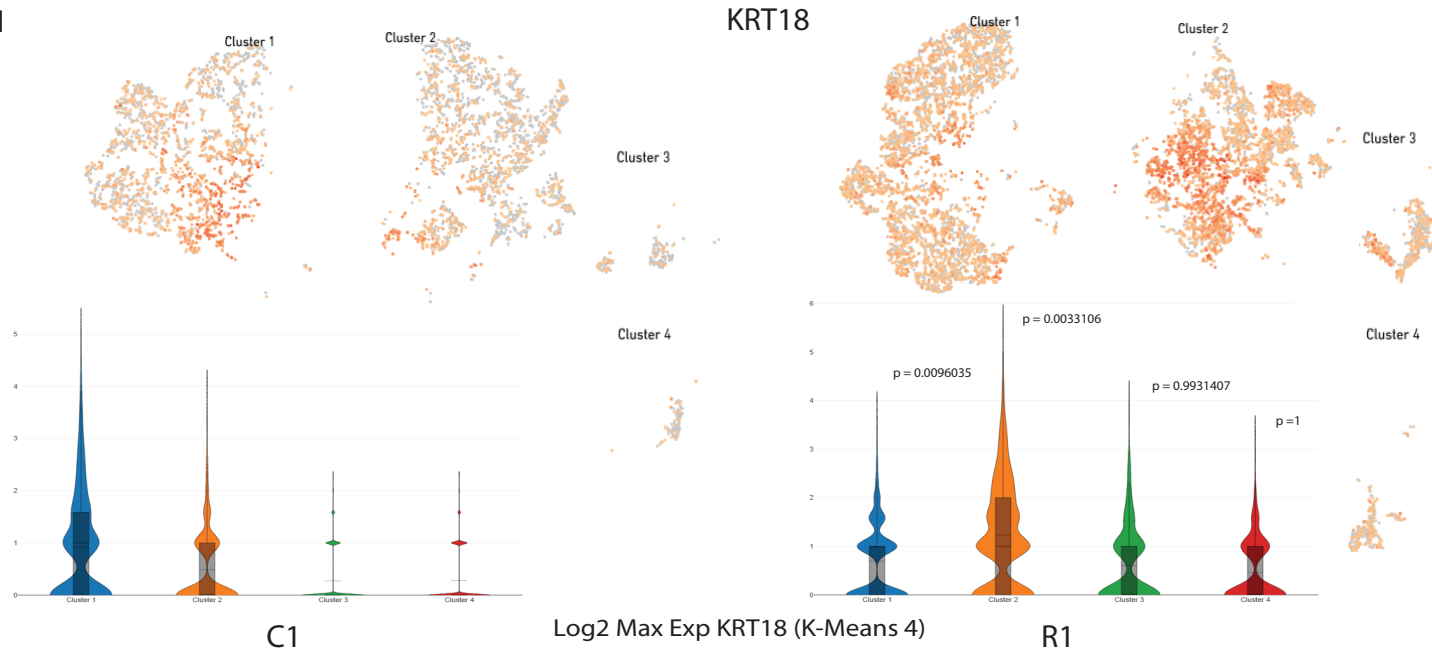

Supplement: Supplementary file 1 [file cancers-15-00402-s001.zip › Supplemental Figure 2 (1).pdf]

Supplementary Figure-8

Race 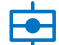 black 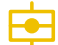 white

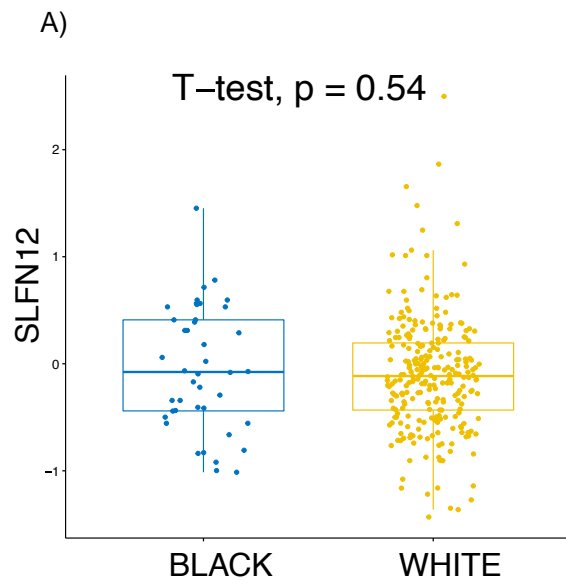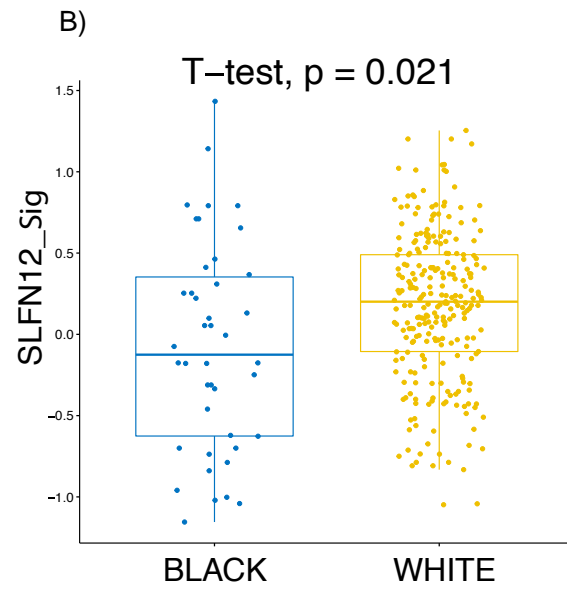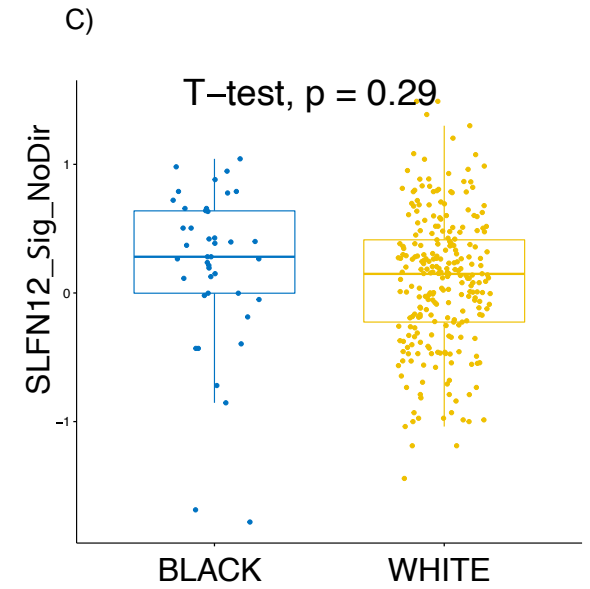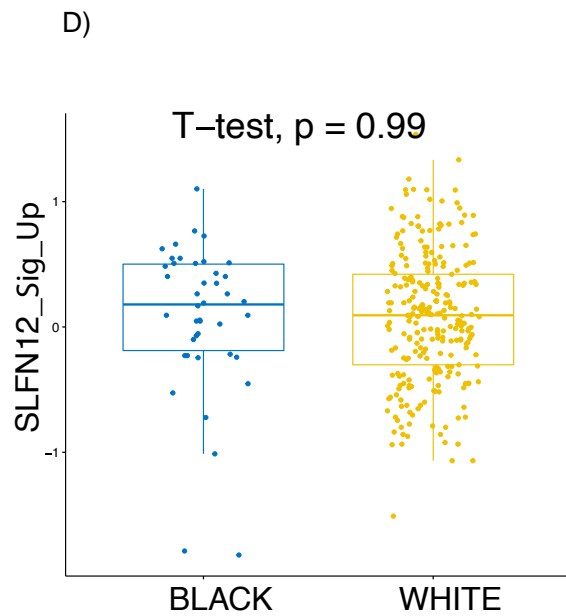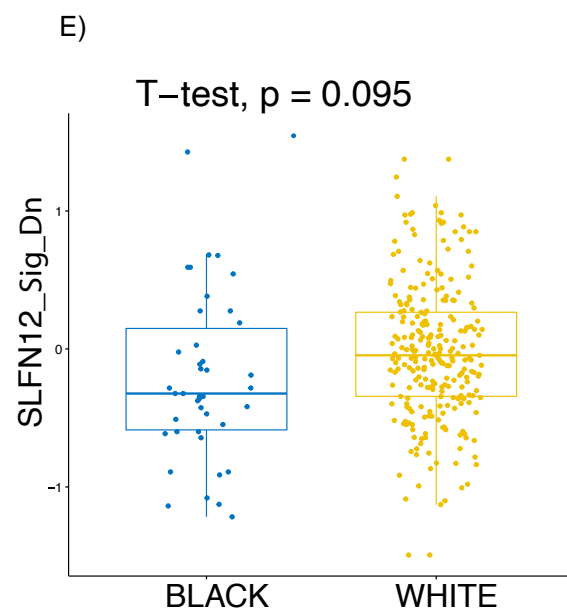

Supplement: Supplementary file 1 [file cancers-15-00402-s001.zip › Supplemental Figure 8 (1).pdf]
